# Precise homology-directed installation of large genomic edits in human cells with cleaving and nicking high-specificity Cas9 variants

**DOI:** 10.1093/nar/gkad165

**Published:** 2023-03-17

**Authors:** Qian Wang, Jin Liu, Josephine M Janssen, Manuel A F V Gonçalves

**Affiliations:** Department of Cell and Chemical Biology, Leiden University Medical Center, Einthovenweg 20, 2333 ZC Leiden, The Netherlands; Department of Cell and Chemical Biology, Leiden University Medical Center, Einthovenweg 20, 2333 ZC Leiden, The Netherlands; Department of Cell and Chemical Biology, Leiden University Medical Center, Einthovenweg 20, 2333 ZC Leiden, The Netherlands; Department of Cell and Chemical Biology, Leiden University Medical Center, Einthovenweg 20, 2333 ZC Leiden, The Netherlands

## Abstract

Homology-directed recombination (HDR) between donor constructs and acceptor genomic sequences cleaved by programmable nucleases, permits installing large genomic edits in mammalian cells in a precise fashion. Yet, next to precise gene knock-ins, programmable nucleases yield unintended genomic modifications resulting from non-homologous end-joining processes. Alternatively, in trans paired nicking (ITPN) involving tandem single-strand DNA breaks at target loci and exogenous donor constructs by CRISPR-Cas9 nickases, fosters seamless and scarless genome editing. In the present study, we identified high-specificity CRISPR-Cas9 nucleases capable of outperforming parental CRISPR-Cas9 nucleases in directing genome editing through homologous recombination (HR) and homology-mediated end joining (HMEJ) with donor constructs having regular and ‘double-cut’ designs, respectively. Additionally, we explored the ITPN principle by demonstrating its compatibility with orthogonal and high-specificity CRISPR-Cas9 nickases and, importantly, report that in human induced pluripotent stem cells (iPSCs), in contrast to high-specificity CRISPR-Cas9 nucleases, neither regular nor high-specificity CRISPR-Cas9 nickases activate P53 signaling, a DNA damage-sensing response linked to the emergence of gene-edited cells with tumor-associated mutations. Finally, experiments in human iPSCs revealed that differently from HR and HMEJ genome editing based on high-specificity CRISPR-Cas9 nucleases, ITPN involving high-specificity CRISPR-Cas9 nickases permits editing allelic sequences associated with essentiality and recurrence in the genome.

## Graphical Abstract

**Graphical Abstract:**
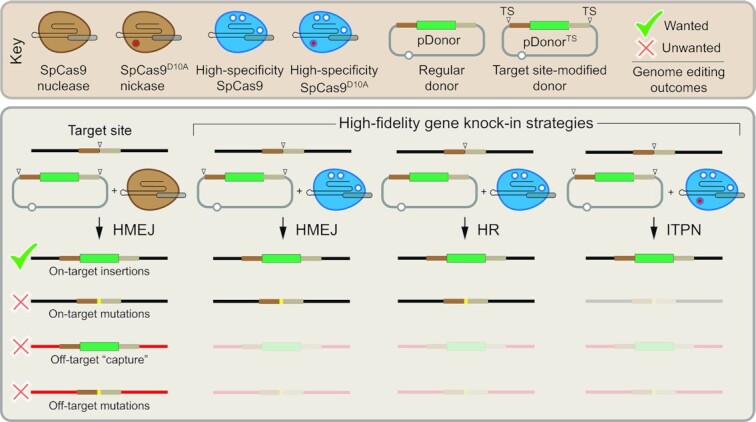
Genome editing strategies based on precision homology-directed gene targeting with high-specificity Cas9 variants minimize the complexity of bystander chromosomal effects, including off-target donor DNA insertions.

## INTRODUCTION

Owing to their versatility and potency, RNA-programmable nucleases derived from bacterial CRISPR-Cas9 adaptive immune systems are offering numerous opportunities in basic and applied research, including for the development of genetic therapies (1). Engineered CRISPR-Cas9 nucleases consist of a single guide RNA (gRNA) and a Cas9 enzyme with HNH and RuvC catalytic domains. In the growing set of CRISPR-based genome editing tools, prototypic *Streptococcus pyogenes* Cas9 (SpCas9) and its smaller orthologue *Staphylococcus aureus* Cas9 (SaCas9) nucleases are amongst the most robust (2,3). In cells, these ribonucleoprotein complexes start by engaging short genomic sequences named protospacer adjacent motifs (PAMs) that read NGG and NNGRRT (R = A or G) in the case of SpCas9 and SaCas9, respectively (3,4). Site-specific double-stranded DNA break (DSB) formation follows when, next to a PAM, lies a sequence (protospacer) complementary to the 5′ end of the gRNA (spacer). Specifically, after Cas9-PAM binding and local DNA unwinding, gRNA:DNA hybridization forms a R-loop whose progression from a PAM-proximal to PAM-distal direction eventually overcomes a conformational checkpoint barrier that triggers HNH translocation to the RuvC domain and DNA cleavage (4–6). Modulation of this conformational activation checkpoint by rationally designed or molecularly evolved Cas9 variants achieves heightened DNA mismatch discrimination and hence blunted off-target activities (7–11). As such, these mutant Cas9 enzymes constitute a critical resource for improving genome editing protocols, including those investigated in this study based on the targeted insertion of exogenous (donor) DNA into predefined chromosomal positions (12–14). Indeed, these genome editing approaches are appealing in that they permit introducing genomic modifications spanning from single base-pairs to whole transgene(s) and build on the straightforward designing of RNA-programmable Cas9 nucleases known to have high activities in mammalian cells (3,15–18).

Typically, CRISPR-Cas9 implementation of large genomic edits is accomplished by delivering donor DNA constructs tailored for site-specific DSB repair through ectopic non-homologous end joining (NHEJ) (19,20) or homology-directed repair (HDR) processes (12–14). The latter processes engage donor constructs favoring homologous recombination (HR) (12,13), microhomology-mediated end joining (MMEJ) (21) or, more recently, homology-mediated end joining (HMEJ) (22–24). MMEJ, HMEJ and HR donors have homology tracts (‘arms’) flanking the exogenous DNA whose sizes span approximately 20–50, 50–900 and 0.5–2.0 kb, respectively. In contrast, NHEJ-prone donors lack sequence homology to target DNA (19,20). In addition, diversely from HR donors, donors prone to NHEJ, MMEJ and HMEJ have a ‘double-cut’ design in that their targeting modules are surrounded by CRISPR-Cas9 cleaving sites (12–14). This design guarantees exogenous DNA release from construct backbones in cell nuclei, presumably fostering its exonucleolytic processing and target sequence annealing. Importantly, experimental evidence indicates that HR and HMEJ donors yield more precise and directional DNA insertions than their MMEJ and NHEJ counterparts (19,21,22). Additional data further shows that HMEJ donors can lead to higher genome editing frequencies than HR, MMEJ and NHEJ donors in mammalian cells and mouse blastocysts (22–24).

In this work, we start by identifying high-specificity CRISPR-Cas9 nucleases that once combined with donors strictly susceptible to HR or to HMEJ processes, trigger genome editing at levels similar to or higher than those obtained with regular CRISPR-Cas9 nucleases. Additional experiments established that high-specificity CRISPR-Cas9 complexes yield on-target and precise chromosomal insertion of large genetic payloads in human induced pluripotent stem cells (iPSCs). However, as expected, a substantial fraction of target alleles contained small insertions and deletions (indels) due to the prevalence of non-homologous end-joining (NHEJ) pathways over HDR in mammalian cells (25). Besides constituting substrates for mutations and chromosomal rearrangements (26,27), DSBs can lead to haploinsufficiency and cell fitness losses, e.g. when located in exons (28). Moreover, P53-dependent cell cycle arrest and apoptosis induced by CRISPR-Cas9-derived DSBs limits the efficacy of genome editing in stem cells (29,30), confounds genetic screens and, critically, creates selective pressure for the emergence of P53 and KRAS mutations which raises safety risks in stem cell therapies (31–33).

Cas9 proteins with either one of their nuclease domains disabled act as sequence-specific and strand-specific nucleases (nickases). Cas9 nickases are particularly appealing genome editing tools in that single-stranded DNA breaks (SSBs), or nicks, as such are not engaged by mutagenic end-joining DNA repair processes. Moreover, although chromosomal nicks constitute poor HDR stimuli, earlier research in our laboratory uncovered that tandem nicking at endogenous target sites and donor DNA constructs by native or engineered nickases elicits HDR-mediated genome editing (24,34). Examples concerning the application of such in trans paired nicking (ITPN) principles include mutation repair or installation (35–38), allele-specific gene editing (39,40), one-step biallelic gene editing (24,41), and one-step multiplexing gene knock-in or tagging (24,41) in various cell types, e.g. iPSCs, recessive dystrophic epidermolysis bullosa keratinocytes and organoids with regular or cancer traits (24,36,39,41).

Although nicks are mostly resolved in a conservative manner, they can nonetheless lead to DSBs if a replication fork advances through them and collapses (42). It is also known that the extent of baseline indel formation by Cas9 nickases vary in a locus sequence-dependent manner (43). Moreover, in previous studies from our laboratory, unbiased high-throughput genome-wide translocation sequencing (HTGTS) revelated that, albeit at low frequencies, SpCas9^D10A^:gRNA complexes can trigger translocations involving gRNA off-target sites and that using high-specificity SpCas9^D10A^:gRNA complexes can further reduce these unwanted genomic effects (28,44). Thus, towards expanding the application of ITPN genome editing and further minimizing nickase-derived DSBs at off-target sequences, we proceeded by investigating its compatibility with SaCas9 nickases and a set of high-specificity SpCas9 nickases. Finally, we found that in contrast to genome editing based on regular and high-specificity CRISPR-Cas9 nucleases, neither regular nor high-specificity CRISPR-Cas9 nickases provokes the P53-dependent DNA damage response (DDR) in human iPSCs.

## MATERIALS AND METHODS

### Cells

Human cervix carcinoma HeLa cells (American Type Culture Collection) and human embryonic kidney HEK293T cells were maintained in Dulbecco's modified Eagle's medium (DMEM; Thermo Fisher Scientific; Cat. No.: 41966029) supplemented with 5% fetal bovine serum (FBS). The generation, characterization and culture conditions of the human iPSCs used in this study (LUMC0020iCTRL) were detailed elsewhere (24,45). In brief, the iPSCs were kept in Essential 8 Medium (E8; Thermo Fisher Scientific; Cat. No.: A1517001) supplemented with 25 U ml^−1^ penicillin and 25 μg ml^−1^ of streptomycin (Thermo Fisher Scientific; Cat. No.: 15140122). Vitronectin Recombinant Human Protein (VTN-N; Thermo Fisher Scientific; Cat. No.: A14700) was applied for coating all the vessels used for iPSC culturing. The different cell types were tested for the absence of mycoplasma contamination and were cultured at 37ºC in humidified-air atmospheres with 5% CO_2_ (human iPSCs) or 10% CO_2_ (HeLa and HEK293T cells).

### Recombinant DNA

Standard recombinant DNA techniques were applied for the generation of the various expression plasmids. The assembly of isogenic expression constructs encoding the different SpCas9 nucleases and SpCas9^D10A^ nickases was described previously (44). Additionally, except for BA32_pU.CAG.SaCas9^N580A^, the generation of expression constructs encoding *S. aureus* SaCas9 nuclease and SaCas9^D10A^ nickase, was also detailed elsewhere (44). The annotated maps and nucleotide sequences of BA32_pU.CAG.SaCas9^N580A^, BB43_pmC.Donor^R5^, BB44_pmC.Donor^R5.TS^, AT13_pE.Donor^S1^, AA63_pE.Donor^S1.TS^, BA02_pE.Donor^CLYBL^, AZ64_pE.Donor^CLYBL.TS^, AD60_pEP.Donor^CLYBL^ and AD59_pEP.Donor^CLYBL.TS^ are available in pages 1–27 of the Supplementary Information. Detailed information about the *AAVS1*-targeting donor plasmids AX44_pS.Donor^S1^ (#100289), AX53_pS.Donor^S1.TS^ (#100290), AV11_pDonor.EP^S1^ (#100296) and AV09_ pDonor.EP^S1.TS^ (#100297), is available in an earlier work from our laboratory (24), and through the Addgene repository. Likewise for accessing information about AY22_pgRNA^R5^ (#100294) and AS11_gRNA^S1^ (#41818), encoding *CCR5*-specific and *AAVS1*-specific gRNAs, respectively. The generation of *OCT4*-targeting gRNA and donor constructs was described previously (28). Finally, specifics about the gRNA negative control constructs gRNA Cloning Vector (#41824) (18) and BPK2660 (#70709) (46), herein named, gRNA^Empty^ and Sa-gRNA^Empty^, respectively, can be equally obtained from Addgene. The target sequences of the *S. pyogenes* gRNAs and *S. aureus* Sa-gRNAs used in this work are indicated in Supplementary Table S1.

### DNA transfections

HeLa cells were transfected with the aid of 1 mg ml^−1^ 25 kDa linear polyethyleneimine (PEI, Polysciences) solution (pH 7.4) following the protocol described previously (44). The transfections of iPSCs were done by using the Lipofectamine Stem Transfection Reagent (Thermo Fisher Scientific; Cat. No.: STEM00003) according to the manufacturer's protocol. The cell numbers and the compositions of different transfection reactions are specified in Supplementary Tables S2–S22.

### Target-site genotyping

Genotyping assays assessing HDR-mediated knock-ins were performed through restriction fragment length analyses (RFLA) and junction PCR. RFLA assays were initiated by amplifying amplicons spanning the target sequences with the primers and PCR cycling conditions indicated in Supplementary Tables S23 and S24, respectively. Subsequently, 10 μl of the resulting PCR mixtures were incubated with 1 μl (10 U) of the restriction enzyme HindIII (Thermo Fisher Scientific; Cat. No.: ER0501) overnight at 37°C and were then analysed by agarose gel electrophoresis with the aid of a Gel-Doc XR+ system and the ImageLab 6.0.1 software (both from Bio-Rad). Undigested samples served as negative controls. The primer sequences and PCR cycling conditions used for junction PCR analyses are listed in Supplementary Tables S25 and S26, respectively.

### Flow cytometry

Nuclease- and nickase-trigged genome editing frequencies were determined by using a BD LSR II flow cytometer (BD Biosciences). Briefly, cells were harvested and resuspended in PBS supplemented with 0.5% bovine serum albumin (BSA) and 2 mM EDTA (pH 8.0). Parental non-transfected cells served as negative controls to establish the thresholds for background fluorescence. At least 10,000 viable single cells were acquired per sample. Data were analyzed with the aid of the FlowJo software (Tree Star; version 10.5.0). The genome editing frequencies were normalized to the initial transfection efficiencies as determined at 3 days post-transfection by using reporter-directed flow cytometry (Supplementary Figure S1).

### Amplicon deep sequencing

Mutagenic loads in cells edited through canonical HR versus ITPN were assessed using amplicon deep sequencing following the protocol detailed previously (44). The primers, cycling parameters and PCR mixtures used for the preparation of gene-specific and barcoded amplicons are indicated in Supplementary Tables S27–S30. Finally, amplicons were pooled in equal molar ratios and were subjected to next-generation Illumina MiSeq deep sequencing for obtaining 100,000 paired-end reads. The frequencies of on-target and off-target genomic indels were quantified with the aid of the CRISPResso2 software (47) after demultiplexing and adapter trimming of the paired-end MiSeq raw reads (R1 and R2 fastq files) with Cutadapt version 2.10 (48). The scripts applied for the CRISPResso2 analyses are available in the Supplementary Information.

### Off-target donor DNA insertion analysis

HeLa cells were transfected with constructs designed for HR, HMEJ or ITPN at *AAVS1* following the scheme specified in Supplementary Table S20. At 10 days post-transfection, the cells were exposed to puromycin (Thermo Fisher Scientific; Cat. No.: A1113803) at a final concentration of 1 μg ml^−1^ after which puromycin-resistant cell populations were harvested for genomic DNA extraction by using the DNeasy^®^ Blood & Tissue Kit according to the manufacturer's instructions (QIAGEN; Cat. No.: 69506). Donor DNA insertions at off-target *CPNE5* and at target *AAVS1* sequences were captured by junction PCR assays with the aid of Platinum™ SuperFi II DNA Polymerase (Thermo Fisher Scientific; Cat. No.: 12361010). Amplicons specific for *EGFP* served as internal controls. The PCR primers and cycling conditions used in these junction PCR assays are listed in Supplementary Tables S31 and S32, respectively. Afterwards, 10 μl of the *CPNE5* amplicons were incubated overnight at 37ºC with 10 U of the restriction enzymes EcoRI (Thermo Fisher Scientific; Cat. No.: ER0271) and PstI (Thermo Fisher Scientific; Cat. No.: ER0615) and were then analyzed by agarose gel electrophoresis with the aid of a Gel-Doc XR+ system and the ImageLab 6.0.1 software (both from Bio-Rad). In addition, indel formation at genomic target sequences was probed in cells edited through canonical HR, HMEJ and ITPN. To this end, the *AAVS1* target region was amplified using the PCR primers and cycling conditions indicated in Supplementary Tables S23-S24, and the resulting PCR products were then subjected to genotyping assays based on the mismatch-sensing T7 endonuclease I (T7EI). In brief, T7EI assays were initiated by subjecting *AAVS1* amplicons to the cycling parameters indicated in Supplementary Table S33 and, subsequently, 10-μl samples were treated with 0.5 μl (5 U) of the T7EI enzyme (New England Biolabs; Cat. No.: M0302) for 15 min at 37°C. T7EI-digested and undigested DNA was analyzed after agarose gel electrophoresis by using a Gel-Doc XR+ system and the ImageLab 4.1 software (both from Bio-Rad). Finally, Sanger sequencing of *AAVS1* amplicons followed by Tracking of Indels by Decomposition (TIDE) (49) was equally applied to probe indel formation in puromycin-resistant HeLa cell populations edited through ITPN.

### IncuCyte cell proliferation assay

iPSCs were seeded at a density of 2 × 10^3^ cells per well of 96-well plates coated with VTN-N. After approximately 16 h, the cells were exposed to 10 μM Nutlin-3a (Cayman Chemical; Cat. No.: 675576–98-4) or to the vehicle DMSO for 3 days. Cell proliferation activity was monitored in the IncuCyte live-cell imaging system and real-time analyzed by the IncuCyte software (Essen BioScience).

### Cell viability assay

The colorimetric MTS (3-(4,5-dimethylthiazol-2-yl)-5-(3-carboxymethoxyphenyl)-2-(4-sulfophenyl)-2H-tetrazolium) assay was carried out for assessing iPSC viability upon Nutlin-3a treatment. In brief, iPSCs were seeded at a density of 2 × 10^3^ cells per well of 96-well plates coated with VTN-N. The next day, the cells were exposed to 2 μM Nutlin-3a, 10 μM Nutlin-3a or to DMSO vehicle for 6, 24, 48 and 72 h. Mock-treated iPSCs served as negative controls. At each of the indicated timepoints, 20 μl of MTS solution (Promega; Cat. No.: G3581) were directly added to each sample and the resulting mixtures were then incubated for 1 h at 37°C. The absorbance at OD_490_ nm was measured with the aid of a multimode plate reader (PerkinElmer VICTOR^TM^ X3).

### Apoptosis analysis

The frequency of apoptotic iPSCs was quantified by using an eBioscience™ Annexin V Apoptosis Detection Kit FITC (Thermo Fisher Scientific; Cat. No.: 88–8005-72) following the manufacturer's recommendations. In brief, iPSCs were plated at a density of 1 × 10^5^ cells per well of 12-well plates coated with VTN-N. After a 2-day incubation period, the cells were treated with 10 μM Nutlin-3a for 4 h, 6 h, and 8 h. Cells exposed to the protein kinase inhibitor Staurosporine (Cell Signaling Technology; Cat. No: 9953S) or to DMSO vehicle served as positive and negative controls for apoptosis, respectively. At the indicated timepoints, the iPSCs were harvested and resuspended in 1× Binding Buffer. Subsequently, each cell suspension was incubated for 10 min at room temperature with 5 μl of Annexin V conjugated to the FITC fluorochrome. After washing twice with 1× Binding Buffer, the cells were resuspended in 200 μl of 1× Binding Buffer containing 10 μl of 20 μg ml^−1^ propidium iodide (PI). Finally, the frequency of apoptotic iPSCs was determined by using a BD LSR II flow cytometer (BD Biosciences) with the acquired data being analysed with the aid of the FlowJo software (Tree Star; version 10.5.0).

### Real-time quantitative PCR (RT-qPCR)

RT-qPCR was applied for assessing the activation of the P53-dependent DDR. Total RNA was extracted by using the NucleoSpin RNA Kit according to the manufacturer's instructions (Macherey Nagel; Cat. No.: 740955). Equal amounts of isolated RNA quantified with a NanoDrop apparatus were reverse transcribed by using the RevertAid RT Reverse Transcription Kit (Thermo Fisher Scientific; Cat. No.: K1691). In brief, 500–1000 ng of RNA were incubated with 0.5 μl of 100 μM random hexamer primers and 0.5 μl of 100 μM Oligo(dT)_18_ primers in 12-μl reaction volumes at 65°C for 5 min followed by 2-min incubations at 4°C. Subsequently, 1 μl of 20 U μl^−1^ RiboLock RNase Inhibitor, 1 μl of 200 U μl^−1^ RevertAid H Minus M-MuLV Reverse Transcriptase, 2 μl of 10 mM dNTP Mix and 4 μl of 5× Reaction Buffer, were directly added to each sample and the resulting mixtures were then incubated for 5 min at 25°C followed by 1 h at 42°C. Next, after deactivating the reverse transcriptase by heating at 70°C for 5 min, 1 μl of the synthesized cDNA samples was subjected to qPCR using the iQ™ SYBR^®^ Green Supermix (Bio-Rad; Cat. No.: L010171C) for determining the expression of *TP53* and of the canonical P53-responsive genes *P21, FAS*, *PUMA* and *MDM2* as well as of the P53 non-responsive gene *HPRT1*. Housekeeping *GAPDH* transcripts were targeted to serve as references for expression normalization. The specificity of each primer pair was predicted by *in silico* BLAST screens and then validated by assessing the melting profiles. Information on target sequences, qPCR primers, mixture components and reaction conditions are indicated in Supplementary Tables S34 and S35. The CFX Connect Real-Time PCR Detection System (Bio-Rad) was applied for the detection of signal outputs and the relative expression levels were calculated through the 2^−ΔΔCt^ method with the aid of the Bio-Rad CFX Manager software (version 3.1). The GraphPad Prism software (version 9.3.1) was applied for the statistical analyses of the resulting RT-qPCR datasets.

### Western blotting

Laemmli buffer consisting of 8.0% glycerol, 3% sodium dodecyl sulphate (SDS) and 200 mM Tris–HCl (pH 6.8) was applied for lysing human iPSCs and HEK293T cells. Afterwards, equal amounts of protein were separated by SDS-polyacrylamide gel electrophoresis (SDS-PAGE) and transferred onto 0.45 μm polyvinylidene difluoride (PVDF) membranes (Merck Millipore; Cat. No.: IPVH00010). After 1 h blocking at room temperature in Tris-buffered saline (TBS) containing 5% non-fat dry milk and 0.1% Tween 20 (TBST), the membranes were incubated overnight at 4°C with the respective primary antibodies, i.e. anti-P21 (Sigma-Aldrich; Cat. No.: 05–655; 1:1000 dilution) and anti-GAPDH (Merck Millipore; Cat. No.: MAB374; 1:1000 dilution). Subsequently, the membranes were washed with TBST thrice and probed with the secondary anti-mouse IgG antibody (Sigma-Aldrich; Cat. No.: NA931V; 1:5000 dilution) at room temperature for 2 h. The Clarity™ Western ECL Substrate (Bio-Rad; Cat. No.: 1705060) and the ChemiDoc Imaging System (Bio-Rad; Cat. No.: 17001402) were applied for signal detection.

### *OCT4* gene tagging

Human iPSCs were transfected with constructs designed for tagging *OCT4* through HR, HMEJ or ITPN following the scheme indicated in Supplementary Table S22. At 2 days post-transfection, the iPSCs were transferred to wells of 6-well plates (Greiner Bio-One) coated with VTN-N and, upon reaching approximately 50% confluency, were exposed to E8 Medium containing 0.5 μg ml^−1^ puromycin. The resulting puromycin-resistant iPSC colonies were identified by using the Leukocyte Alkaline Phosphatase Kit following the manufacturer's instructions (Sigma-Aldrich; Cat. No.: 86R-1KT). Additionally, the puromycin-resistant iPSCs were further expanded for quantifying the frequency of cells expressing OCT4::EGFP after transduction with a lentiviral vector coding for the bacteriophage P1 Cre recombinase (LV.Cre) (28,44) at a multiplicity-of-infection (MOI) of 20 vector particles per cell. The quantification of OCT4::EGFP-positive iPSCs was carried out with the aid of a BD LSR II flow cytometer (BD Biosciences).

### iPSC differentiation

The *in vitro* spontaneous differentiation of iPSCs into mesoderm cells was described elsewhere (44). In brief, OCT4::EGFP^+^ iPSCs were dissociated into large cell clumps and incubated in suspension on low-attachment plates for a period of 24 h. Afterwards, the cell clumps were replated on glass coverslips coated with Vitronectin. After two days in culture, the regular growth medium was replaced by differentiation medium, i.e. DMEM/F12 (Gibco; Cat. No. 31331–028) containing 20% FBS. The differentiation medium was replenished every 2–3 days during the following 3 weeks. The differentiation of OCT4::EGFP^+^ iPSCs into ectoderm and endoderm cells was carried out with the aid of the STEMdiff™ Trilineage Differentiation Kit (STEMCELL Technologies; Cat. No. 05230) following the manufacturer's recommendations. Confocal immunofluorescence microscopy analyses were carried out for detecting the indicated lineage markers specific for mesoderm, ectoderm and endoderm germ layers (Supplementary Table S36).

### Confocal immunofluorescence microscopy

OCT4::EGFP^+^ iPSC populations were fixed with 4% paraformaldehyde (PFA), permeabilized with 0.5% Triton X-100 in TBS pH 7.6 (50 mM Tris–HCl pH 7.6; 150 mM NaCl), and blocked with a blocking solution consisting of TBS, Triton X-100, 2% BSA and 0.1% sodium azide. Afterwards, the cells were incubated with the corresponding primary antibodies and after thorough washes in TBS were exposed to the fluorochrome-conjugated secondary antibodies indicated in Supplementary Table S36. The specimens were mounted in the ProLong Gold Antifade Mounting reagent (Thermo Fisher Scientific; Cat. No.: P36931), and the images were captured by using an upright Leica SP8 confocal microscope equipped with Leica hybrid detectors, HyD (Leica Microsystems) and analyzed the with the aid of the LAS X software.

### Statistical analyses

Statistical analyses on data obtained from at least three biological replicates were performed with the GraphPad Prism software (version 9.3.1). Information on statistical parameters and tests used are specified in the figure legends

## RESULTS

### Functional screens identify Cas9 variants with improved performance over regular Cas9 for HR and HMEJ genome editing

**Figure 1.**
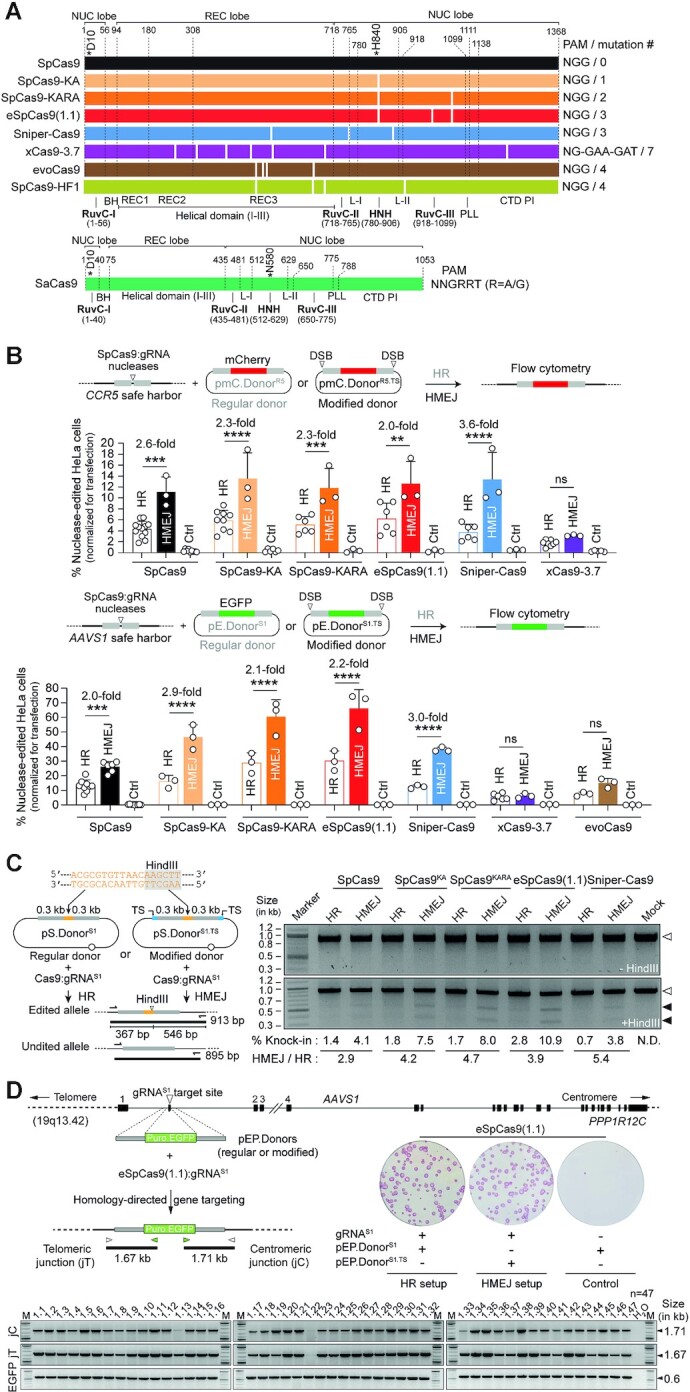
Testing DSB-dependent genome editing using regular versus high-specificity SpCas9 nucleases. (**A**) Diagrams of engineered Cas9 nucleases derived from *S. pyogenes* and *S. aureus* type II CRISPR systems. Protein domains and mutation positions are marked by dashed and white lines, respectively. HNH, histidine-asparagine-histidine nuclease domain; RuvC, RuvC-like nuclease domain composed of a tripartite assembly of RuvC-I, -II and -III. The HNH and RuvC domains in the nuclease lobe cut the target and non-target DNA strands, respectively. L-I and L-II, linker region I and II, respectively. BH, Arginine-rich bridge helix; CTD, C-terminal domain in which the PAM-interacting motif (PI) is integrated; NUC and REC, nuclease and recognition lobes, respectively; PLL, phosphate lock loop. Asterisks mark residues D10 and H840 crucial for RuvC and HNH catalytic activities, respectively. (**B**) Genome editing based on donors prone to canonical HR and HMEJ upon high-specificity SpCas9 delivery. Nuclease-dependent genome editing frequencies in HeLa cells transfected with the depicted reagents targeting *CCR5* and *AAVS1* were quantified by reporter-directed flow cytometry at 17 days post-transfection (top and bottom graphs, respectively). HeLa cells exposed to corresponding Cas9 nucleases and regular donor plasmids in the absence of locus-specific gRNAs served as negative controls. Data are plotted as mean ± SD of at least 3 independent biological replicates. Significant differences between the indicated datasets were determined by two-way ANOVA followed by Šidák's multiple comparisons tests; ****P < 0.0001, ***0.0001 < *P*< 0.001, **0.001 < *P*< 0.01; *P*> 0.05 was considered non-significant (ns). (**C**) Genotyping assay assessing HDR-mediated restriction site knock-ins. Regular pS.Donor^S1^ and modified pS.Donor^S1.TS^ constructs, designed to introduce a HindIII recognition site at *AAVS1* through HR and HMEJ processes, respectively, were transfected into HeLa cells together with plasmids expressing SpCas9 nucleases and gRNA^S1^. The HindIII polymorphism is detected by restriction-fragment length analysis (RFLA) of amplicons covering the target site (left panel). RFLA products diagnostic for unedited and edited *AAVS1* alleles retrieved from HeLa cells exposed to the indicated reagents were measured through densitometry and are marked with open and closed arrowheads, respectively (right panel). (**D**) Genotyping assay assessing HDR-mediated transgene knock-ins. Regular pEP.Donor^S1^ and modified pEP.Donor^S1.TS^ plasmids, tailored for inserting the live-cell selectable marker Puro^R^.EGFP at *AAVS1* via HR and HMEJ processes, respectively, were transfected into iPSCs together with constructs expressing eSpCas9(1.1):gRNA^S1^ complexes. HDR-derived gene knock-ins were identified by junction PCR analysis of randomly selected iPSC clones engineered through pEP.Donor^S1^ and eSpCas9(1.1):gRNA^S1^ delivery. Puromycin-resistant iPSC colonies were identified by staining for the pluripotency marker alkaline phosphatase.

Gene targeting (knock-in) into safe harbor loci in single or multiplexing formats leverages and broadens synthetic biology and genetic therapy efforts (50,51). Hence, to test the performance of the different gene knock-in tools and strategies, the commonly used prototypic safe harbor loci *AAVS1* and *CCR5* were selected, together with the more recently characterized *CLYBL* locus, as endogenous target sequences (52,53). We started by comparing the performance of wild-type SpCas9:gRNA complexes with those of a representative panel of high-specificity SpCas9 variants for DSB-dependent genome editing using regular and target site-modified plasmid donors designed for HR and HMEJ, respectively. This panel consists of SpCas9 variants SpCas9-KA (8), SpCas9-KARA (8), eSpCas9(1.1) (8), Sniper-Cas9 (11), xCas9-3.7 (10), evoCas9 (9) and SpCas9-HF1 (7) (Figure 1A). Thus, cervical carcinoma HeLa cells were transfected with regular HR or modified HMEJ donors each mixed with isogenic constructs expressing individual nucleases and canonical gRNAs specific for *CCR5* or *AAVS1* acceptor genomic sequences (Figure 1B, top and bottom graphs, respectively). Of notice, in contrast to gRNAs with extended spacers and/or non-hybridizing 5’ guanines, canonical *S. pyogenes* gRNAs with 20-nucleotide spacers fully complementary to protospacer DNA do not hinder high-specificity SpCas9 nuclease activities (9,11,44,54,55). After a 2-week sub-culturing period, to eliminate expression from episomal donor templates, DSB-dependent genome editing frequencies were determined by reporter-directed flow cytometry (Figure 1B). These experiments revealed that Sniper-Cas9 together with the nuclease sub-set formed by the single K848A, double K848A/R1060A and triple K848A/K1003A/R1060A mutants SpCas9-KA, SpCas9-KARA and eSpCas9(1.1), respectively, yielded DSB-dependent genome editing levels as high as or higher than those achieved by the parental SpCas9 nuclease (Figure 1B and Supplementary Figure S2). Indeed, frequencies reached with HR and HMEJ templates at *AAVS1* were, respectively, 13.22 ± 3.92% and 26.17 ± 3.66% when delivering SpCas9 versus 30.18 ± 6.78% and 66.14 ± 12.8% when introducing eSpCas9(1.1) (Supplementary Figure S3). Moreover, similarly to experiments using SpCas9, modified HMEJ-prone donors outperformed donors strictly susceptible to HR when combined with Sniper-Cas9, SpCas9-KA, SpCas9-KARA and eSpCas9(1.1) (Figure 1B). In contrast, genome editing frequencies induced by xCas9-3.7 and evoCas9 were lower than those triggered by SpCas9, with differences between HR and HMEJ donors not reaching significance (Figure 1B). Further experiments revealed that eSpCas9(1.1) outperformed SpCas9-HF1 at *AAVS1* and *CLYBL*, with the highest differences in genome editing levels reached by these two nucleases observed at the latter locus (Supplementary Figure S4). Specifically, DSB-dependent genome editing frequencies achieved with HR and HMEJ templates at *CLYBL* were, respectively, 0.86 ± 0.18% and 7.36 ± 2.44% when using SpCas9-HF1 versus 8.82 ± 1.52% and 54.15 ± 4.71% when deploying eSpCas9(1.1) instead (Supplementary Figure S4).

Subsequently, independent assays based on tracing polymorphism knock-ins in HeLa cells by restriction fragment length analysis (RFLA) (Figure 1C); and screening transgene knock-ins in randomly isolated iPSC colonies (*n* = 47) by junction PCR assays established HDR-mediated gene targeting in cells exposed to high-specificity nucleases and matched donor constructs (Figure 1D). Moreover, in agreement with the experiments using *AAVS1*-targeting reporter constructs (Figure 1B, bottom graph), the RFLA assay detected the highest DSB-dependent genome editing levels when delivering the high-specificity nucleases SpCas9-KA, SpCas9-KARA and eSpCas9(1.1) together with HMEJ donor templates (Figure 1C).

**Figure 2.**
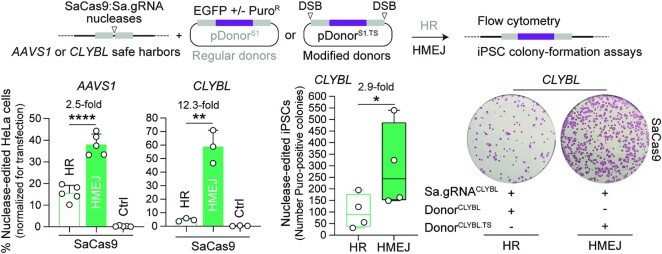
Genome editing combining plasmid donors with regular HR or modified HMEJ templates and orthogonal SaCas9 complexes. SaCas9-dependent genome editing at *AAVS1* and *CLYBL* loci in HeLa cells using EGFP-encoding donors, and at *CLYBL* in iPSCs using Puro^R^.EGFP-encoding donors was determined by reporter-directed flow cytometry and colony-formation assays, respectively. The latter assays detected the pluripotency marker alkaline phosphatase to identify puromycin-resistant iPSCs. Controls consisted of cells exposed to regular donor plasmids and SaCas9 nucleases with non-targeting gRNAs. Data are presented as mean ± SD of at least three independent biological replicates. Significant differences between the indicated datasets were calculated by two-tailed unpaired Student's *t* tests (left and middle graphs) and two-tailed paired ratio *t* test (right graph); ****P < 0.0001, **0.001 < *P*< 0.01, *0.01 < *P*< 0.05.

Towards expanding the scope of HR- and HMEJ-based genome editing, we next tested the SaCas9 nuclease (Figure 1A) together with *AAVS1*-targeting donors in HeLa cells or with *CLYBL*-targeting donors in HeLa cells and iPSCs (Figure 2). DSB-dependent genome editing frequencies were measured by flow cytometry of EGFP-expressing HeLa cells or by colony-formation assays based on puromycin selection and alkaline phosphatase staining of Puro^R^.EGFP-expressing iPSCs (Figure 2). In line with the experiments using SpCas9 (Figure 1B and Supplementary Figure S4), donor constructs prone to HMEJ yielded higher DSB-dependent genome editing frequencies than donors strictly susceptible to HR, independently of the cell type or genomic target region probed (Figure 2). In HeLa cells, this difference was most noticeable at *CLYBL* with HR- and HMEJ-prone donors resulting in SaCas9-edited cell frequencies of 4.77 ± 1.16% and 58.8 ± 12.19%, respectively (Figure 2).

Together, these experiments have identified Cas9 nucleases whose high specificities and activities turn them into preferable tools for DSB-dependent genome editing approaches. In addition, these data validate a versatile set of CRISPR reagents and matched HR- and HMEJ-tailored donor constructs for safe harbour targeting in human cells.

### Functional screens identify high-specificity Cas9 variants compatible with in trans paired nicking

By enhancing otherwise inefficient SSB-dependent HR, ITPN constitutes a valuable approach for seamless chromosomal installation of large DNA segments in eukaryotic cells (24). Moreover, owing to its scarless character, ITPN is particularly useful for achieving allele-specific editing (39–41), minimizing haploinsufficiency, or for editing repetitive or essential genomic tracts (28). In addition, ITPN has been applied for one-step biallelic and multiplexing DNA editing and for clonal screening-free generation of model cells and organoids (24,41).

**Figure 3.**
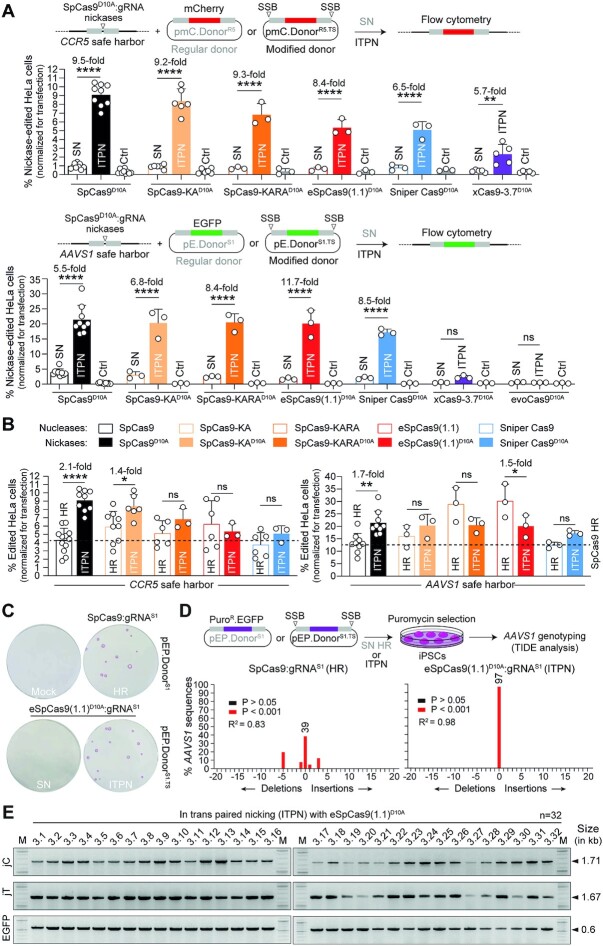
Testing SSB-dependent genome editing using regular versus high-specificity SpCas9^D10A^ nickases. (**A**) Single nicking and in trans paired nicking genome editing based on high-specificity SpCas9^D10A^ variants. Nickase-dependent genome editing frequencies in HeLa cells transfected with the depicted components targeting *CCR5* and *AAVS1* were measured by reporter-directed flow cytometry at 17 days post-transfection (top and bottom graphs, respectively). HeLa cells treated with corresponding Cas9^D10A^ nickases and regular donor plasmids in the absence of locus-specific gRNAs served as negative controls. Results are plotted as mean ± SD of at least three independent biological replicates. Significant differences between the indicated datasets were assessed by two-way ANOVA followed by Šidák's multiple comparisons test; ****P < 0.0001, **0.001 < *P*< 0.01; *P*> 0.05 was considered non-significant (ns). (**B**) Comparing standard and in trans paired nicking genome editing strategies at *CCR5* and *AAVS1*. Plotting of datasets presented in panel A corresponding to HeLa cells subjected to nucleases and regular donors or to nickases and target site-modified donors (canonical HR or ITPN strategies, respectively). Dashed lines mark the means of the DSB-dependent genome editing levels obtained with conventional SpCas9 and unmodified HR donor templates. Data are shown as mean ± SD of at least 3 independent biological replicates. Significant differences between the indicated datasets were calculated by two-way ANOVA followed by Šidák's multiple comparisons tests; ****P < 0.0001, **0.001 < *P*< 0.01, *0.01 < *P*< 0.05; *P*> 0.05 was considered non-significant (ns). (**C**) Testing standard and in trans paired nicking in iPSCs using high-specificity cleaving and nicking CRISPR complexes. iPSCs edited upon exposure to the indicated *AAVS1*-targeting reagents were selected in the presence of puromycin and the resulting colonies were stained for the pluripotency marker alkaline phosphatase. (**D**) Probing mutagenic loads in genome-edited iPSCs. iPSCs edited after exposure to the indicated *AAVS1*-targeting reagents were selected in the presence of puromycin and indel profiles at *AAVS1* were examined through tracking of indels by decomposition (TIDE) analysis. (**E**) Establishing HDR-mediated transgene insertion in iPSCs edited through in trans paired nicking. Junction PCR analysis was performed on randomly picked iPSC clones engineered through pEP.Donor^S1.TS^ and eSpCas9(1.1)^D10A^:gRNA^S1^ delivery.

Previous research from our laboratory using DNA/gRNA mismatch screens demonstrated that the specificities of mutant SpCas9^D10A^ variants exceeds by manifold that of the parental SpCas9^D10A^ nickase (44). Here, to further improve the seamless and scarless character of ITPN genome editing, we sought to investigate its compatibility with these high-specificity nickases, namely, SpCas9-KA^D10A^, SpCas9-KARA^D10A^, eSpCas9(1.1)^D10A^, Sniper-Cas9^D10A^, xCas9-3.7^D10A^, evoCas9^D10A^ and SpCas9-HF1^D10A^ (Supplementary Figure S5). To this end, we started by comparing the performances of parental SpCas9^D10A^:gRNA complexes with those of high-specificity SpCas9^D10A^ variants using regular and target site-modified donors for single nicking (SN)- and ITPN-mediated HR, respectively. Thus, HeLa cells were transfected with unmodified or target site-modified donors together with isogenic constructs expressing specific nickases and canonical gRNAs targeting *CCR5* and *AAVS1* acceptor sequences (Figure 3A, top and bottom graphs, respectively). After a 2-week sub-culturing period, SSB-dependent genome editing frequencies were assessed by reporter-directed flow cytometry. These experiments revealed that, at *CCR5*, the best-performing nickase was SpCas9-KA^D10A^ (Figure 3A, top graph) whilst at *AAVS1*, SpCas9-KA^D10A^ together with SpCas9-KARA^D10A^ and eSpCas9(1.1)^D10A^ induced ITPN genome editing to the same extent as the parental SpCas9^D10A^ nickase (Figure 3A, bottom graph). Consistent with the nuclease screens (Figure 1), xCas9-3.7^D10A^ and evoCas9^D10A^ triggered the lowest frequencies of SSB-dependent genome editing (Figure 3A). Additional experiments showed that both SpCas9^D10A^ and eSpCas9(1.1)^D10A^ outperformed SpCas9-HF1^D10A^ at *AAVS1* and *CLYBL*, with the highest ITPN genome editing levels induced by these nickases registered at the former locus (Supplementary Figure S6).

Significantly, the comparison of precise HR setups encompassing ITPN and genomic DSBs (canonical HR), revealed that, except when directing eSpCas(1.1)^D10A^ to *AAVS1*, ITPN reached similar or significantly higher frequencies of genome-edited cells than canonical HR (Figure 3B). Complementing *AAVS1* gene targeting experiments in iPSCs using SpCas9 and eSpCas9(1.1)^D10A^, besides confirming the poor performance of SN genome editing (Figure 3C), further corroborated that ITPN mostly avoids target allele disruptions (Figure 3D) while achieving precise HR-derived chromosomal insertions (Figure 3E).

**Figure 4.**
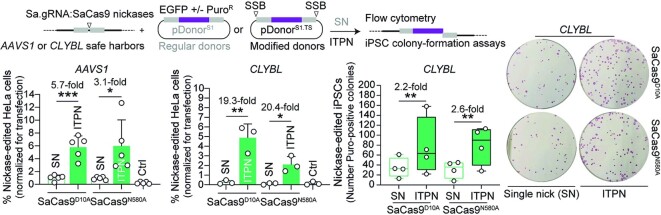
Genome editing combining regular SN plasmid donors or modified ITPN donors and nicking orthogonal SaCas9 complexes. SaCas9^D10A^- or SaCas9^N580A^-dependent genome editing at *AAVS1* and *CLYBL* loci in HeLa cells using EGFP-encoding donors, and at these loci in iPSCs using Puro^R^.EGFP-encoding donors, was assessed by reporter-directed flow cytometry and colony-formation assays, respectively. The latter assay detected the pluripotency marker alkaline phosphatase to identify puromycin-resistant iPSCs. Controls consisted of cells exposed to regular donor plasmids and nickases lacking locus-specific gRNAs. Data are shown as mean ± SD of at least three independent biological replicates. Significant differences between the indicated datasets were calculated by two-tailed unpaired Student's *t* tests (left and middle graphs) and two-tailed paired ratio *t* test (right graph); ***0.0001 < *P*< 0.001, **0.001 < *P*< 0.01, *0.01 < *P*< 0.05.

Towards broadening the scope of ITPN genome editing, we next performed experiments in HeLa cells and iPSCs using *AAVS1*- and *CLYBL*-targeting donors together with orthologue SaCas9^D10A^ and SaCas9^N580A^ nickases (Figure 4). SSB-dependent genome editing frequencies were assessed by flow cytometry of EGFP-expressing HeLa cells or by iPSC colony-formation assays (Figure 4). Consistent with the experiments using parental SpCas9^D10A^ and high-specificity SpCas9^D10A^ derivatives (Figure 3A), the HR setups involving ITPN were more effective than those entailing SN (Figure 4). However, in contrast to the experiments using SpCas9^D10A^ nickases (Figure 3A), neither SaCas9^D10A^ nor SaCas9^N580A^ led to genome editing frequencies higher than those obtained through SaCas9-induced canonical HR (Figure 4 and Supplementary Figure S7). These data indicate that when compared to *S. aureus* SaCas9^D10A^ and SaCas9^N580A^ nickases, *S. pyogenes* SpCas9^D10A^ nickases are preferable for ITPN genome editing, especially so in their high-specificity configurations.

**Figure 5.**
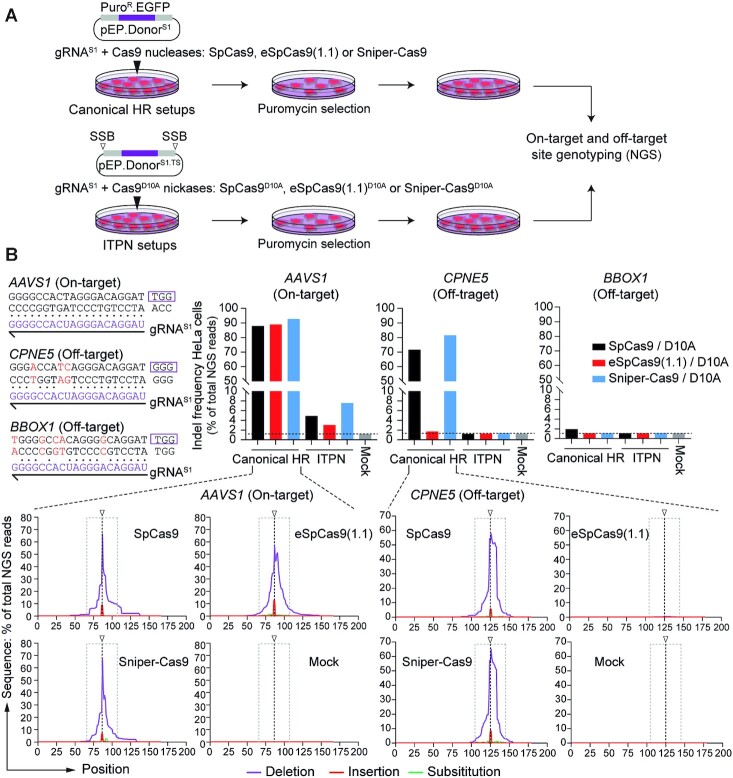
Assessing mutagenic loads in cells edited through canonical homologous recombination versus in trans paired nicking. (**A**) Experimental design. HeLa cells were exposed to regular and modified donors conferring puromycin resistance together with SpCas9 nucleases and SpCas9^D10A^ nickases, respectively. SpCas9, eSpCas9(1.1) and Sniper-Cas9 nucleases, and their D10A nickase derivatives, were coupled to *AAVS1*-targeting gRNA^S1^. Indel frequencies at on-target and off-target sites was done by amplicon deep sequencing genotyping of puromycin-resistant cell populations. (**B**) Quantification of indels at on-target and off-target sites. CRISPR complex-derived indels at the *AAVS1* target site and at two validated off-target sites (i.e. *CPNE5* and *BBOX1*) were quantified by amplicon deep sequencing (∼100,000 paired-end reads per sample). Nucleotide mismatch positions between gRNA^S1^ spacer and off-target *CPNE5* and *BBOX1* sequences are highlighted in red. The types and distributions of indels detected within *AAVS1*, *CPNE5* and *BBOX1* in cells treated with regular and high-specificity nucleases are plotted. HeLa cells not exposed to CRISPR complexes provided for negative controls (Mock).

Orthogonal high-throughput genome-wide translocation sequencing (oHTGTS) permits tracing off-target effects of CRISPR nucleases vis-à-vis nickases in a quantitative and unbiased fashion (28,44). In our earlier work, oHTGTS assays showed a striking and progressive reduction of off-target activities associated with SpCas9, high-specificity eSpCas9(1.1) and SpCas9^D10A^. A more moderate, yet readily measurable, further reduction in off-target effects was detected when using the high-specificity eSpCas9(1.1)^D10A^ nickase instead of its parental SpCas9^D10A^ counterpart (44). Moreover, oHTGTS assays also disclosed sequences mapping at *CPNE5* and *BBOX1* as the top-ranked off-target sites for CRISPR complexes formed by coupling the *AAVS1*-specific gRNA^S1^ to SpCas9 and SpCas9^D10A^, respectively (28). Therefore, we proceeded by assessing the integrity of *AAVS1*, *CPNE5* and *BBOX1* in HeLa cell populations genome-edited through canonical HR using SpCas9:gRNA^S1^, eSpCas9(1.1):gRNA^S1^ or Sniper-Cas9:gRNA^S1^ or via ITPN using their corresponding D10A nickase derivatives (Figure 5A). Targeted amplicon deep sequencing confirmed high and low indel frequencies at *AAVS1* in cells exposed to nuclease and nickase complexes, respectively (Figure 5B). Furthermore, in striking contrast to eSpCas9(1.1), the regular SpCas9 and high-specificity Sniper-Cas9 nucleases led to similar and high frequencies of indels at the *CPNE5* off-target site. Significantly, none of the nickase complexes tested induced detectable off-target activities using the sensitive deep sequencing genotyping assays (Figure 5B).

**Figure 6.**
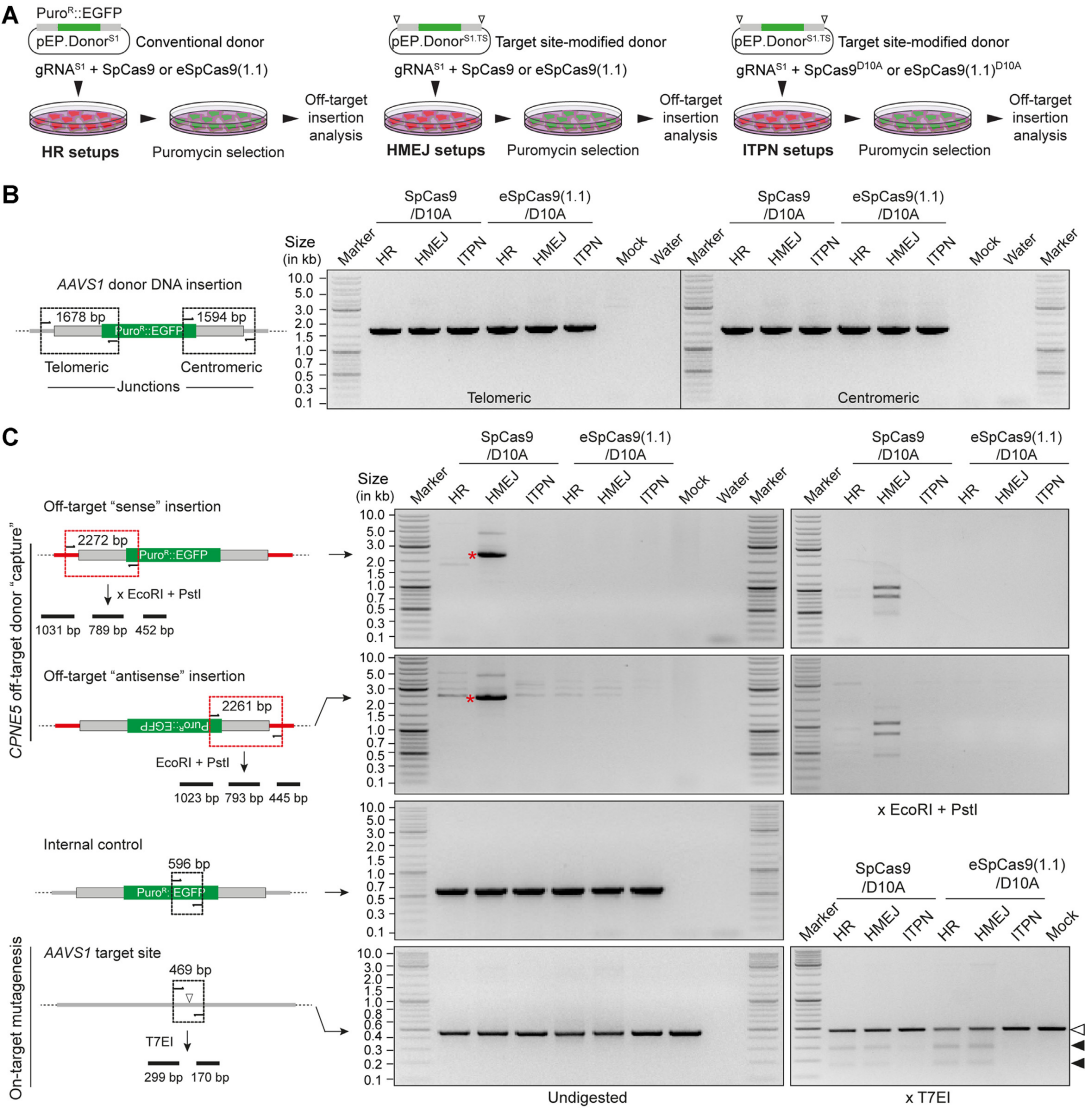
Assessing off-target chromosomal donor DNA insertions resulting from HR, HMEJ and ITPN using regular and high-specificity Cas9 enzymes. (**A**) Experimental design. HeLa cells were subjected to HR, HMEJ and ITPN procedures using the indicated combinations of donor DNA constructs and Cas9 proteins coupled to *AAVS1*-targeting gRNA^S1^. Genetically modified cells, selected through puromycin exposure, were screened for donor DNA ‘capture’ at the prevalent gRNA^S1^ off-target site *CPNE5* by junction PCR analysis. (**B**) On-target donor DNA insertion analysis. Amplicons diagnostics for HDR-mediated *AAVS1* knock-ins are illustrated and shown. (**C**) Off-target insertion and on-target mutagenesis analysis. Amplicons diagnostics for HDR-independent ‘capture’ of donor DNA sequences at *CPNE5* in the ‘sense’ and ‘antisense’ orientations are illustrated and marked with asterisks. Specific donor DNA ‘capture’ at *CPNE5* off-target alleles and mutagenesis at *AAVS1* target alleles were probed via restriction enzyme (EcoRI and PstI) and T7 endonuclease I (T7EI) digestions, respectively. Solid arrowheads point to T7EI-digested products derived from indel-containing *AAVS1* sequences. PCR amplifications of a 596-bp *EGFP* tract served as internal controls.

As aforementioned, ‘double-cut’ donors susceptible to HMEJ, MMEJ and NHEJ are normally more efficient genome editing substrates than their HR counterparts. Yet, the free termini generated *in cellula* from ‘double-cut’ donors upon site-specific DNA cleavage might diminish the genome editing precision due to end-to-end ligation (‘capture’) at off-target DSBs (24,56). Thus, to further investigate genome editing precision using conventional and high-specificity Cas9 proteins, HeLa cells were genetically modified through HR, HMEJ and ITPN (Figure 6A), and then analysed for on-target and off-target donor DNA insertion at *AAVS1* and *CPNE5* (Figure 6B and C, respectively). Besides confirming donor DNA targeting at *AAVS1* (Figure 6B), junction PCR analysis established that HMEJ donors are the most prone to HR-independent ‘capture’ at off-target sequences and that these unwanted outcomes can be minimized by using high-specificity instead of parental SpCas9 nucleases (Figure 6C). Finally, genotyping assays based on T7 endonuclease I (T7EI) digestions for indel detection (Figure 6C) and DNA sequencing (Supplementary Figure S8) strengthened the value of ITPN for precise chromosomal insertion of large genetic payloads with minimal bystander effects at target alleles within genome-edited cell populations.

Collectively, these experiments have identified Cas9 nickases whose combined high specificities and activities turns them into valuable alternatives to the regular SpCas9^D10A^ nickase for use in ITPN genome editing settings and stress the relevance of using high-specificity SpCas9 nucleases, especially when aiming at targeted insertion of free-ended donor DNA.

### CRISPR-Cas9 nickases fail to activate the P53-dependent DNA damage response in iPSCs

Single to few DSBs suffice to induce P53 signalling in stem cells (29,30) causing cell cycle arrest at G1. Hence, CRISPR-Cas9-induced HR is hindered in cells with functional P53 as it takes place during the S through G2 phases of the cell cycle (25). Indeed, P53 absence or inhibition correlates with enhanced DSB-dependent genome editing (29–31,57).

**Figure 7.**
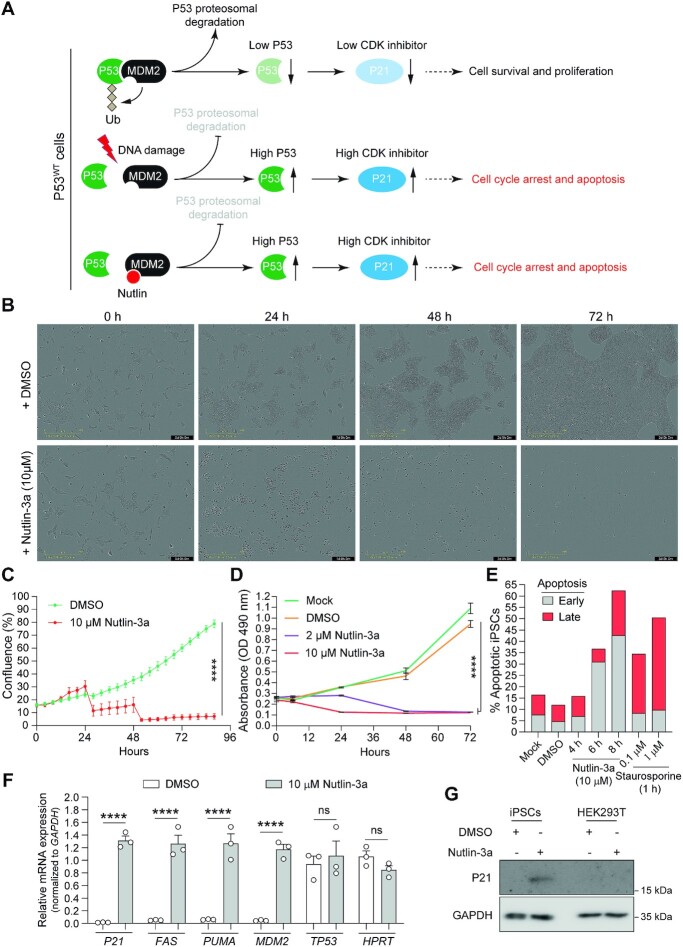
Cell survival assay for assessing P53 functionality in human iPSCs. (**A**) Schematics of post-transcriptional P53 activity control by DNA damage and Nutlins. In cells with normal amounts of P53, DNA damage activates ATM/ATR kinases that disrupt P53-MDM2 interaction through P53 phosphorylation. Free P53 escapes proteasomal degradation and upregulates the expression of downstream target genes (e.g. cyclin-dependent kinase inhibitor P21) inducing cell cycle arrest and apoptosis. Nutlins disrupt the P53-MDM2 interaction by instead occupying the P53 binding pocket in MDM2 mimicking a P53-dependent DNA damage response. Conversely, in cells with no or low amounts of P53, nutlins induce neither cell cycle arrest nor apoptosis (not drawn). (**B** and **C**) Realtime cell proliferation assay. The proliferation of human iPSCs incubated in the presence of Nutlin-3a (10 μM) or vehicle (DMSO) was quantified in a live-cell imaging system (IncuCyte) for 3 days. Data are shown as mean ± SD of 6 technical replicates. Significant differences between the indicated datasets were calculated by two-way ANOVA tests; ****P < 0.0001. (**D** and **E**) Cell survival assays. The survival of human iPSCs incubated in regular medium (Mock) or in medium supplemented with DMSO or Nutlin-3a (2 μM and 10 μM) was monitor for 3 days by using the MTS cell metabolic activity readout (panel D). The frequencies of apoptotic human iPSCs were determined with a combined annexin V/propidium iodide assay (panel E). Annexin V positive cells and annexin V/propidium iodine doubly positive cells measured by flow cytometry scored for early and late apoptosis, respectively. Prior to flow cytometry the cells were incubated in regular medium (Mock) and in medium supplemented with DMSO or with Nutlin-3a (10 μM) for different periods. Staurosporine applied at the indicated conditions served as an apoptosis-inducing control. (**F**) Assessing P53-dependent responses in human iPSCs exposed to Nutlin-3a. RT-qPCR analysis of transcripts for P53 and P53-responsive genes were conducted in human iPSCs incubated for 5 h in regular medium or in medium supplemented with Nutlin-3a (10 μM). RT-qPCR analysis of *HPRT1* transcripts served to measure the expression of a P53-independent control gene (*n* = 3 independent biological replicates). Significances were calculated with two-way ANOVA followed by Šidák's test for multiple comparisons; ****P < 0.0001; *P*> 0.05 was considered non-significant (ns). (**G**) P53-dependent P21 protein detection assay. Western blot analysis of P21 expression in human iPSCs incubated in the presence of Nutlin-3a (10 μM) or vehicle (DMSO) for 12 h. Transformed P53-defective HEK293T cells exposed to the same experimental conditions served as control. Western blotting of the housekeeping GAPDH provided for loading controls.

A recent study showed that SpCas9^D10A^ did not significantly activate P53 signalling in cervical carcinoma and mammary epithelial cell lines, i.e. HeLa and MCF10A cells, respectively (37). To examine P53 signalling elicited by nickases versus nucleases in cells with a low sensitivity threshold to DNA damage, we selected human iPSCs owing to their established relevance in basic and translational research. Besides present in over 50% of cancers, cells with P53 mutations can recurrently arise in cultures of pluripotent stem cells (PSCs) (58). Importantly, real-time cell proliferation assays in the presence and absence of Nutlin-3a, a small-molecule inhibitor of P53-MDM2 interactions (Figure 7A), demonstrated that the selected iPSCs have a functional P53 status (Figure 7B and C; Supplementary files S1 and S2). This conclusion was independently confirmed by measuring cell viability using metabolic and apoptosis activity assays (Figure 7D and E, respectively) and by detecting the specific upregulation of the P53 target genes *FAS*, *PUMA* and *MDM2* at the transcriptional level and of *P21* at the transcriptional and protein levels (Figure 7F and G, respectively).

**Figure 8.**
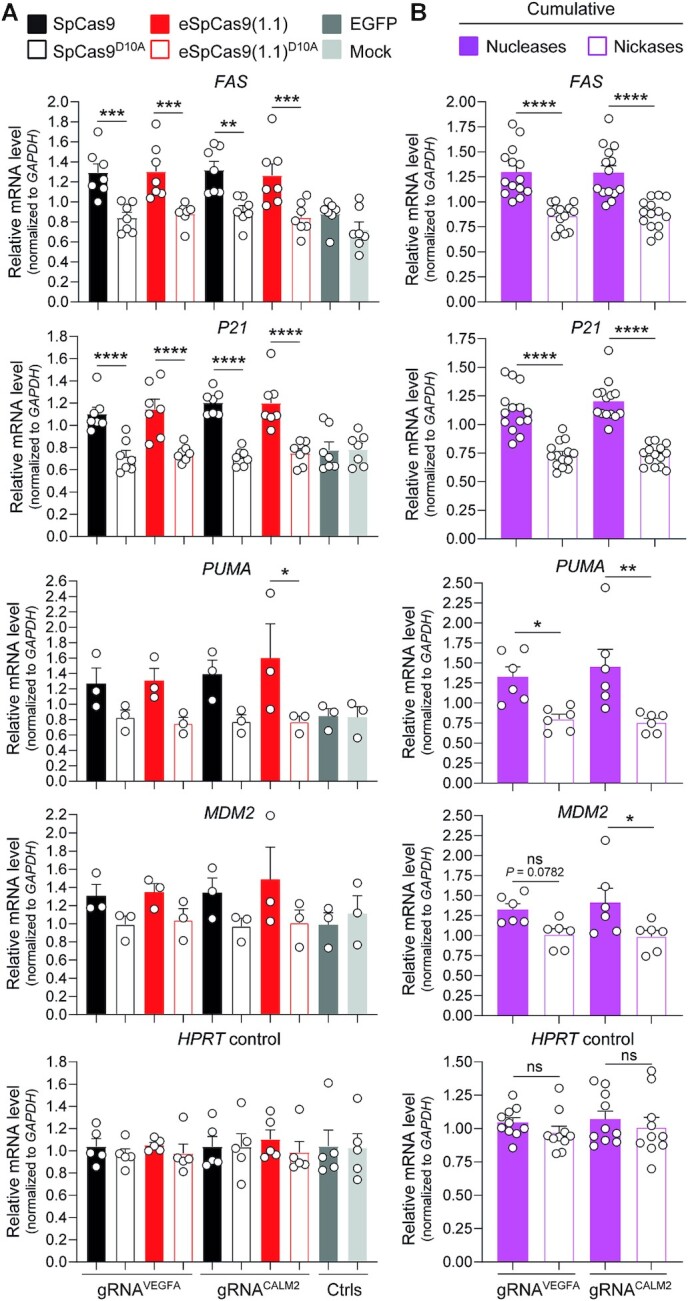
Assessing activation of P53-dependent DNA damage responses in human iPSCs exposed to nucleases versus nickases. (**A**) Expression analysis of P53 activation-responsive genes. Constructs encoding the indicated Cas9 enzymes and gRNAs conferring high (gRNA^VEGFA^) or low (gRNA^CALM2^) off-target activities (Supplementary Figure S9), were transfected into iPSCs. RT-qPCR measurements of *FAS*, *P21*, *PUMA* and *MDM2* transcripts whose expression is upregulated upon P53 activation (minimum *n* = 3 independent biological replicates). Targeting *HPRT1* transcripts served for RT-qPCR measurements of a housekeeping control gene (n = 5 independent biological replicates). Additional controls consisted of targeting *FAS*, *P21*, *PUMA*, *MDM2* and *HPRT1* transcripts in mock-transfected iPSCs and in iPSCs transfected with an EGFP-encoding plasmid. Significances were calculated with one-way ANOVA followed by Tukey's test for multiple comparisons; ****P < 0.0001, ***0.0001 < *P*< 0.001, **0.001 < *P*< 0.01, *0.01 < *P*< 0.05. (**B**) Cumulative comparison of cleaving versus nicking effects on P53-responsive gene modulation. Combined RT-qPCR datasets derived from iPSCs treated with nucleases SpCas9 and eSpCas9(1.1) or nickases SpCas9^D10A^ and eSpCas9(1.1)^D10A^. Significances were calculated with two-way ANOVA followed by Šidák's test for multiple comparisons; ****P < 0.0001, **0.001 < *P*< 0.01, *0.01 < *P*< 0.05; *P*> 0.05 was considered non-significant (ns).

Next, the iPSCs were transfected with constructs expressing regular or high-specificity SpCas9 proteins and gRNA^CALM2^ or gRNA^VEGFA^. The former and latter gRNAs are known to have few and numerous off-target sites, respectively, as assessed *in silico* (Supplementary Figure S9) and experimentally (8,29,45). Expression analysis of the P53 transcription factor target genes *FAS* and *P21* disclosed that coupling SpCas9 and high-specificity eSpCas9(1.1) nucleases to the promiscuous gRNA^VEGFA^ led to significant activation of P53 signaling, whilst coupling the same gRNA^VEGFA^ to SpCas9^D10A^ and eSpCas9(1.1)^D10A^ nickases, did not (Figure 8A). Moreover, high-specificity gRNA^CALM2^ also led to nuclease-dependent upregulation of *FAS* and *P21* expression (Figure 8A). Cumulative datasets comparing nuclease- versus nickase-mediated activation of the P53-responsive genes *FAS*, *P21*, *PUMA* and *MDM2* revelated that SpCas9^D10A^ nickases are poor triggers of the P53-dependent DDR in iPSCs when compared to SpCas9 nucleases (Figure 8B).

Together, these results indicate that genome editing with SpCas9^D10A^ nickases might offer a heightened safety profile to engineered cell products derived from iPSCs in that, besides cell-cycle arrest and apoptosis, DSB-induced signalling pathways have been linked to the selection of cells with mutations in cancer-associated genes, i.e. *TP53* and *KRAS* (31,32).

### In contrast to genome editing based on high-specificity Cas9 nucleases, ITPN facilitates editing essential and non-unique allelic sequences in iPSCs

**Figure 9.**
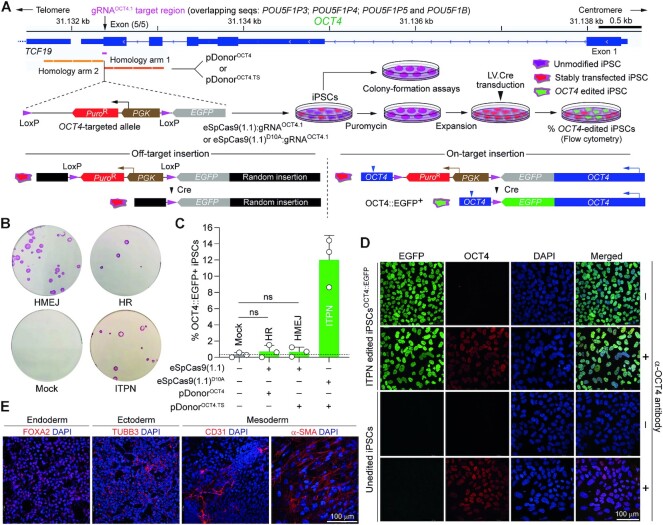
Testing DSB- versus SSB-dependent genome editing strategies at essential *OCT4* alleles in human iPSCs using high-specificity CRISPR complexes. (**A**) Experimental setup for tracking *OCT4* gene editing events. iPSCs exposed to the indicated reagents designed to elicit canonical HR, HMEJ or ITPN were traced by colony-formation assays upon puromycin selection and alkaline phosphatase staining and by a genetic assay reporting live-cell *OCT4* gene targeting events upon Cre recombinase delivery. (**B**) Detection of stably transfected iPSC colonies. Picture of a representative colony-formation assay is shown. (**C**) Detection of *OCT4* gene editing events. The frequencies of *OCT4* edited cells (OCT4::EGFP^+^) in puromycin-resistant iPSC populations were determined by EGFP-directed flow cytometry following transduction with Cre-expressing lentivector particles (20 vector particles per cell). Data are presented as mean ± S.D. of independent biological replicates (*n* = 3). (**D**) Confocal microscopy analysis of iPSCs edited at *OCT4* through ITPN. OCT4::EGFP-expressing iPSCs engineered through ITPN and Cre delivery (iPSC^OCT4::EGFP^) were analysed through immunofluorescence microscopy for detecting OCT4 and EGFP, respectively. Nuclei were stained with DAPI. The merge of the three fluorescence signals highlights the nuclear localization of the OCT4::EGFP fusion product. Unedited iPSCs served as negative controls. iPSC and iPSC^OCT4::EGFP^ specimens not incubated with the OCT4-specific primary antibody served as staining controls. (**E**) Assessing the multi-lineage differentiation capacity of iPSCs edited at *OCT4* through ITPN. iPSCs^OCT4::EGFP^ generated by ITPN using high-specificity eSpCas9(1.1)^D10A^ were induced to differentiate into cell lineages corresponding to the three embryonic germ layers, i.e. mesoderm, ectoderm and endoderm. Immunofluorescence microscopy detected the indicated embryonic germ layer-specific markers. Nuclei were stained with DAPI.

Programmable nucleases can elicit cell fitness losses and unpredictable phenotypes upon cutting DNA sequences coding for essential proteins or motifs or that are recurrent in the genome (28,59,60). *OCT4* (alias *POU5F1*) encodes a transcription factor essential for human embryogenesis (61) and PSC maintenance (62,63). The essentiality of *OCT4* combined with its extensive homology to pseudogenes *POU5F1B*, *POU5F1P3*, *POU5F1P4* and *POU5F1P5* makes its editing particularly challenging. Indeed, at both coding termini, *OCT4* shares 100% homology to pseudogene sequences making it impossible designing gRNAs specific for these regions or for tagging OCT4 (Supplementary Figure S10). Hence, retrieving PSCs edited at such multiple-copy sequences is expected to be hindered by the acute sensitivity of these cells to DSBs. Three lines of evidence support this assertion. Firstly, OCT4 tagging experiments in iPSCs using TALENs and donor construct pDonor^OCT4^ (Figure 9A), did not yield HR-targeted clones (0/48) (64). Secondly, experiments in human embryonic stem cells deploying SpCas9 and donor templates with the same ‘homology arms’ present in pDonor^OCT4^, led only to eight HR-targeted clones (8/288) (65). Finally, our earlier experiments in iPSCs showed that, in contrast to pDonor^OCT4^ and SpCas9:gRNA^OCT4.1^ delivery (HR setup), transfer of modified pDonor^OCT4.TS^ and nicking SpCas9^D10A^:gRNA^OCT4.1^ complexes (ITPN setup), readily led to OCT4-tagged iPSC populations from which viable HR-targeted iPSC clones were obtained (21/22) (28).

In this study, complementing experiments using the same live-cell gene editing readout and high-specificity DNA cleaving and nicking CRISPR complexes revealed that although canonical HR, HMEJ and ITPN setups all led to stably transfected iPSCs (Figure 9B), only the latter setup resulted in accurate *OCT4* editing at frequencies significantly above background levels (Figure 9C). These results demonstrate that despite high-specificity Cas9 nuclease usage, generating viable OCT4-tagged iPSCs is nonetheless hindered when applying the DSB-dependent genome editing strategies. Importantly, this is not the case when employing the ITPN approach instead. Moreover, dual-colour confocal microscopy analysis established that iPSCs edited through eSpCas9(1.1)^D10A^-induced ITPN contained engineered OCT4::EGFP fusion proteins properly localized in cell nuclei (Figure 9D). Finally, the *OCT4* edited cells were capable of differentiating into cells representing the three embryonic germ layers, i.e. endoderm, mesoderm and ectoderm (Figure 9E).

Collectively, these results support the proposition that, irrespective of their specificities, programmable nucleases are outperformed by nickases for targeted and high-fidelity DNA knock-ins at sequences associated with essentiality and recurrence in the genome.

## DISCUSSION

In this work, we have identified high-specificity SpCas9 nucleases that once combined with donor constructs tailored for HR or HMEJ can, in a locus-dependent manner, trigger genome editing to similar or higher levels than those elicited by the parental SpCas9 nuclease, i.e. SpCas9-KA (8), SpCas9-KARA (8), eSpCas9(1.1) (8), Sniper-Cas9 (11). These results contrast with those obtained with xCas9-3.7 (10), evoCas9 (9) and SpCas9-HF1 (7) in that these high-specificity nucleases normally yield the lowest frequencies of genome-edited cells. Potentially, the modulation of DNA binding, catalytic checkpoint thresholds (4–6) and/or post-cleavage residence times (66) by different sets of SpCas9 mutations controls target-donor engagement and ultimate gene knock-in proficiencies. It is equally possible that specific chromatin contexts have a bearing on gene knock-ins involving different SpCas9 variants (67,68). Notwithstanding the individual mechanisms or combinations thereof, the genome-editing levels reached by delivering HDR-tailored donor constructs together with different SpCas9 variants largely correlate with the DNA cleaving activities of the latter tools as scored through gene knockout assays (44).

Although the mechanisms underlying recombination between target and HMEJ donors have not been dissected, it is sensible to postulate the participation of canonical HR and MMEJ factors in that HMEJ donors, similarly to HR and MMEJ donors, have long homology tracts and are substrates to DNA end-processing, respectively. Regardless, consistent with earlier investigations using parental SpCas9 (22–24), HMEJ donors were the most proficient gene knock-in substrates once combined with the above-mentioned high-efficiency SpCas9 nucleases, independently of cell type or endogenous locus targeted.

Clearly, off-target chromosomal DSBs are undesirable in that these lesions are *bona fide* substrates for NHEJ processes and, as such, they are prone to mutations and to donor DNA ‘capture’ at unintended genomic locations. The latter by-products arise most frequently when free-ended linear DNA substrates are presented in cell nuclei, such as those resulting from ‘double-cut’ donors (56). In fact, the ‘capture’ of free-ended double-stranded DNA at chromosomal DSBs forms the basis of pipelines for genome-wide detection of programable nuclease off-target activities (69,70). Moreover, in addition to reducing genome-editing fidelity, off-target exogenous DNA insertions heighten cellular transformation risks. Further to this point, the emergence of severe adverse events in gene therapy clinical trials using retroviral vectors harbouring strong viral enhancers offers a cautionary example of such insertional oncogenesis phenomena (71). Importantly, we have demonstrated that off-target ‘capture’ of exogenous DNA resulting from the processing of HMEJ donors are minimized via using high-specificity instead of parental SpCas9 nucleases. Hence, the high-specificity SpCas9 nucleases identified here as efficient tools for DSB-dependent genome editing are expected to be particularly suited for gene knock-ins entailing HMEJ and, possibly, other types of ‘double-cut’ donors, such as those prone to NHEJ or MMEJ.

Genetic and pharmacological approaches that, by modulating DSB repair pathway choice, favour precise HDR-mediated genome editing, are under intense investigation (72). High-specificity SpCas9 nucleases were recently shown to have potential in this regard. Specifically, systematic experiments using double-stranded oligonucleotide donors revealed that high-specificity SpCas9 variants can, in a target site-dependent manner, bias DSB repair towards HDR at the expense of non-homologous end-joining (73). In most instances, however, HDR events remain underrepresented. Contrary to DSBs, nicks are non-canonical substrates for mutagenic DNA end-joining processes. By recruiting SSB-dependent HR pathways, ITPN genome editing strategies (24,35,37,41), generically based on tandem nicking of donor and target DNA by SpCas9 nickases (4), introduce a low mutagenic burden in edited cell populations. As a result, these approaches are particularly fitting for minimizing haploinsufficiency (28), for clonal screening-free generation of model cells and organoids as well as for biallelic, multiplexing and allele-specific gene editing (24,39–41). In this study, we have identified high-specificity SpCas9^D10A^ nickases capable of eliciting ITPN genome editing to the same extent as that triggered by the parental SpCas9^D10A^ protein. Significantly, at the *CCR5* and *AAVS1* safe harbours, ITPN setups comprising members from this nickase panel (i.e. SpCas9-KA^D10A^, SpCas9-KARA^D10A^, eSpCas9(1.1)^D10A^ and Sniper-Cas9^D10A^) outperformed the reference HR setup involving regular donor constructs and the SpCas9 nuclease. Importantly, indel ‘footprints’ installed at target and off-target sequences in genome-edited cell populations by high-specificity SpCas9^D10A^ nickases were rare and undetected, respectively. In contrast, cell populations edited through regular and high-specificity SpCas9 nucleases had over 80% of their target alleles disrupted as quantified by amplicon deep sequencing. This data underscores the high and low mutagenic burdens imposed on cells subjected to SpCas9 nucleases and nickases, respectively.

Improving the efficiency and precision of stem cell engineering is in demand owing to the increasing role that these technologies are having in science and medicine. P53-dependent cytostatic and cytotoxic responses triggered by DSBs (targeted or otherwise) limits the efficacy of genome editing in stem cells, e.g. PSCs and hematopoietic stem cells (HSCs) (29,30). To assess P53 signaling in cells with high sensitivity to DNA damage, we exposed human iPSCs to regular and high-specificity SpCas9 nucleases, or to their respective D10A nickase counterparts, along with specific or promiscuous gRNAs. We found that in contrast to SpCas9 nucleases, neither regular nor high-specificity SpCas9^D10A^ nickases significantly activate the canonical P53 signalling pathway. As a corollary, cell therapy products derived from human iPSCs engineered with high-specificity Cas9 nickases might offer a heighten safety profile over those made through nuclease exposure. Indeed, DSB-mediated activation of signalling pathways has been shown to select for cells with potentially harmful loss-of-function or dominant-negative mutations in the tumor-suppressor P53 transcription factor or gain-of-function mutations in the K-RAS oncoprotein (31,32). Further to this point, PSCs are capable of ‘spontaneously’ acquiring cancer associated P53 mutations in a recurrent fashion (58). Therefore, these cells are more resistant to DSBs and, as a result, more prone to expansion than their wild-type counterparts once exposed to programmable nucleases. Moreover, recent mouse model data support the conclusion that p53 mutant cells, rather than progressing to full malignancy in a strictly haphazard fashion, suffer instead a more deterministic series of genetic instability events (74).

ITPN genome editing permits accessing in a seamless fashion challenging genomic sequences in the form of target DNA sharing high homology to off-target sites and/or coding for essential cellular functions (28). By targeting the pluripotency supporting *OCT4* gene as such a genomic locus, we provide evidence for the utility of high-specificity nicking CRISPR complexes over their DNA cleaving counterparts for achieving gene knock-ins at essential and non-unique allelic sequences in iPSCs. In this context, ITPN and complementary DSB-free technologies, such as those based on prime editors, should widen the options for precise genome editing at challenging (or otherwise) genomic sequences (75). Prime editors consist of Cas9 nickases fused to engineered reverse transcriptases and extended prime editing (PE) gRNAs (pegRNAs) that simultaneously define target and editing sequences. In contrast to ITPN and other HDR-based strategies, PE does not require delivery of donor DNA templates and allows for efficient DNA insertions of up to ∼44 bp even if substantial pegRNA optimization is typically necessary (75,76). Moreover, work from our laboratory and that of others has recently disclosed that PE is more limited in non-cycling than in cycling cells (77,78). Yet, differently from HDR-based genome editing, it can perform in post-mitotic cells *in vitro* and *in vivo* (75,77). Recent developments on PE technologies that comprise the use of dual pegRNAs and site-specific recombinases permit replacing target sequences with up 250-bp of foreign DNA and inserting whole transgenes at a prime editor-placed recombination site, respectively (75). These combinatorial approaches are powerful and versatile despite requiring the delivery of large and multicomponent reagents into target cells. Moreover, PE based on dual pegRNAs is not amenable to large DNA insertions whilst, when compared to conservative HR-based ITPN, combinatorial PE and site-specific recombination is less amenable to subtle genomic edits, such as those involving endogenous gene repair, due to ‘footprint’ installation in the form of recombinase target sites.

In conclusion, genome editing based on high-specificity CRISPR-Cas9 complexes and donor DNA constructs prone to defined HDR processes (i.e. HR, HMEJ or ITPN) constitute a complementary set of precision genetic engineering strategies with enhanced performances and heightened safety profiles. Indeed, the HR, HMEJ and ITPN genome editing strategies investigated here can be selected based on specific experimental or biotechnological contexts and associated goals. Namely, HMEJ as the most robust strategy across different genomic target sites (Supplementary Figure S11) and ITPN as the least mutagenic and cytotoxic should be particularly suited for applications profiting from high-efficiency and high-fidelity genome editing, respectively (Figure 6 and Supplementary Figure S12). Regarding the latter parameter, we found that SpCas9^D10A^ nickases are poor triggers of P53 signalling in human iPSCs, which makes them a fitting tool for the genomic engineering of cells with high sensitivity to DNA damage, e.g. pluripotent and tissue-specific stem cells.

## DATA AVAILABILITY

All data assembled for and analyzed in this study are included in the article and additional files. The libraries of next-generation sequencing reads are deposited at the NCBI Sequence Read Archive (SRA) database under BioProject ID PRJNA879334. The raw flow cytometry datasets are deposited in the FlowRepository under repository IDs: FR-FCM-Z5P9 (Detection of *OCT4* gene editing events), FR-FCM-Z5PA (Cleaving and nicking SaCas9 mediated gene editing), FR-FCM-Z5PB (Cleaving and nicking high-specificity Cas9 variants mediated gene editing). The donor DNA constructs designed for human safe harbor targeting through HR, HMEJ and ITPN and for expressing parental and high-specificity SpCas9^D10A^:gRNA complexes are available through the Addgene plasmid repository. AY27_pU6.gRNA.CLYBL (#199238); AM77_pU6.Sa-gRNA.CLYBL (#199237); AZ64_pE.DonorCLYBL.TS (#199228); AD59_pEP.DonorCLYBL.TS (#199227); BB44_pmc.DonorR5.TS (#199223); AP76_pU.CAG.Cas9-D10A-K848A.rBGpA (alias SpCas9-KA-D10A) (#199253); AP70_pU.CAG.Cas9-D10A-K848A-R1060A.rGBpA (alias SpCas0-KARA-D10A) (#199254); AA69_pU.CAG.Cas9-eSp(1.1)-D10A.rBGpA.2NLS (alias eSpCas9(1.1)-D10A) (#199252); BA31_pU.CAG.SaCas9-D10A.rBGpA (alias SaCas9-D10A) (#199251); AB65_pCAG.Cas9-D10A.rBGpA (alias SpCas9-D10A) (#199256) and AW01_pU.CAG.eSpCas9(1.1).rBGpA (#199255).

## Supplementary Material

gkad165_Supplemental_Files
