# Correction to ‘Precise homology-directed installation of large genomic edits in human cells with cleaving and nicking high-specificity Cas9 variants’

**DOI:** 10.1093/nar/gkag158

**Published:** 2026-02-23

This is a correction to: Qian Wang, Jin Liu, Josephine M Janssen, Manuel A F V Gonçalves, Precise homology-directed installation of large genomic edits in human cells with cleaving and nicking high-specificity Cas9 variants, Nucleic Acids Research, Volume 51, Issue 7, 24 April 2023, Pages 3465–3484, https://doi.org/10.1093/nar/gkad165

The authors have identified an error. The secondary anti-mouse antibody used was not donkey anti-mouse IgG Alexa Fluor 555 (Invitrogen; Cat. No. A31570), as listed in Supplementary Table S36, but goat anti-mouse IgG Alexa Fluor 568 (Invitrogen; Cat. No. A11004).

The error does not affect the results, discussion and conclusions presented in the article. These details have been corrected only in this correction notice to preserve the published version of record.

