# Supplementary material for: Precise homology-directed installation of large genomic edits in human cells with cleaving and nicking high-specificity Cas9 variants
Source: Nucleic Acids Res. 2023 Mar 17;51(7):3465–84. doi: 10.1093/nar/gkad165 (PMC10123109; doi:10.1093/nar/gkad165)
Supplement: gkad165_Supplemental_Files [file gkad165_supplemental_files.zip › Supplementary Information.pdf]

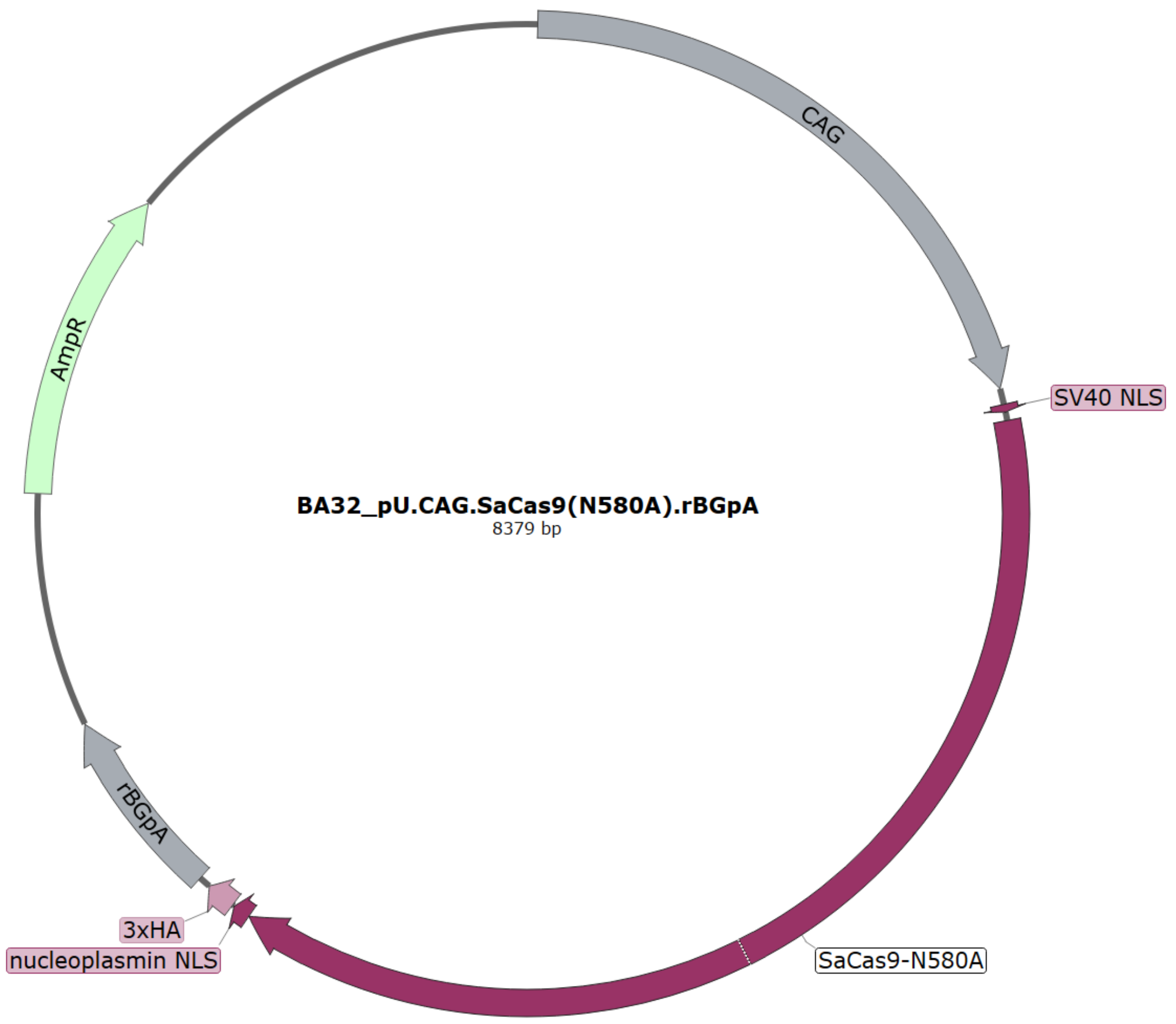


> BA32_pU.CAG.SaCas9$^{N580A}$(8379 bp)

GTTTAAACATTTAAATCTCGAGCCATGGATTCGACATTGATTATTGACTAGTTATTAATAGTAATCAA
TTACGGGGTCATTAGTTCATAGCCCATATATGGAGTTCCGCGTTACATAACTTACGGTAAATGGCCCG
CCTGGCTGACCGCCCAACGACCCCCGCCCATTGACGTCAATAATGACGTATGTTCCCATAGTAACGCC
AATAGGGACTTTCCATTGACGTCAATGGGTGGAGTATTTACGGTAAACTGCCCACTTGGCAGTACATC
AAGTGTATCATATGCCAAGTACGCCCCCTATTGACGTCAATGACGGTAAATGGCCCGCCTGGCATTAT
GCCCAGTACATGACCTTATGGGACTTTCCTACTTGGCAGTACATCTACGTATTAGTCATCGCTATTAC
CATGGTCGAGGTGAGCCCCACGTTCTGCTTCACTCTCCCCATCTCCCCCCCCTCCCCACCCCCAATTT
TGTATTTATTTATTTTTTAATTATTTTGTGCAGCGATGGGGGCGGGGGGGGGGGGGCGCGCGCCAGG
CGGGGCGGGGCGGGGCGAGGGGCGGGGCGGGGCGAGGCGGAGAGGTGCGGCGGCAGCCAATCAGAGCG
GCGCGCTCCGAAAGTTTCCTTTTATGGCGAGGCGGCGGCGGCGGCGGCCCTATAAAAAGCGAAGCGCG
CGGCGGGCGGGAGTCGCTGCGTTGCCTTCGCCCCGTGCCCCGCTCCGCGCCGCCTCGCGCCGCCCGCC
CCGGCTCTGACTGACCGCGTTACTCCCACAGGTGAGCGGGCGGGACGGCCCTTCTCCTCCGGGCTGTA
ATTAGCGCTTGGTTTAATGACGGCTCGTTTCTTTTCTGTGGCTGCGTGAAAGCCTTAAAGGGCTCCGG
GAGGGCCCTTTGTGCGGGGGGGAGCGGCTCGGGGGGTGCGTGCGTGTGTGTGTGCGTGGGGAGCGCCG
CGTGCGGCCCGCGCTGCCCGGCGGCTGTGAGCGCTGCGGGCGCGGCGCGGGGCTTTGTGCGCTCCGCG
TGTGCGCGAGGGGAGCGCGGCCGGGGGCGGTGCCCCGCGGTGCGGGGGGGCTGCGAGGGGAACAAAGG
CTGCGTGCGGGGTGTGTGCGTGGGGGGGTGAGCAGGGGGTGTGGGCGCGGCGGTCGGGCTGTAACCCC
CCCCTGCACCCCCCTCCCCGAGTTGCTGAGCACGGCCCGGCTTCGGGTGCGGGGCTCCGTGCGGGGCG
TGGCGCGGGGCTCGCCGTGCCGGGCGGGGGGTGGCGGCAGGTGGGGGTGCCGGGCGGGGCGGGGCCGC

CTCGGGCCGGGGAGGGCTCGGGGGAGGGGCGCGGCGGCCCCGGAGCGCCGGCGGCTGTCGAGGCGCGG
CGAGCCGCAGCCATTGCCTTTTATGGTAATCGTGCGAGAGGGCGCAGGGACTTCCTTTGTCCCAAATC
TGGCGGAGCCGAAATCTGGGAGGCGCCGCCGCACCCCCTCTAGCGGGCGCGGGCGAAGCGGTGCGGCG
CCGGCAGGAAGGAAATGGGCGGGGAGGGCCTTCGTGCGTCGCCGCGCCGCCGTCCCCTTCTCCATCTC
CAGCCTCGGGGCTGCCGCAGGGGGACGGCTGCCTTCGGGGGGGACGGGGCAGGGCGGGGTTCGGCTTC
TGGCGTGTGACCGGCGGCTCTAGAGCCTCTGCTAACCATGTTCATGCCTTCTTCTTTTTCCTACAGCT
CCTGGGCAACGTGCTGGTTGTTGTGCTGTCTCATCATTTTGGCAAAGAATTATCGCATGCCTGCAGAG
CTCTAGAGTCCCGGTGCCACCATGGCCCCAAAGAAGAAGCGGAAGGTCGGTATCCACGGAGTCCCAGC
AGCCAAGCGGAACTACATCCTGGGCCTGGACATCGGCATCACCAGCGTGGGCTACGGCATCATCGACT
ACGAGACACGGGACGTGATCGATGCCGGCGTGCGGCTGTTCAAAGAGGCCAACGTGGAAAACAACGAG
GGCAGGCGGAGCAAGAGAGGCGCCAGAAGGCTGAAGCGGCGGAGGCGGCATAGAATCCAGAGAGTGAA
GAAGCTGCTGTTCGACTACAACCTGCTGACCGACCACAGCGAGCTGAGCGGCATCAACCCCTACGAGG
CCAGAGTGAAGGGCCTGAGCCAGAAGCTGAGCGAGGAAGAGTTCTCTGCCGCCCTGCTGCACCTGGCC
AAGAGAAGAGGCGTGCACAACGTGAACGAGGTGGAAGAGGACACCGGCAACGAGCTGTCCACCAAAGA
GCAGATCAGCCGGAACAGCAAGGCCCTGGAAGAGAAATACGTGGCCGAACTGCAGCTGGAACGGCTGA
AGAAAGACGGCGAAGTGCGGGGCAGCATCAACAGATTCAAGACCAGCGACTACGTGAAAGAAGCCAAA
CAGCTGCTGAAGGTGCAGAAGGCCTACCACCAGCTGGACCAGAGCTTCATCGACACCTACATCGACCT
GCTGGAAACCCGGCGGACCTACTATGAGGGACCTGGCGAGGGCAGCCCCTTCGGCTGGAAGGACATCA
AAGAATGGTACGAGATGCTGATGGGCCACTGCACCTACTTCCCCGAGGAACTGCGGAGCGTGAAGTAC
GCCTACAACGCCGACCTGTACAACGCCCTGAACGACCTGAACAATCTCGTGATCACCAGGGACGAGAA
CGAGAAGCTGGAATATTACGAGAAGTTCCAGATCATCGAGAACGTGTTCAAGCAGAAGAAGAAGCCCA
CCCTGAAGCAGATCGCCAAAGAAATCCTCGTGAACGAAGAGGATATTAAGGGCTACAGAGTGACCAGC
ACCGGCAAGCCCGAGTTCACCAACCTGAAGGTGTACCACGACATCAAGGACATTACCGCCCGGAAAGA
GATTATTGAGAACGCCGAGCTGCTGGATCAGATTGCCAAGATCCTGACCATCTACCAGAGCAGCGAGG
ACATCCAGGAAGAACTGACCAATCTGAACTCCGAGCTGACCCAGGAAGAGATCGAGCAGATCTCTAAT
CTGAAGGGCTATACCGGCACCCACAACCTGAGCCTGAAGGCCATCAACCTGATCCTGGACGAGCTGTG
GCACACCAACGACAACCAGATCGCTATCTTCAACCGGCTGAAGCTGGTGCCCAAGAAGGTGGACCTGT
CCCAGCAGAAAGAGATCCCCACCACCCTGGTGGACGACTTCATCCTGAGCCCCGTCGTGAAGAGAAGC
TTCATCCAGAGCATCAAAGTGATCAACGCCATCATCAAGAAGTACGGCCTGCCCAACGACATCATTAT
CGAGCTGGCCCGCGAGAAGAACTCCAAGGACGCCCAGAAAATGATCAACGAGATGCAGAAGCGGAACC
GGCAGACCAACGAGCGGATCGAGGAAATCATCCGGACCACCGGCAAAGAGAACGCCAAGTACCTGATC
GAGAAGATCAAGCTGCACGACATGCAGGAAGGCAAGTGCCTGTACAGCCTGGAAGCCATCCCTCTGGA
AGATCTGCTGAACAACCCCTTCAACTATGAGGTGGACCACATCATCCCCAGAAGCGTGTCCTTCGACA
ACAGCTTCAACAACAAGGTGCTCGTGAAGCAGGAAGAAGCCAGCAAGAAGGGCAACCGGACCCCATTC
CAGTACCTGAGCAGCAGCGACAGCAAGATCAGCTACGAAACCTTCAAGAAGCACATCCTGAATCTGGC
CAAGGGCAAGGGCAGAATCAGCAAGACCAAGAAAGAGTATCTGCTGGAAGAACGGGACATCAACAGGT
TCTCCGTGCAGAAAGACTTCATCAACCGGAACCTGGTGGATACCAGATACGCCACCAGAGGCCTGATG
AACCTGCTGCGGAGCTACTTCAGAGTGAACAACCTGGACGTGAAAGTGAAGTCCATCAATGGCGGCTT
CACCAGCTTTCTGCGGCGGAAGTGGAAGTTTAAGAAAGAGCGGAACAAGGGGTACAAGCACCACGCCG
AGGACGCCCTGATCATTGCCAACGCCGATTTCATCTTCAAAGAGTGGAAGAAACTGGACAAGGCCAAA
AAAGTGATGGAAAACCAGATGTTCGAGGAAAAGCAGGCCGAGAGCATGCCCGAGATCGAAACCGAGCA
GGAGTACAAAGAGATCTTCATCACCCCCCACCAGATCAAGCACATTAAGGACTTCAAGGACTACAAGT
ACAGCCACCGGGTGGACAAGAAGCCTAATAGAGAGCTGATTAACGACACCCTGTACTCCACCCGGAAG
GACGACAAGGGCAACACCCTGATCGTGAACAATCTGAACGGCCTGTACGACAAGGACAATGACAAGCT
GAAAAAGCTGATCAACAAGAGCCCCGAAAAGCTGCTGATGTACCACCACGACCCCCAGACCTACCAGA
AACTGAAGCTGATTATGGAACAGTACGGCGACGAGAAGAATCCCCTGTACAAGTACTACGAGGAAACC
GGGAACTACCTGACCAAGTACTCCAAAAAGGACAACGGCCCCGTGATCAAGAAGATTAAGTATTACGG
CAACAAACTGAACGCCCATCTGGACATCACCGACGACTACCCCAACAGCAGAAACAAGGTCGTGAAGC

TGTCCCTGAAGCCCTACAGATTCGACGTGTACCTGGACAATGGCGTGTACAAGTTCGTGACCGTGAAG
AATCTGGATGTGATCAAAAAAGAAAACTACTACGAAGTGAATAGCAAGTGCTATGAGGAAGCTAAGAA
GCTGAAGAAGATCAGCAACCAGGCCGAGTTTATCGCCTCCTTCTACAACAACGATCTGATCAAGATCA
ACGGCGAGCTGTATAGAGTGATCGGCGTGAACAACGACCTGCTGAACCGGATCGAAGTGAACATGATC
GACATCACCTACCGCGAGTACCTGGAAAACATGAACGACAAGAGGCCCCCCAGGATCATTAAGACAAT
CGCCTCCAAGACCCAGAGCATTAAGAAGTACAGCACAGACATTCTGGGCAACCTGTATGAAGTGAAAT
CTAAGAAGCACCCTCAGATCATCAAAAAGGGCAAAAGGCCGGCGGCCACGAAAAAGGCCGGCCAGGCA
AAAAAGAAAAAGGGATCCTACCCATACGATGTTCCAGATTACGCTTATCCCTACGACGTGCCTGATTA
TGCATACCCATATGATGTCCCCGACTATGCCTAAGAATTGGCCGCACTTAAGTTACGCGTGGATCAAT
TCACTCCTCAGGTGCAGGCTGCCTATCAGAAGGTGGTGGCTGGTGTGGCCAATGCCCTGGCTCACAAA
TACCACTGAGATCTTTTTCCCTCTGCCAAAAATTATGGGGACATCATGAAGCCCCTTGAGCATCTGAC
TTCTGGCTAATAAAGGAAATTTATTTTCATTGCAATAGTGTGTTGGAATTTTTTGTGTCTCTCACTCG
GAAGGACATATGGGAGGGCAAATCATTTAAAACATCAGAATGAGTATTTGGTTTAGAGTTTGGCAACA
TATGCCATATGCTGGCTGCCATGAACAAAGGTGGCTATAAAGAGGTCATCAGTATATGAAACAGCCCC
CTGCTGTCCATTCCTTATTCCATAGAAAAGCCTTGACTTGAGGTTAGATTTTTTTTATATTTTGTTTT
GTGTTATTTTTTTCTTTAACATCCCTAAAATTTTCCTTACATGTTTTACTAGCCAGATTTTTCCTCCT
CTCCTGACTACTCCCAGTCATAGCTGTCCCTCTTCTCTTATGAAGATCCCTCGACCTGCAGCCCAAGC
TGATCCCGGGATTTAAATGTTTAAACGAATTCACTGGCCGTCGTTTTACAACGTCGTGACTGGGAAAA
CCCTGGCGTTACCCAACTTAATCGCCTTGCAGCACATCCCCCTTTCGCCAGCTGGCGTAATAGCGAAG
AGGCCCGCACCGATCGCCCTTCCCAACAGTTGCGCAGCCTGAATGGCGAATGGCGCCTGATGCGGTAT
TTTCTCCTTACGCATCTGTGCGGTATTTCACACCGCATATGGTGCACTCTCAGTACAATCTGCTCTGA
TGCCGCATAGTTAAGCCAGCCCCGACACCCGCCAACACCCGCTGACGCGCCCTGACGGGCTTGTCTGC
TCCCGGCATCCGCTTACAGACAAGCTGTGACCGTCTCCGGGAGCTGCATGTGTCAGAGGTTTTCACCG
TCATCACCGAAACGCGCGAGACGAAAGGGCCTCGTGATACGCCTATTTTTATAGGTTAATGTCATGAT
AATAATGGTTTCTTAGACGTCAGGTGGCACTTTTCGGGGAAATGTGCGCGGAACCCCTATTTGTTTAT
TTTTCTAAATACATTCAAATATGTATCCGCTCATGAGACAATAACCCTGATAAATGCTTCAATAATAT
TGAAAAAGGAAGAGTATGAGTATTCAACATTTCCGTGTCGCCCTTATTCCCTTTTTTGCGGCATTTTG
CCTTCCTGTTTTTGCTCACCCAGAAACGCTGGTGAAAGTAAAAGATGCTGAAGATCAGTTGGGTGCAC
GAGTGGGTTACATCGAACTGGATCTCAACAGCGGTAAGATCCTTGAGAGTTTTCGCCCCGAAGAACGT
TTTCCAATGATGAGCACTTTTAAAGTTCTGCTATGTGGCGCGGTATTATCCCGTATTGACGCCGGGCA
AGAGCAACTCGGTCGCCGCATACACTATTCTCAGAATGACTTGGTTGAGTACTCACCAGTCACAGAAA
AGCATCTTACGGATGGCATGACAGTAAGAGAATTATGCAGTGCTGCCATAACCATGAGTGATAACACT
GCGGCCAACTTACTTCTGACAACGATCGGAGGACCGAAGGAGCTAACCGCTTTTTTGCACAACATGGG
GGATCATGTAACTCGCCTTGATCGTTGGGAACCGGAGCTGAATGAAGCCATACCAAACGACGAGCGTG
ACACCACGATGCCTGTAGCAATGGCAACAACGTTGCGCAAACTATTAACTGGCGAACTACTTACTCTA
GCTTCCCGGCAACAATTAATAGACTGGATGGAGGCGGATAAAGTTGCAGGACCACTTCTGCGCTCGGC
CCTTCCGGCTGGCTGGTTTATTGCTGATAAATCTGGAGCCCGTGAGCGTGGGTCTCGCGGTATCATTG
CAGCACTGGGGCCAGATGGTAAGCCCTCCCGTATCGTAGTTATCTACACGACGGGGAGTCAGGCAACT
ATGGATGAACGAAATAGACAGATCGCTGAGATAGGTGCCTCACTGATTAAGCATTGGTAACTGTCAGA
CCAAGTTTACTCATATATACTTTAGATTGATTTAAAACTTCATTTTTAATTTAAAAGGATCTAGGTGA
AGATCCTTTTTGATAATCTCATGACCAAAATCCCTTAACGTGAGTTTTCGTTCCACTGAGCGTCAGAC
CCCGTAGAAAAGATCAAAGGATCTTCTTGAGATCCTTTTTTTCTGCGCGTAATCTGCTGCTTGCAAAC
AAAAAAACCACCGCTACCAGCGGTGGTTTGTTTGCCGGATCAAGAGCTACCAACTCTTTTTCCGAAGG
TAACTGGCTTCAGCAGAGCGCAGATACCAAATACTGTCCTTCTAGTGTAGCCGTAGTTAGGCCACCAC
TTCAAGAACTCTGTAGCACCGCCTACATACCTCGCTCTGCTAATCCTGTTACCAGTGGCTGCTGCCAG
TGGCGATAAGTCGTGTCTTACCGGGTTGGACTCAAGACGATAGTTACCGGATAAGGCGCAGCGGTCGG
GCTGAACGGGGGGTTCGTGCACACAGCCCAGCTTGGAGCGAACGACCTACACCGAACTGAGATACCTA
CAGCGTGAGCTATGAGAAAGCGCCACGCTTCCCGAAGGGAGAAAGGCGGACAGGTATCCGGTAAGCGG

```
CAGGGTCGGAACAGGAGAGCGCACGAGGGAGCTTCCAGGGGGAAACGCCTGGTATCTTTATAGTCCTG
TCGGGTTTCGCCACCTCTGACTTGAGCGTCGATTTTTGTGATGCTCGTCAGGGGGGCGGAGCCTATGG
AAAAACGCCAGCAACGCGGCCTTTTTACGGTTCCTGGCCTTTTGCTGGCCTTTTGCTCACATGTTCTT
TCCTGCGTTATCCCCTGATTCTGTGGATAACCGTATTACCGCCTTTGAGTGAGCTGATACCGCTCGCC
GCAGCCGAACGACCGAGCGCAGCGAGTCAGTGAGCGAGGAAGCGGAAGAGCGCCCAATACGCAAACCG
CCTCTCCCCGCGCGTTGGCCGATTCATTAATGCAGCTGGCACGACAGGTTTCCCGACTGGAAAGCGGG
CAGTGAGCGCAACGCAATTAATGTGAGTTAGCTCACTCATTAGGCACCCCAGGCTTTACACTTTATGC
TTCCGGCTCGTATGTTGTGTGGAATTGTGAGCGGATAACAATTTCACACAGGAAACAGCTATGACCAT
GATTACGCCAAGCTT
```

**Map and nucleotide sequence of expression plasmid for orthogonal SaCas9$^{N580A}$ nickase BA32_pU.CAG.SaCas9$^{N580A}$.** CAG, hybrid promoter (CMV enhancer, human cytomegalovirus *immediate-early* gene enhancer; chicken *β-actin* promoter; chimeric intron, fusion between introns from the chicken *β-actin* and rabbit *β-globin* genes); SaCas9$^{N580A}$, human codon-optimized ORF coding for the SaCas9 nickase derived from the *Staphylococcus aureus* type II CRISPR system; rBGpA, rabbit *β-globin* polyadenylation signal; AmpR, *β-lactamase* ampicillin resistance gene. The SaCas9$^{N580A}$ coding sequence is highlighted in magenta. The sequences coding for the HNH-disabling mutation N580A and the indicated nuclear localization signals are marked in yellow and blue, respectively.

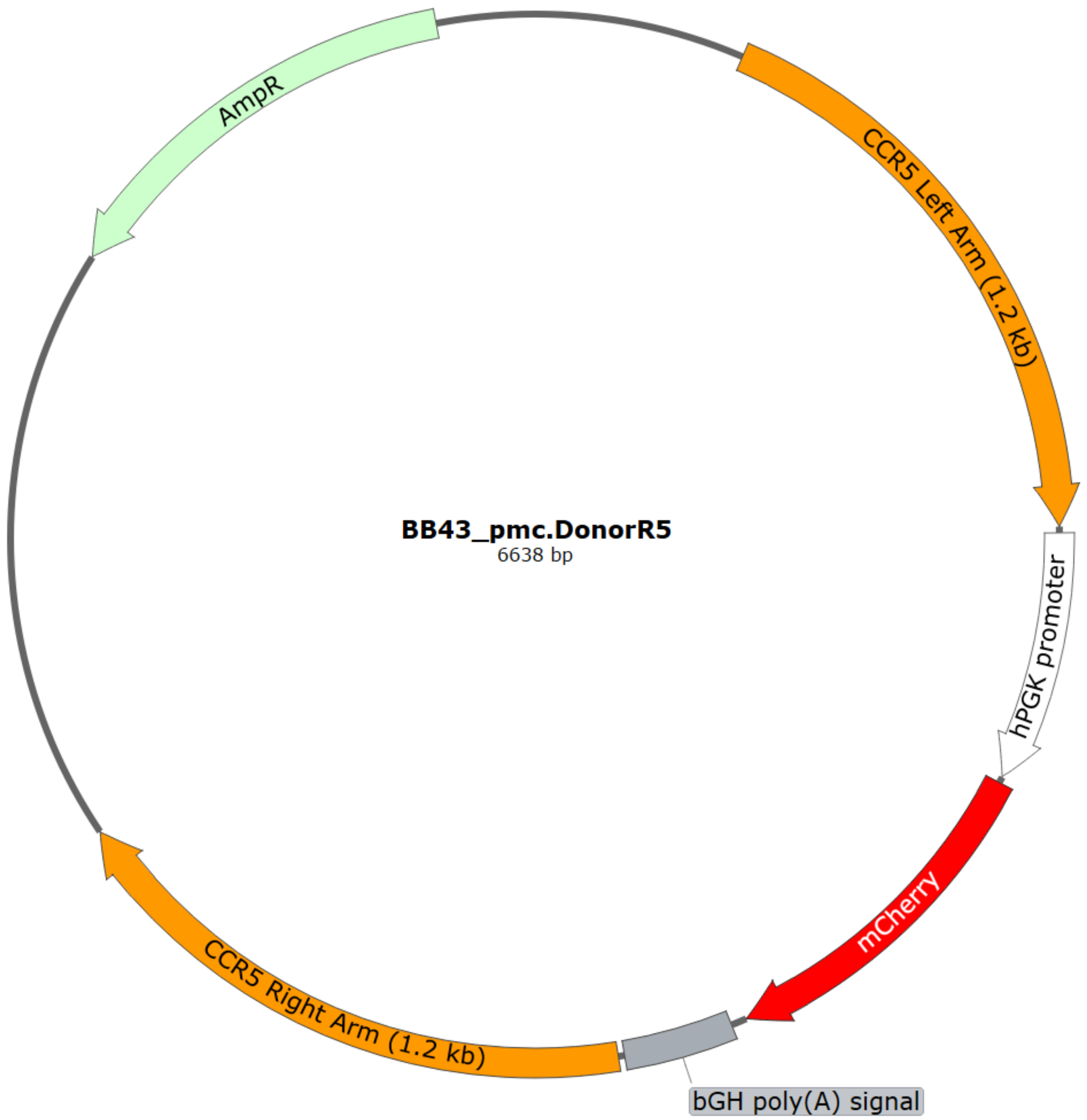


>BB43_pmc.Donor$^{R5}$ (6638 bp)

TCGCGCGTTTCGGTGATGACGGTGAAAACCTCTGACACATGCAGCTCCCGGAGACGGTCACAGCTTGT
CTGTAAGCGGATGCCGGGAGCAGACAAGCCCGTCAGGGCGCGTCAGCGGGTGTTGGCGGGTGTCGGGG
CTGGCTTAACTATGCGGCATCAGAGCAGATTGTACTGAGAGTGCACCATATGCGGTGTGAAATACCGC
ACAGATGCGTAAGGAGAAAATACCGCATCAGGCGCCATTCGCCATTCAGGCTGCGCAACTGTTGGGAA
GGGCGATCGGTGCGGGCCTCTTCGCTATTACGCCAGCTGGCGAAAGGGGGATGTGCTGCAAGGCGATT
AAGTTGGGTAACGCCAGGGTTTTCCCAGTCACGACGTTGTAAAACGACGGCCAGTGAATTCGAGCTCG
GTACCTCGCGAATGCATCTAGTGTTTCATGGCACAGTTAGTATATAATTCTTTATGGTTCAAAATTAA
AAATGAGCTTTTCTAGGGGCTTCTCTCAGCTGCCTAGTCTAAGGTGCAGGGAGTTTGAGACTCACAGG
GTTTAATAAGAGAAAATTCTCAGCTAGAGCAGCTGAACTTAAATAGACTAGGCAAGACAGCTGGTTAT
AAGACTAAACTACCCAGAATGCATGACATTCATCTGTGGTGGCAGACGAAACATTTTTTATTATATTA
TTTCTTGGGTATGTATGACAACTCTTAATTGTGGCAACTCAGAAACTACAAACACAAACTTCACAGAA
AATGTGAGGATTTTACAATTGGCTGTTGTCATCTATGACCTTCCCTGGGACTTGGGCACCCGGCCATT
TCACTCTGACTACATCATGTCACCAAACATCTGATGGTCTTGCCTTTTAATTCTCTTTTCGAGGACTG
AGAGGGAGGGTAGCATGGTAGTTAAGAGTGCAGGCTTCCCGCATTCAAAATCGGTTGCTTACTAGCTG

TGTGGCTTTGAGCAAGTTACTCACCCTCTCTGTGCTTCAAGGTCCTTGTCTGCAAAATGTGAAAAATA
TTTCCTGCCTCATAAGGTTGCCCTAAGGATTAAATGAATGAATGGGTATGATGCTTAGAACAGTGATT
GGCATCCAGTATGTGCCCTCGAGGCCTCTTAATTATTACTGGCTTGCTCATAGTGCATGTTCTTTGTG
GGCTAACTCTAGCGTCAATAAAAATGTTAAGACTGAGTTGCAGCCGGGCATGGTGGCTCATGCCTGTA
ATCCCAGCATTCTAGGAGGCTGAGGCAGGAGGATCGCTTGAGCCCAGGAGTTCGAGACCAGCCTGGGC
AACATAGTGTGATCTTGTATCTATAAAAATAAACAAAATTAGCTTGGTGTGGTGGCGCCTGTAGTCCC
CAGCCACTTGGAGGGGTGAGGTGAGAGGATTGCTTGAGCCCGGGATGGTCCAGGCTGCAGTGAGCCAT
GATCGTGCCACTGCACTCCAGCCTGGGCGACAGAGTGAGACCCTGTCTCACAACAACAACAACAACAA
CAAAAAGGCTGAGCTGCACCATGCTTGACCCAGTTTCTTAAAATTGTTGTCAAAGCTTCATTCACTCC
ATGGTGCTATAGAGCACAAGATTTTATTTGGTGAGATGGTGCTTTCATGAATTCCCCCAACAGAGCAA
GCTTGTTAGCTTCCACGGGGTTGGGGTTGCGCCTTTTCCAAGGCAGCCCTGGGTTTGCGCAGGGACGC
GGCTGCTCTGGGCGTGGTTCCGGGAAACGCAGCGGCGCCGACCCTGGGTCTCGCACATTCTTCACGTC
CGTTCGCAGCGTCACCCGGATCTTCGCCGCTACCCTTGTGGGCCCCCCGGCGACGCTTCCTGCTCCGC
CCCTAAGTCGGGAAGGTTCCTTGCGGTTCGCGGCGTGCCGGACGTGACAAACGGAAGCCGCACGTCTC
ACTAGTACCCTCGCAGACGGACAGCGCCAGGGAGCAATGGCAGCGCGCCGACCGCGATGGGCTGTGGC
CAATAGCGGCTGCTCAGCAGGGCGCGCCGAGAGCAGCGGCCGGGAAGGGGCGGTGCGGGAGGCGGGGT
GTGGGGCGGTAGTGTGGGCCCTGTTCCTGCCCGCGCGGTGTTCCGCATTCTGCAAGCCTCCGGAGCGC
ACGTCGGCAGTCGGCTCCCTCGTTGACCGAATCACCGACCTCTCTCCCCAAGATCTGCCACCATGGTG
AGCAAGGGCGAGGAGGATAACATGGCCATCATCAAGGAGTTCATGCGCTTCAAGGTGCACATGGAGGG
CTCCGTGAACGGCCACGAGTTCGAGATCGAGGGCGAGGGCGAGGGCCGCCCCTACGAGGGCACCCAGA
CCGCCAAGCTGAAGGTGACCAAGGGTGGCCCCCTGCCCTTCGCCTGGGACATCCTGTCCCCTCAGTTC
ATGTACGGCTCCAAGGCCTACGTGAAGCACCCCGCCGACATCCCCGACTACTTGAAGCTGTCCTTCCC
CGAGGGCTTCAAGTGGGAGCGCGTGATGAACTTCGAGGACGGCGGCGTGGTGACCGTGACCCAGGACT
CCTCCCTGCAGGACGGCGAGTTCATCTACAAGGTGAAGCTGCGCGGCACCAACTTCCCCTCCGACGGC
CCCGTAATGCAGAAGAAGACCATGGGCTGGGAGGCCTCCTCCGAGCGGATGTACCCCGAGGACGGCGC
CCTGAAGGGCGAGATCAAGCAGAGGCTGAAGCTGAAGGACGGCGGCCACTACGACGCTGAGGTCAAGA
CCACCTACAAGGCCAAGAAGCCCGTGCAGCTGCCCGGCGCCTACAACGTCAACATCAAGTTGGACATC
ACCTCCCACAACGAGGACTACACCATCGTGGAACAGTACGAACGCGCCGAGGGCCGCCACTCCACCGG
CGGCATGGACGAGCTGTACAAGTAAAGCGGCCGCGTCGAGTCTAGGATCAGCCTCGACTGTGCCTTCT
AGTTGCCAGCCATCTGTTGTTTGCCCCTCCCCCGTGCCTTCCTTGACCCTGGAAGGTGCCACTCCCAC
TGTCCTTTCCTAATAAAATGAGGAAATTGCATCGCATTGTCTGAGTAGGTGTCATTCTATTCTGGGGG
GTGGGGTGGGGCAGGACAGCAAGGGGGAGGATTGGGAAGACAATAGCAGGCATGCTGGGGATGCGGTG
GGCTCTATGGAAGCTAACACGCGTCTCCATCTAGTGGACAGGGAAGCTAGCAGCAAACCTTCCCTTCA
CTACAAAACTTCATTGCTTGGCCAAAAAGAGAGTTAATTCAATGTAGACATCTATGTAGGCAATTAAA
AACCTATTGATGTATAAAACAGTTTGCATTCATGGAGGGCAACTAAATACATTCTAGGACTTTATAAA
AGATCACTTTTTATTTATGCACAGGGTGGAACAAGATGGATTATCAAGTGTCAAGTCCAATCTATGAC
ATCAATTATTATACATCGGAGCCCTGCCAAAAAATCAATGTGAAGCAAATCGCAGCCCGCCTCCTGCC
TCCGCTCTACTCACTGGTGTTCATCTTTGGTTTTGTGGGCAACATGCTGGTCATCCTCATCCTGATAA
ACTGCAAAAGGCTGAAGAGCATGACTGACATCTACCTGCTCAACCTGGCCATCTCTGACCTGTTTTTC
CTTCTTACTGTCCCCTTCTGGGCTCACTATGCTGCCGCCCAGTGGGACTTTGGAAATACAATGTGTCA
ACTCTTGACAGGGCTCTATTTTATAGGCTTCTTCTCTGGAATCTTCTTCATCATCCTCCTGACAATCG
ATAGGTACCTGGCTGTCGTCCATGCTGTGTTTGCTTTAAAAGCCAGGACGGTCACCTTTGGGGTGGTG
ACAAGTGTGATCACTTGGGTGGTGGCTGTGTTTGCGTCTCTCCCAGGAATCATCTTTACCAGATCTCA
AAAAGAAGGTCTTCATTACACCTGCAGCTCTCATTTTCCATACAGTCAGTATCAATTCTGGAAGAATT
TCCAGACATTAAAGATAGTCATCTTGGGGCTGGTCCTGCCGCTGCTTGTCATGGTCATCTGCTACTCG
GGAATCCTAAAAACTCTGCTTCGGTGTCGAAATGAGAAGAAGAGGCACAGGGCTGTGAGGCTTATCTT
CACCATCATGATTGTTTATTTTCTCTTCTGGGCTCCCTACAACATTGTCCTTCTCCTGAACACCTTCC
AGGAATTCTTTGGCCTGAATAATTGCAGTAGCTCTAACAGGTTGGACCAAGCTATGCAGGTGACAGAG

ACTCTTGGGATGACGCACTGCTGCATCAACCCCATCATCTATGCCTTTGTCGGGGAGAAGTTCAGAAA
CTACCTCTTAGTCTTCTTCCAAAAGCACATTGCCAAACGCTTCTGCAAATGCTGTTCTATTTTCCAGC
CCTAGATATCGGATCCCGGGCCCGTCGACTGCAGAGGCCTGCATGCAAGCTTGGCGTAATCATGGTCA
TAGCTGTTTCCTGTGTGAAATTGTTATCCGCTCACAATTCCACACAACATACGAGCCGGAAGCATAAA
GTGTAAAGCCTGGGGTGCCTAATGAGTGAGCTAACTCACATTAATTGCGTTGCGCTCACTGCCCGCTT
TCCAGTCGGGAAACCTGTCGTGCCAGCTGCATTAATGAATCGGCCAACGCGCGGGGAGAGGCGGTTTG
CGTATTGGGCGCTCTTCCGCTTCCTCGCTCACTGACTCGCTGCGCTCGGTCGTTCGGCTGCGGCGAGC
GGTATCAGCTCACTCAAAGGCGGTAATACGGTTATCCACAGAATCAGGGGATAACGCAGGAAAGAACA
TGTGAGCAAAAGGCCAGCAAAAGGCCAGGAACCGTAAAAAGGCCGCGTTGCTGGCGTTTTTCCATAGG
CTCCGCCCCCCTGACGAGCATCACAAAAATCGACGCTCAAGTCAGAGGTGGCGAAACCCGACAGGACT
ATAAAGATACCAGGCGTTTCCCCCTGGAAGCTCCCTCGTGCGCTCTCCTGTTCCGACCCTGCCGCTTA
CCGGATACCTGTCCGCCTTTCTCCCTTCGGGAAGCGTGGCGCTTTCTCATAGCTCACGCTGTAGGTAT
CTCAGTTCGGTGTAGGTCGTTCGCTCCAAGCTGGGCTGTGTGCACGAACCCCCCGTTCAGCCCGACCG
CTGCGCCTTATCCGGTAACTATCGTCTTGAGTCCAACCCGGTAAGACACGACTTATCGCCACTGGCAG
CAGCCACTGGTAACAGGATTAGCAGAGCGAGGTATGTAGGCGGTGCTACAGAGTTCTTGAAGTGGTGG
CCTAACTACGGCTACACTAGAAGAACAGTATTTGGTATCTGCGCTCTGCTGAAGCCAGTTACCTTCGG
AAAAAGAGTTGGTAGCTCTTGATCCGGCAAACAAACCACCGCTGGTAGCGGTGGTTTTTTTGTTTGCA
AGCAGCAGATTACGCGCAGAAAAAAAGGATCTCAAGAAGATCCTTTGATCTTTTCTACGGGGTCTGAC
GCTCAGTGGAACGAAAACTCACGTTAAGGGATTTTGGTCATGAGATTATCAAAAAGGATCTTCACCTA
GATCCTTTTAAATTAAAAATGAAGTTTTAAATCAATCTAAAGTATATATGAGTAAACTTGGTCTGACA
GTTACCAATGCTTAATCAGTGAGGCACCTATCTCAGCGATCTGTCTATTTCGTTCATCCATAGTTGCC
TGACTCCCCGTCGTGTAGATAACTACGATACGGGAGGGCTTACCATCTGGCCCCAGTGCTGCAATGAT
ACCGCGAGACCCACGCTCACCGGCTCCAGATTTATCAGCAATAAACCAGCCAGCCGGAAGGGCCGAGC
GCAGAAGTGGTCCTGCAACTTTATCCGCCTCCATCCAGTCTATTAATTGTTGCCGGGAAGCTAGAGTA
AGTAGTTCGCCAGTTAATAGTTTGCGCAACGTTGTTGCCATTGCTACAGGCATCGTGGTGTCACGCTC
GTCGTTTGGTATGGCTTCATTCAGCTCCGGTTCCCAACGATCAAGGCGAGTTACATGATCCCCCATGT
TGTGCAAAAAAGCGGTTAGCTCCTTCGGTCCTCCGATCGTTGTCAGAAGTAAGTTGGCCGCAGTGTTA
TCACTCATGGTTATGGCAGCACTGCATAATTCTCTTACTGTCATGCCATCCGTAAGATGCTTTTCTGT
GACTGGTGAGTACTCAACCAAGTCATTCTGAGAATAGTGTATGCGGCGACCGAGTTGCTCTTGCCCGG
CGTCAATACGGGATAATACCGCGCCACATAGCAGAACTTTAAAAGTGCTCATCATTGGAAAACGTTCT
TCGGGGCGAAAACTCTCAAGGATCTTACCGCTGTTGAGATCCAGTTCGATGTAACCCACTCGTGCACC
CAACTGATCTTCAGCATCTTTTACTTTCACCAGCGTTTCTGGGTGAGCAAAAACAGGAAGGCAAAATG
CCGCAAAAAAGGGAATAAGGGCGACACGGAAATGTTGAATACTCATACTCTTCCTTTTTCAATATTAT
TGAAGCATTTATCAGGGTTATTGTCTCATGAGCGGATACATATTTGAATGTATTTAGAAAAATAAACA
AATAGGGGTTCCGCGCACATTTCCCCGAAAAGTGCCACCTGACGTCTAAGAAACCATTATTATCATGA
CATTAACCTATAAAAATAGGCGTATCACGAGGCCCTTTCGTC

**Map and nucleotide sequence of *CCR5*-targeting plasmid BB43_pmc.Donor$^{R5}$**. Orange regions, sequences homologous to the human *CCR5* safe harbour locus; hPGK promoter, human phosphoglycerate kinase 1 gene (*PGK1*) regulatory sequences; mCherry, open reading frame of a monomeric derivative of DsRed fluorescent protein; bGH poly(A) signal, bovine growth hormone gene (*GH1*) polyadenylation signal; AmpR, *β-lactamase* ampicillin resistance gene.

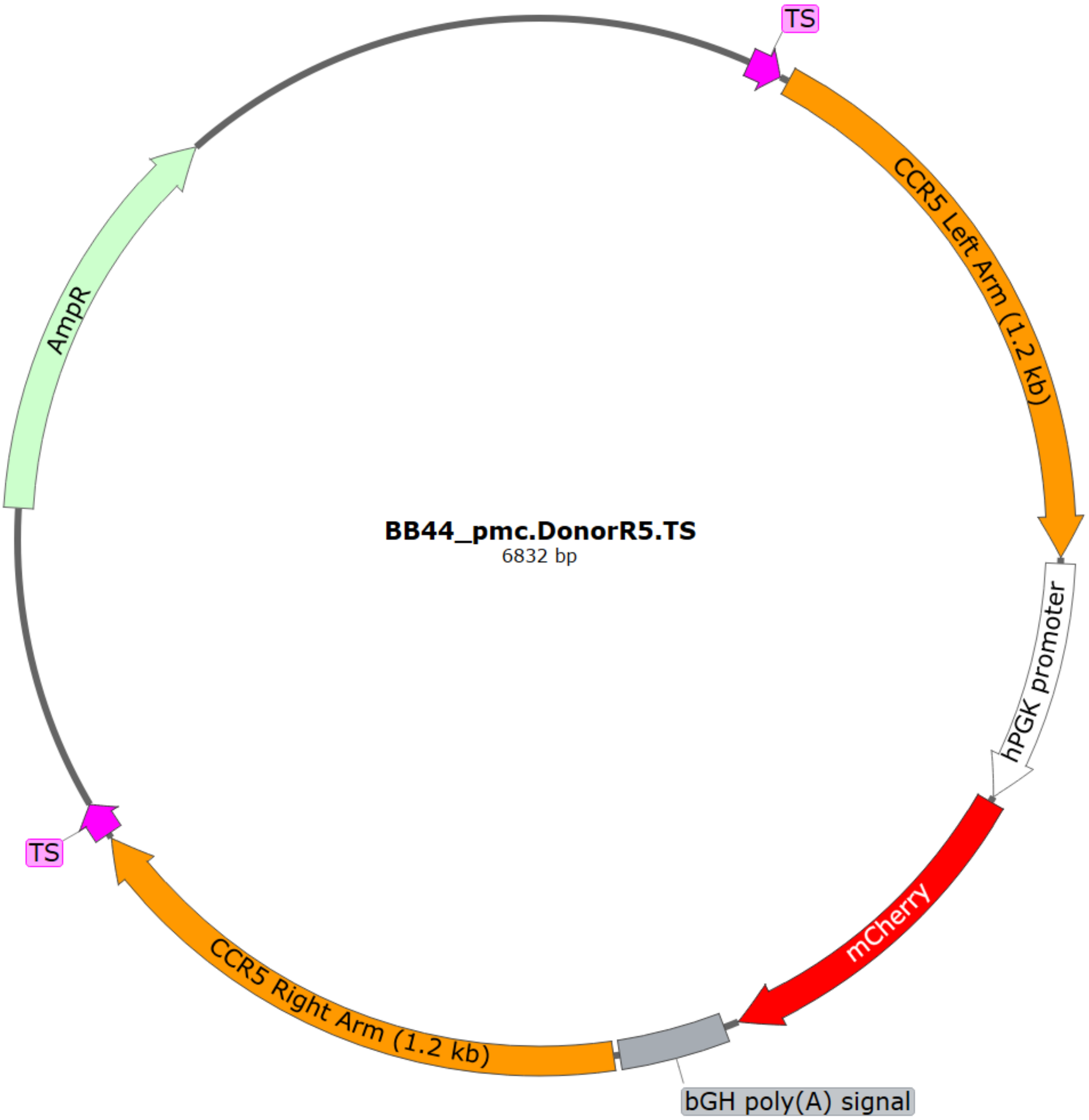


>BB44_pmc.Donor$^{R5.TS}$ (6832 bp)

TACGGTTCCTGGCCTTTTGCTGGCCTTTTGCTCACATGTTCTTTCCTGCGTTATCCCCTGATTCTGTG
GATAACCGTATTACCGCCTTTGAGTGAGCTGATACCGCTCGCCGCAGCCGAACGACCGAGCGCAGCGA
GTCAGTGAGCGAGGAAGCGGAAGAGCGCCCAATACGCAAACCGCCTCTCCCCGCGCGTTGGCCGATTC
ATTAATGCAGCTGGCACGACAGGTTTCCCGACTGGAAAGCGGGCAGTGAGCGCAACGCAATTAATGTG
AGTTAGCTCACTCATTAGGCACCCCAGGCTTTACACTTTATGCTTCCGGCTCGTATGTTGTGTGGAAT
TGTGAGCGGATAACAATTTCACACAGGAAACAGCTATGACCATGATTACGCCAAGCTTGCATGCAGGC
CTCTGCAGTCGACGGGCCCGGGATCCGATACGACGCGGCCGCAGATGGTGCTTTCATGAATTCCCCCA
ACAGAGCCAAGCTCTCCATCTAGTGGACAGGGAAGCTAGCAGCAAAATTTCGAATGCATCTAGTGTTT
CATGGCACAGTTAGTATATAATTCTTTATGGTTCAAAATTAAAAATGAGCTTTTCTAGGGGCTTCTCT
CAGCTGCCTAGTCTAAGGTGCAGGGAGTTTGAGACTCACAGGGTTTAATAAGAGAAAATTCTCAGCTA
GAGCAGCTGAACTTAAATAGACTAGGCAAGACAGCTGGTTATAAGACTAAACTACCCAGAATGCATGA
CATTCATCTGTGGTGGCAGACGAAACATTTTTTATTATATTATTTCTTGGGTATGTATGACAACTCTT
AATTGTGGCAACTCAGAAACTACAAACACAAACTTCACAGAAAATGTGAGGATTTTACAATTGGCTGT
TGTCATCTATGACCTTCCCTGGGACTTGGGCACCCGGCCATTTCACTCTGACTACATCATGTCACCAA
ACATCTGATGGTCTTGCCTTTTAATTCTCTTTTCGAGGACTGAGAGGGAGGGTAGCATGGTAGTTAAG

AGTGCAGGCTTCCCGCATTCAAAATCGGTTGCTTACTAGCTGTGTGGCTTTGAGCAAGTTACTCACCC
TCTCTGTGCTTCAAGGTCCTTGTCTGCAAAATGTGAAAAATATTTCCTGCCTCATAAGGTTGCCCTAA
GGATTAAATGAATGAATGGGTATGATGCTTAGAACAGTGATTGGCATCCAGTATGTGCCCTCGAGGCC
TCTTAATTATTACTGGCTTGCTCATAGTGCATGTTCTTTGTGGGCTAACTCTAGCGTCAATAAAAATG
TTAAGACTGAGTTGCAGCCGGGCATGGTGGCTCATGCCTGTAATCCCAGCATTCTAGGAGGCTGAGGC
AGGAGGATCGCTTGAGCCCAGGAGTTCGAGACCAGCCTGGGCAACATAGTGTGATCTTGTATCTATAA
AAATAAACAAAATTAGCTTGGTGTGGTGGCGCCTGTAGTCCCCAGCCACTTGGAGGGGTGAGGTGAGA
GGATTGCTTGAGCCCGGGATGGTCCAGGCTGCAGTGAGCCATGATCGTGCCACTGCACTCCAGCCTGG
GCGACAGAGTGAGACCCTGTCTCACAACAACAACAACAACAACAAAAAGGCTGAGCTGCACCATGCTT
GACCCAGTTTCTTAAAATTGTTGTCAAAGCTTCATTCACTCCATGGTGCTATAGAGCACAAGATTTTA
TTTGGTGAGATGGTGCTTTCATGAATTCCCCCAACAGAGCAAGCTTGTTAGCTTCCACGGGGTTGGGG
TTGCGCCTTTTCCAAGGCAGCCCTGGGTTTGCGCAGGGACGCGGCTGCTCTGGGCGTGGTTCCGGGAA
ACGCAGCGGCGCCGACCCTGGGTCTCGCACATTCTTCACGTCCGTTCGCAGCGTCACCCGGATCTTCG
CCGCTACCCTTGTGGGCCCCCCGGCGACGCTTCCTGCTCCGCCCCTAAGTCGGGAAGGTTCCTTGCGG
TTCGCGGCGTGCCGGACGTGACAAACGGAAGCCGCACGTCTCACTAGTACCCTCGCAGACGGACAGCG
CCAGGGAGCAATGGCAGCGCGCCGACCGCGATGGGCTGTGGCCAATAGCGGCTGCTCAGCAGGGCGCG
CCGAGAGCAGCGGCCGGGAAGGGGCGGTGCGGGAGGCGGGGTGTGGGGCGGTAGTGTGGGCCCTGTTC
CTGCCCGCGCGGTGTTCCGCATTCTGCAAGCCTCCGGAGCGCACGTCGGCAGTCGGCTCCCTCGTTGA
CCGAATCACCGACCTCTCTCCCCAAGATCTGCCACCATGGTGAGCAAGGGCGAGGAGGATAACATGGC
CATCATCAAGGAGTTCATGCGCTTCAAGGTGCACATGGAGGGCTCCGTGAACGGCCACGAGTTCGAGA
TCGAGGGCGAGGGCGAGGGCCGCCCCTACGAGGGCACCCAGACCGCCAAGCTGAAGGTGACCAAGGGT
GGCCCCCTGCCCTTCGCCTGGGACATCCTGTCCCCTCAGTTCATGTACGGCTCCAAGGCCTACGTGAA
GCACCCCGCCGACATCCCCGACTACTTGAAGCTGTCCTTCCCCGAGGGCTTCAAGTGGGAGCGCGTGA
TGAACTTCGAGGACGGCGGCGTGGTGACCGTGACCCAGGACTCCTCCCTGCAGGACGGCGAGTTCATC
TACAAGGTGAAGCTGCGCGGCACCAACTTCCCCTCCGACGGCCCCGTAATGCAGAAGAAGACCATGGG
CTGGGAGGCCTCCTCCGAGCGGATGTACCCCGAGGACGGCGCCCTGAAGGGCGAGATCAAGCAGAGGC
TGAAGCTGAAGGACGGCGGCCACTACGACGCTGAGGTCAAGACCACCTACAAGGCCAAGAAGCCCGTG
CAGCTGCCCGGCGCCTACAACGTCAACATCAAGTTGGACATCACCTCCCACAACGAGGACTACACCAT
CGTGGAACAGTACGAACGCGCCGAGGGCCGCCACTCCACCGGCGGCATGGACGAGCTGTACAAGTAAA
GCGGCCGCGTCGAGTCTAGGATCAGCCTCGACTGTGCCTTCTAGTTGCCAGCCATCTGTTGTTTGCCC
CTCCCCCGTGCCTTCCTTGACCCTGGAAGGTGCCACTCCCACTGTCCTTTCCTAATAAAATGAGGAAA
TTGCATCGCATTGTCTGAGTAGGTGTCATTCTATTCTGGGGGGTGGGGTGGGGCAGGACAGCAAGGGG
GAGGATTGGGAAGACAATAGCAGGCATGCTGGGGATGCGGTGGGCTCTATGGAAGCTAACACGCGTCT
CCATCTAGTGGACAGGGAAGCTAGCAGCAAACCTTCCCTTCACTACAAAACTTCATTGCTTGGCCAAA
AAGAGAGTTAATTCAATGTAGACATCTATGTAGGCAATTAAAAACCTATTGATGTATAAAACAGTTTG
CATTCATGGAGGGCAACTAAATACATTCTAGGACTTTATAAAAGATCACTTTTTATTTATGCACAGGG
TGGAACAAGATGGATTATCAAGTGTCAAGTCCAATCTATGACATCAATTATTATACATCGGAGCCCTG
CCAAAAAATCAATGTGAAGCAAATCGCAGCCCGCCTCCTGCCTCCGCTCTACTCACTGGTGTTCATCT
TTGGTTTTGTGGGCAACATGCTGGTCATCCTCATCCTGATAAACTGCAAAAGGCTGAAGAGCATGACT
GACATCTACCTGCTCAACCTGGCCATCTCTGACCTGTTTTTCCTTCTTACTGTCCCCTTCTGGGCTCA
CTATGCTGCCGCCCAGTGGGACTTTGGAAATACAATGTGTCAACTCTTGACAGGGCTCTATTTTATAG
GCTTCTTCTCTGGAATCTTCTTCATCATCCTCCTGACAATCGATAGGTACCTGGCTGTCGTCCATGCT
GTGTTTGCTTTAAAAGCCAGGACGGTCACCTTTGGGGTGGTGACAAGTGTGATCACTTGGGTGGTGGC
TGTGTTTGCGTCTCTCCCAGGAATCATCTTTACCAGATCTCAAAAAGAAGGTCTTCATTACACCTGCA
GCTCTCATTTTCCATACAGTCAGTATCAATTCTGGAAGAATTTCCAGACATTAAAGATAGTCATCTTG
GGGCTGGTCCTGCCGCTGCTTGTCATGGTCATCTGCTACTCGGGAATCCTAAAAACTCTGCTTCGGTG
TCGAAATGAGAAGAAGAGGCACAGGGCTGTGAGGCTTATCTTCACCATCATGATTGTTTATTTTCTCT
TCTGGGCTCCCTACAACATTGTCCTTCTCCTGAACACCTTCCAGGAATTCTTTGGCCTGAATAATTGC

AGTAGCTCTAACAGGTTGGACCAAGCTATGCAGGTGACAGAGACTCTTGGGATGACGCACTGCTGCAT
CAACCCCATCATCTATGCCTTTGTCGGGGAGAAGTTCAGAAACTACCTCTTAGTCTTCTTCCAAAAGC
ACATTGCCAAACGCTTCTGCAAATGCTGTTCTATTTTCCAGCCCTAGATAAACAGATGGTGCTTTCAT
GAATTCCCCCAACAGAGCCAAGCTCTCCATCTAGTGGACAGGGAAGCTAGCAGCAAACGCCGGCGGTC
GTCATCTAGATGCATTCGCGAGGTACCGAGCTCGAATTCACTGGCCGTCGTTTTACAACGTCGTGACT
GGGAAAACCCTGGCGTTACCCAACTTAATCGCCTTGCAGCACATCCCCCTTTCGCCAGCTGGCGTAAT
AGCGAAGAGGCCCGCACCGATCGCCCTTCCCAACAGTTGCGCAGCCTGAATGGCGAATGGCGCCTGAT
GCGGTATTTTCTCCTTACGCATCTGTGCGGTATTTCACACCGCATATGGTGCACTCTCAGTACAATCT
GCTCTGATGCCGCATAGTTAAGCCAGCCCCGACACCCGCCAACACCCGCTGACGCGCCCTGACGGGCT
TGTCTGCTCCCGGCATCCGCTTACAGACAAGCTGTGACCGTCTCCGGGAGCTGCATGTGTCAGAGGTT
TTCACCGTCATCACCGAAACGCGCGAGACGAAAGGGCCTCGTGATACGCCTATTTTTATAGGTTAATG
TCATGATAATAATGGTTTCTTAGACGTCAGGTGGCACTTTTCGGGGAAATGTGCGCGGAACCCCTATT
TGTTTATTTTTCTAAATACATTCAAATATGTATCCGCTCATGAGACAATAACCCTGATAAATGCTTCA
ATAATATTGAAAAAGGAAGAGTATGAGTATTCAACATTTCCGTGTCGCCCTTATTCCCTTTTTTGCGG
CATTTTGCCTTCCTGTTTTTGCTCACCCAGAAACGCTGGTGAAAGTAAAAGATGCTGAAGATCAGTTG
GGTGCACGAGTGGGTTACATCGAACTGGATCTCAACAGCGGTAAGATCCTTGAGAGTTTTCGCCCCGA
AGAACGTTTTCCAATGATGAGCACTTTTAAAGTTCTGCTATGTGGCGCGGTATTATCCCGTATTGACG
CCGGGCAAGAGCAACTCGGTCGCCGCATACACTATTCTCAGAATGACTTGGTTGAGTACTCACCAGTC
ACAGAAAAGCATCTTACGGATGGCATGACAGTAAGAGAATTATGCAGTGCTGCCATAACCATGAGTGA
TAACACTGCGGCCAACTTACTTCTGACAACGATCGGAGGACCGAAGGAGCTAACCGCTTTTTTGCACA
ACATGGGGGATCATGTAACTCGCCTTGATCGTTGGGAACCGGAGCTGAATGAAGCCATACCAAACGAC
GAGCGTGACACCACGATGCCTGTAGCAATGGCAACAACGTTGCGCAAACTATTAACTGGCGAACTACT
TACTCTAGCTTCCCGGCAACAATTAATAGACTGGATGGAGGCGGATAAAGTTGCAGGACCACTTCTGC
GCTCGGCCCTTCCGGCTGGCTGGTTTATTGCTGATAAATCTGGAGCCGGTGAGCGTGGGTCTCGCGGT
ATCATTGCAGCACTGGGGCCAGATGGTAAGCCCTCCCGTATCGTAGTTATCTACACGACGGGGAGTCA
GGCAACTATGGATGAACGAAATAGACAGATCGCTGAGATAGGTGCCTCACTGATTAAGCATTGGTAAC
TGTCAGACCAAGTTTACTCATATATACTTTAGATTGATTTAAAACTTCATTTTTAATTTAAAAGGATC
TAGGTGAAGATCCTTTTTGATAATCTCATGACCAAAATCCCTTAACGTGAGTTTTCGTTCCACTGAGC
GTCAGACCCCGTAGAAAAGATCAAAGGATCTTCTTGAGATCCTTTTTTTCTGCGCGTAATCTGCTGCT
TGCAAACAAAAAAACCACCGCTACCAGCGGTGGTTTGTTTGCCGGATCAAGAGCTACCAACTCTTTTT
CCGAAGGTAACTGGCTTCAGCAGAGCGCAGATACCAAATACTGTTCTTCTAGTGTAGCCGTAGTTAGG
CCACCACTTCAAGAACTCTGTAGCACCGCCTACATACCTCGCTCTGCTAATCCTGTTACCAGTGGCTG
CTGCCAGTGGCGATAAGTCGTGTCTTACCGGGTTGGACTCAAGACGATAGTTACCGGATAAGGCGCAG
CGGTCGGGCTGAACGGGGGGTTCGTGCACACAGCCCAGCTTGGAGCGAACGACCTACACCGAACTGAG
ATACCTACAGCGTGAGCTATGAGAAAGCGCCACGCTTCCCGAAGGGAGAAAGGCGGACAGGTATCCGG
TAAGCGGCAGGGTCGGAACAGGAGAGCGCACGAGGGAGCTTCCAGGGGGAAACGCCTGGTATCTTTAT
AGTCCTGTCGGGTTTCGCCACCTCTGACTTGAGCGTCGATTTTTGTGATGCTCGTCAGGGGGGCGGAG
CCTATGGAAAAACGCCAGCAACGCGGCCTTTT

**Map and nucleotide sequence of *CCR5*-targeting plasmid BB44_pmc.Donor$^{R5.TS}$**. Orange regions, sequences homologous to the human *CCR5* safe harbour locus; Magenta arrows, gRNA$^{CCR5}$ target site (TS); hPGK promoter, human phosphoglycerate kinase 1 gene (*PGK1*) regulatory sequences; mCherry, open reading frame of a monomeric derivative of DsRed fluorescent protein; bGH poly(A) signal, bovine growth hormone gene (*GH1*) polyadenylation signal; AmpR, *β-lactamase* ampicillin resistance gene.

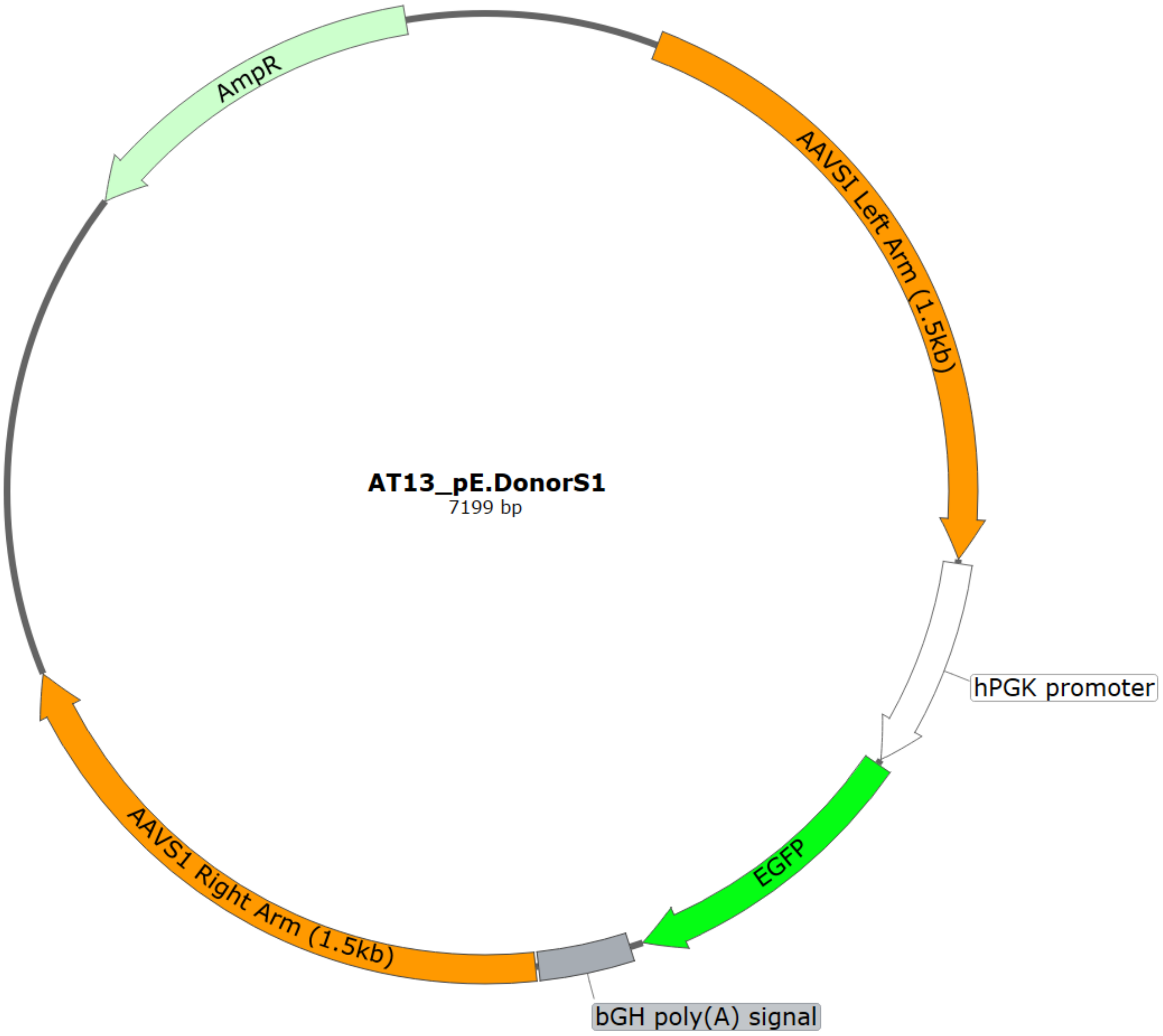


>AT13_pE.Donor[S1] (7199 bp)

CCTTTCGTCTTCAAGAATTGGAAACAGCTATGACCATGATTACGCCAAGCTCGAAATTACCCCTCACT
AAAGGGAACAAAGCTGGTACGAGGACAGGCTGGAGCCATGGGCATGGCTACTCAAGCTGATTTGATGG
AGTTGGACATGGCCATGGCTGGTGACCACGTCGTGGAATGCCTTCGAATTCAGCACCTGCACATGGGA
CGTCGACCTGAGGTAATTAGTACCGCGGCCTAATGTGAGTTAGCTCACTCATTAGGCACCCCAGGCTT
TACACTTTATGCTTCCGGCTCGTATGTTGTGTGGAATTGTGAGCGGATAACAATTTCACACAGGAAAC
AGCTATGACCATGATTACGCCAAGCGCGCAATTAACCCTCACTAAAGGGAACAAAAGCTGGAGCTCCA
CCGCGGTGGCGGCCCGCCGAGCTCGACCCCGCCGCGCCGCCGCCCGCCCGCGCCGTGCTGGACTCCAC
CAACGCCGACGGTATCAGCGCCCTGCACCAGGTCAGCGCCCCCCGCCCGGCGTCTCCCGGGGCCAGGT
CCACCCTCTGCTGCGCCACCTGGGGCATCCTCCTTCCCCGTTGCCAGTCTCGATCCGCCCCGTCGTTC
CTGGCCCTGGGCTTTGCCACCCTATGCTGACACCCCGTCCCAGTCCCCCTTACCATTCCCCTTCGACC
ACCCCACTTCCGAATTGGAGCCGCTTCAACTGGCCCTGGGCTTAGCCACTCTGTGCTGACCACTCTGC
CCCAGGCCTCCTTACCATTCCCCTTCGACCTACTCTCTTCCGCATTGGAGTCGCTTTAACTGGCCCTG
GCTTTGGCAGCCTGTGCTGACCCATGCAGTCCTCCTTACCATCCCTCCCTCGACTTCCCCTCTTCCGA
TGTTGAGCCCCTCCAGCCGGTCCTGGACTTTGTCTCCTTCCCTGCCCTGCCCTCTCCTGAACCTGAGC
CAGCTCCCATAGCTCAGTCTGGTCTATCTGCCTGGCCCTGGCCATTGTCACTTTGCGCTGCCCTCCTC
TCGCCCCCGAGTGCCCTTGCTGTGCCGCCGGAACTCTGCCCTCTAACGCTGCCGTCTCTCTCCTGAGT
CCGGACCACTTTGAGCTCTACTGGCTTCTGCGCCGCCTCTGGCCCACTGTTTCCCCTTCCCAGGCAGG
TCCTGCTTTCTCTGACCTGCATTCTCTCCCCTGGGCCTGTGCCGCTTTCTGTCTGCAGCTTGTGGCCT
GGGTCACCTCTACGGCTGGCCCAGATCCTTCCCTGCCGCCTCCTTCAGGTTCCGTCTTCCTCCACTCC

CTCTTCCCCTTGCTCTCTGCTGTGTTGCTGCCCAAGGATGCTCTTTCCGGAGCACTTCCTTCTCGGCG
CTGCACCACGTGATGTCCTCTGAGCGGATCCTCCCCGTGTCTGGGTCCTCTCCGGGCATCTCTCCTCC
CTCACCCAACCCCATGCCGTCTTCACTCGCTGGGTTCCCTTTTCCTTCTCCTTCTGGGGCCTGTGCCA
TCTCTCGTTTCTTAGGATGGCCTTCTCCGACGGATGTCTCCCTTGCGTCCCGCCTCCCCTTCTTGTAG
GCCTGCATCATCACCGTTTTTCTGGACAACCCCAAAGTACCCCGTCTCCCTGGCTTTAGCCACCTCTC
CATCCTCTTGCTTTCTTTGCCTGGACACCCCGTTCTCCTGTGGATTCGGGTCACCTCTCACTCCTTTC
ATTTGGGCAGCTCCCCTACCCCCCTTACCTCTCTAGTCTGTGCTAGCTCTTCCAGCCCCCTGTCATGG
CATCTTCCAGGGGTCCGAGAGCTCAGCTAGTCTTCTTCCTCCAACCCGGGCCCCTATGTCCACTTCAG
GACAGCATGTTTGCTGCCTCCAGGGATCCTGTGTCCCCGAGCTGGGACCACCTTATATTCCCAGGGCC
GGTTAATGTGGCTCTGGTTCTGGGTACTTTTATCTGTCCCCTCCACCCCACAGTGGGGCAAGCTTCCA
CGGGGTTGGGGTTGCGCCTTTTCCAAGGCAGCCCTGGGTTTGCGCAGGGACGCGGCTGCTCTGGGCGT
GGTTCCGGGAAACGCAGCGGCGCCGACCCTGGGTCTCGCACATTCTTCACGTCCGTTCGCAGCGTCAC
CCGGATCTTCGCCGCTACCCTTGTGGGCCCCCCGGCGACGCTTCCTGCTCCGCCCCTAAGTCGGGAAG
GTTCCTTGCGGTTCGCGGCGTGCCGGACGTGACAAACGGAAGCCGCACGTCTCACTAGTACCCTCGCA
GACGGACAGCGCCAGGGAGCAATGGCAGCGCGCCGACCGCGATGGGCTGTGGCCAATAGCGGCTGCTC
AGCAGGGCGCGCCGAGAGCAGCGGCCGGGAAGGGGCGGTGCGGGAGGCGGGGTGTGGGGCGGTAGTGT
GGGCCCTGTTCCTGCCCGCGCGGTGTTCCGCATTCTGCAAGCCTCCGGAGCGCACGTCGGCAGTCGGC
TCCCTCGTTGACCGAATCACCGACCTCTCTCCCCACCGGTCGCCACCATGGTGAGCAAGGGCGAGGAG
CTGTTCACCGGGGTGGTGCCCATCCTGGTCGAGCTGGACGGCGACGTAAACGGCCACAAGTTCAGCGT
GTCCGGCGAGGGCGAGGGCGATGCCACCTACGGCAAGCTGACCCTGAAGTTCATCTGCACCACCGGCA
AGCTGCCCGTGCCCTGGCCCACCCTCGTGACCACCCTGACCTACGGCGTGCAGTGCTTCAGCCGCTAC
CCCGACCACATGAAGCAGCACGACTTCTTCAAGTCCGCCATGCCCGAAGGCTACGTCCAGGAGCGCAC
CATCTTCTTCAAGGACGACGGCAACTACAAGACCCGCGCCGAGGTGAAGTTCGAGGGCGACACCCTGG
TGAACCGCATCGAGCTGAAGGGCATCGACTTCAAGGAGGACGGCAACATCCTGGGGCACAAGCTGGAG
TACAACTACAACAGCCACAACGTCTATATCATGGCCGACAAGCAGAAGAACGGCATCAAGGTGAACTT
CAAGATCCGCCACAACATCGAGGACGGCAGCGTGCAGCTCGCCGACCACTACCAGCAGAACACCCCCA
TCGGCGACGGCCCCGTGCTGCTGCCCGACAACCACTACCTGAGCACCCAGTCCGCCCTGAGCAAAGAC
CCCAACGAGAAGCGCGATCACATGGTCCTGCTGGAGTTCGTGACCGCCGCCGGGATCACTCTCGGCAT
GGACGAGCTGTACAAGTAAAGCGGCCGCGTCGAGTCTAGGATCAGCCTCGACTGTGCCTTCTAGTTGC
CAGCCATCTGTTGTTTGCCCCTCCCCCGTGCCTTCCTTGACCCTGGAAGGTGCCACTCCCACTGTCCT
TTCCTAATAAAATGAGGAAATTGCATCGCATTGTCTGAGTAGGTGTCATTCTATTCTGGGGGGTGGGG
TGGGGCAGGACAGCAAGGGGGAGGATTGGGAAGACAATAGCAGGCATGCTGGGGATGCGGTGGGCTCT
ATGGAAGCTTTACTAGGGACAGGATTGGTGACAGAAAAGCCCCATCCTTAGGCCTCCTCCTTCCTAGT
CTCCTGATATTGGGTCTAACCCCCACCTCCTGTTAGGCAGATTCCTTATCTGGTGACACACCCCCATT
TCCTGGAGCCATCTCTCTCCTTGCCAGAACCTCTAAGGTTTGCTTACGATGGAGCCAGAGAGGATCCT
GGGAGGGAGAGCTTGGCAGGGGGTGGGAGGGAAGGGGGGGATGCGTGACCTGCCCGGTTCTCAGTGGC
CACCCTGCGCTACCCTCTCCCAGAACCTGAGCTGCTCTGACGCGGCTGTCTGGTGCGTTTCACTGATC
CTGGTGCTGCAGCTTCCTTACACTTCCCAAGAGGAGAAGCAGTTTGGAAAAACAAAATCAGAATAAGT
TGGTCCTGAGTTCTAACTTTGGCTCTTCACCTTTCTAGTCCCCAATTTATATTGTTCCTCCGTGCGTC
AGTTTTACCTGTGAGATAAGGCCAGTAGCCAGCCCCGTCCTGGCAGGGCTGTGGTGAGGAGGGGGGTG
TCCGTGTGGAAAACTCCCTTTGTGAGAATGGTGCGTCCTAGGTGTTCACCAGGTCGTGGCCGCCTCTA
CTCCCTTTCTCTTTCTCCATCCTTCTTTCCTTAAAGAGTCCCCAGTGCTATCTGGGACATATTCCTCC
GCCCAGAGCAGGGTCCCGCTTCCCTAAGGCCCTGCTCTGGGCTTCTGGGTTTGAGTCCTTGGCAAGCC
CAGGAGAGGCGCTCAGGCTTCCCTGTCCCCCTTCCTCGTCCACCATCTCATGCCCCTGGCTCTCCTGC
CCCTTCCCTACAGGGGTTCCTGGCTCTGCTCTTCAGACTGAGCCCCGTTCCCCTGCATCCCCGTTCCC
CTGCATCCCCCTTCCCCTGCATCCCCCAGAGGCCCCAGGCCACCTACTTGGCCTGGACCCCACGAGAG
GCCACCCCAGCCCTGTCTACCAGGCTGCCTTTTGGGTGGATTCTCCTCCAACTGTGGGGTGACTGCTT
GGCAAACTCACTCTTCGGGGTATCCCAGGAGGCCTGGAGCATTGGGGTGGGCTGGGGTTCAGAGAGGA

GGGATTCCCTTCTCAGGTTACGTGGCCAAGAAGCAGGGGAGCTGGGTTTGGGTCAGGTCTGGGTGTGG
GGTGACCAGCTTATGCTGTTTGCCCAGGACAGCCTAGTTTTAGCACTGAAACCCTCAGTCCTAGGTGT
TCACCAGGTCGTGGCCGCCTCTACTCCCTTTCTCTTTCTCCATCCTTCTTTCCTTAAAGAGTCCCCAG
TGCTATCTGGGACATATTCCTCCGCCCAGAGCAGGGTCCCGCTTCCCTAAGGCCCTGCTCTGGGCTTC
TGGGTTTGAGTCCTTGGCAAGCCCAGGAGAGGCGCTCAGGCTTCCCTGTCCCCCTTCCTCGTCCACCA
TCTCATGCCCCTGGCTCTCCTGCCCCTTCCCTACAGGGGTTCCTGGCTCTGCTCTAAGGGCGAATTCG
ATCTGGCGCCATCGATACGCGTACGTCGCGACCGCGGACATGTACAGAGCTCGAGAAGTACTAGTGGC
CACGTGGGCCGTGCACCTTAAGCTTTTAAATAAGGAGGAATAACATATGACCATGATTACGCCAAGCT
CCAATTCGCCCTATAGTGAGTCGTATTACAATTCACTGGCCGTCGTTTTACTATGCGGTGTGAAATAC
CGCACAGATGCGTAAGGAGAAAATACCGCATCAGGCGCTCTTCCGCTTCCTCGCTCACTGACTCGCTG
CGCTCGGTCGTTCGGCTGCGGCGAGCGGTATCAGCTCACTCAAAGGCGGTAATACGGTTATCCACAGA
ATCAGGGGATAACGCAGGAAAGAACATGTGAGCAAAAGGCCAGCAAAAGGCCAGGAACCGTAAAAAGG
CCGCGTTGCTGGCGTTTTTCCATAGGCTCCGCCCCCCTGACGAGCATCACAAAAATCGACGCTCAAGT
CAGAGGTGGCGAAACCCGACAGGACTATAAAGATACCAGGCGTTTCCCCCTGGAAGCTCCCTCGTGCG
CTCTCCTGTTCCGACCCTGCCGCTTACCGGATACCTGTCCGCCTTTCTCCCTTCGGGAAGCGTGGCGC
TTTCTCATAGCTCACGCTGTAGGTATCTCAGTTCGGTGTAGGTCGTTCGCTCCAAGCTGGGCTGTGTG
CACGAACCCCCCGTTCAGCCCGACCGCTGCGCCTTATCCGGTAACTATCGTCTTGAGTCCAACCCGGT
AAGACACGACTTATCGCCACTGGCAGCAGCCACTGGTAACAGGATTAGCAGAGCGAGGTATGTAGGCG
GTGCTACAGAGTTCTTGAAGTGGTGGCCTAACTACGGCTACACTAGAAGGACAGTATTTGGTATCTGC
GCTCTGCTGAAGCCAGTTACCTTCGGAAAAAGAGTTGGTAGCTCTTGATCCGGCAAACAAACCACCGC
TGGTAGCGGTGGTTTTTTTGTTTGCAAGCAGCAGATTACGCGCAGAAAAAAAGGATCTCAAGAAGATC
CTTTGATCTTTTCTACGGGGTCTGACGCTCAGTGGAACGAAAACTCACGTTAAGGGATTTTGGTCATG
AGATTATCAAAAAGGATCTTCACCTAGATCCTTTTAAATTAAAAATGAAGTTTTAAATCAATCTAAAG
TATATATGAGTAAACTTGGTCTGACAGTTACCAATGCTTAATCAGTGAGGCACCTATCTCAGCGATCT
GTCTATTTCGTTCATCCATAGTTGCCTGACTCCCCGTCGTGTAGATAACTACGATACGGGAGGGCTTA
CCATCTGGCCCCAGTGCTGCAATGATACCGCGAGACCCACGCTCACCGGCTCCAGATTTATCAGCAAT
AAACCAGCCAGCCGGAAGGGCCGAGCGCAGAAGTGGTCCTGCAACTTTATCCGCCTCCATCCAGTCTA
TTAATTGTTGCCGGGAAGCTAGAGTAAGTAGTTCGCCAGTTAATAGTTTGCGCAACGTTGTTGCCATT
GCTGCAGGCATCGTGGTGTCACGCTCGTCGTTTGGTATGGCTTCATTCAGCTCCGGTTCCCAACGATC
AAGGCGAGTTACATGATCCCCCATGTTGTGCAAAAAAGCGGTTAGCTCCTTCGGTCCTCCGATCGTTG
TCAGAAGTAAGTTGGCCGCAGTGTTATCACTCATGGTTATGGCAGCACTGCATAATTCTCTTACTGTC
ATGCCATCCGTAAGATGCTTTTCTGTGACTGGTGAGTACTCAACCAAGTCATTCTGAGAATAGTGTAT
GCGGCGACCGAGTTGCTCTTGCCCGGCGTCAACACGGGATAATACCGCGCCACATAGCAGAACTTTAA
AAGTGCTCATCATTGGAAAACGTTCTTCGGGGCGAAAACTCTCAAGGATCTTACCGCTGTTGAGATCC
AGTTCGATGTAACCCACTCGTGCACCCAACTGATCTTCAGCATCTTTTACTTTCACCAGCGTTTCTGG
GTGAGCAAAAACAGGAAGGCAAAATGCCGCAAAAAAGGGAATAAGGGCGACACGGAAATGTTGAATAC
TCATACTCTTCCTTTTTCAATATTATTGAAGCATTTATCAGGGTTATTGTCTCATGAGCGGATACATA
TTTGAATGTATTTAGAAAAATAAACAAATAGGGGTTCCGCGCACATTTCCCCGAAAAGTGCCACCTGA
CGTCTAAGAAACCATTATTATCATGACATTAACCTATAAAAATAGGCGTATCACGAGGC

**Map and nucleotide sequence of *AAVS1*-targeting plasmid AT13_pE.Donor[S1]**. Orange regions, sequences homologous to the human *AAVS1* safe harbour locus; hPGK promoter, human phosphoglycerate kinase 1 gene (*PGK1*) regulatory sequences; EGFP, open reading frame of the enhanced green fluorescence protein reporter; bGH poly(A) signal, bovine growth hormone gene (*GH1*) polyadenylation signal; AmpR, *β-lactamase* ampicillin resistance gene.

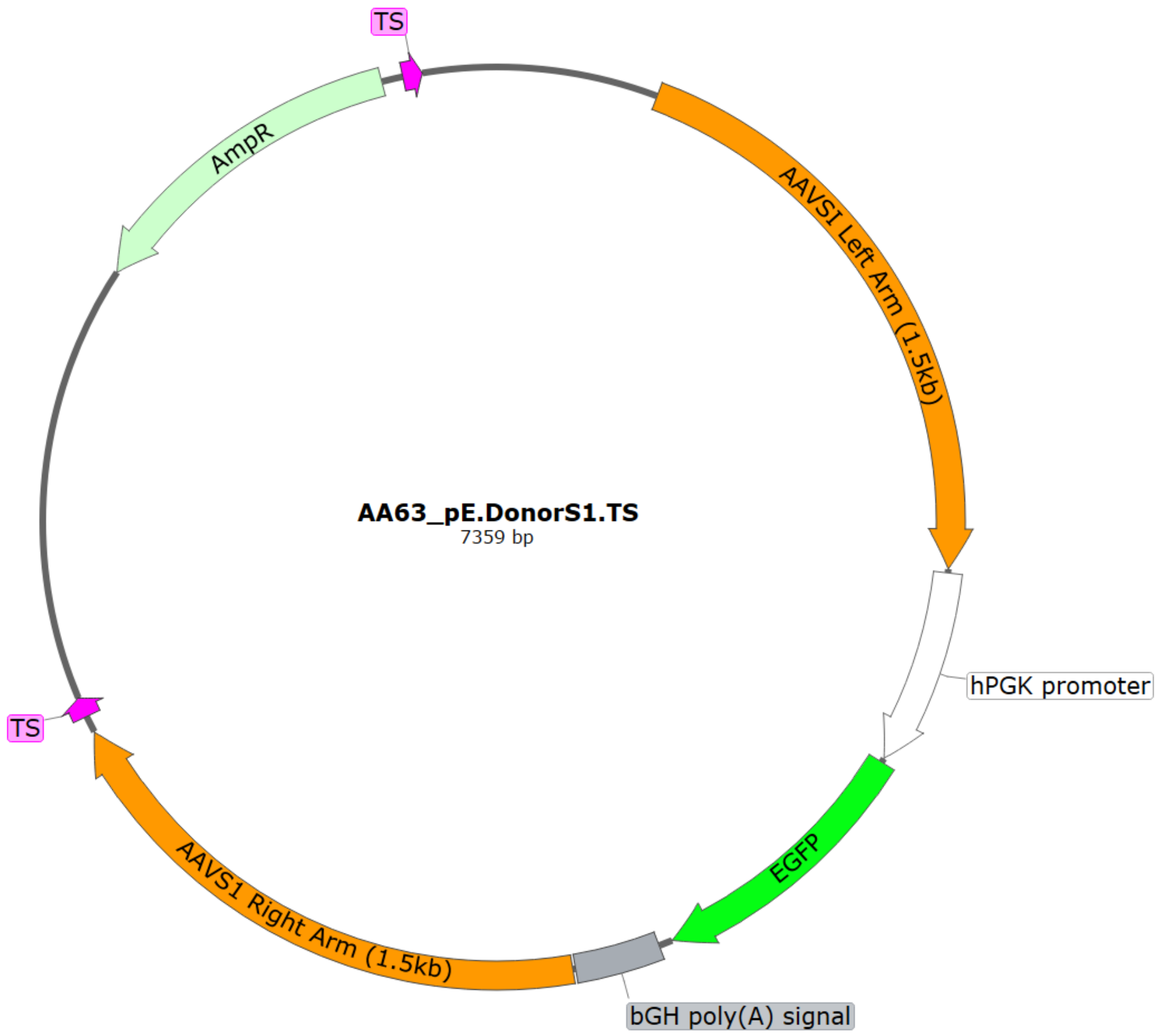


>AA63_pE.Donor$^{S1.TS}$ (7359 bp)

CCTTTCGTCTTCAAGAATTGGAAACAGCTATGACCATGATTACGCCAAGCTCGAAATTACCCCTCACT
AAAGGGAACAAAGCTGGTACGAGGACAGGCTGGAGCCATGGGCATGGCTACTCAAGCTGATTTGATGG
AGTTGGACATGGCCATGGCTGGTGACCACGTCGTGGAATGCCTTCGAATTCAGCACCTGCACATGGGA
CGTCGACCTGAGGTAATTAGTACCGCGGCCTAATGTGAGTTAGCTCACTCATTAGGCACCCCAGGCTT
TACACTTTATGCTTCCGGCTCGTATGTTGTGTGGAATTGTGAGCGGATAACAATTTCACACAGGAAAC
AGCTATGACCATGATTACGCCAAGCGCGCAATTAACCCTCACTAAAGGGAACAAAAGCTGGAGCTCCA
CCGCGGTGGCGGCCCGCCGAGCTCGACCCCGCCGCGCCGCCGCCCGCCCGCGCCGTGCTGGACTCCAC
CAACGCCGACGGTATCAGCGCCCTGCACCAGGTCAGCGCCCCCGCCCGGCGTCTCCCGGGGCCAGGT
CCACCCTCTGCTGCGCCACCTGGGGCATCCTCCTTCCCCGTTGCCAGTCTCGATCCGCCCCGTCGTTC
CTGGCCCTGGGCTTTGCCACCCTATGCTGACACCCCGTCCCAGTCCCCCTTACCATTCCCCTTCGACC
ACCCCACTTCCGAATTGGAGCCGCTTCAACTGGCCCTGGGCTTAGCCACTCTGTGCTGACCACTCTGC
CCCAGGCCTCCTTACCATTCCCCTTCGACCTACTCTCTTCCGCATTGGAGTCGCTTTAACTGGCCCTG
GCTTTGGCAGCCTGTGCTGACCCATGCAGTCCTCCTTACCATCCCTCCCTCGACTTCCCCTCTTCCGA
TGTTGAGCCCCTCCAGCCGGTCCTGGACTTTGTCTCCTTCCCTGCCCTGCCCTCTCCTGAACCTGAGC
CAGCTCCCATAGCTCAGTCTGGTCTATCTGCCTGGCCCTGGCCATTGTCACTTTGCGCTGCCCTCCTC
TCGCCCCCGAGTGCCCTTGCTGTGCCGCCGGAACTCTGCCCTCTAACGCTGCCGTCTCTCTCCTGAGT
CCGGACCACTTTGAGCTCTACTGGCTTCTGCGCCGCCTCTGGCCCACTGTTTCCCCTTCCCAGGCAGG
TCCTGCTTTCTCTGACCTGCATTCTCTCCCCTGGGCCTGTGCCGCTTTCTGTCTGCAGCTTGTGGCCT
GGGTCACCTCTACGGCTGGCCCAGATCCTTCCCTGCCGCCTCCTTCAGGTTCCGTCTTCCTCCACTCC

CTCTTCCCCTTGCTCTCTGCTGTGTTGCTGCCCAAGGATGCTCTTTCCGGAGCACTTCCTTCTCGGCG
CTGCACCACGTGATGTCCTCTGAGCGGATCCTCCCCGTGTCTGGGTCCTCTCCGGGCATCTCTCCTCC
CTCACCCAACCCCATGCCGTCTTCACTCGCTGGGTTCCCTTTTCCTTCTCCTTCTGGGGCCTGTGCCA
TCTCTCGTTTCTTAGGATGGCCTTCTCCGACGGATGTCTCCCTTGCGTCCCGCCTCCCCTTCTTGTAG
GCCTGCATCATCACCGTTTTTCTGGACAACCCCAAAGTACCCCGTCTCCCTGGCTTTAGCCACCTCTC
CATCCTCTTGCTTTCTTTGCCTGGACACCCCGTTCTCCTGTGGATTCGGGTCACCTCTCACTCCTTTC
ATTTGGGCAGCTCCCCTACCCCCCTTACCTCTCTAGTCTGTGCTAGCTCTTCCAGCCCCCTGTCATGG
CATCTTCCAGGGGTCCGAGAGCTCAGCTAGTCTTCTTCCTCCAACCCGGGCCCCTATGTCCACTTCAG
GACAGCATGTTTGCTGCCTCCAGGGATCCTGTGTCCCCGAGCTGGGACCACCTTATATTCCCAGGGCC
GGTTAATGTGGCTCTGGTTCTGGGTACTTTTATCTGTCCCCTCCACCCCACAGTGGGGCAAGCTTCCA
CGGGGTTGGGGTTGCGCCTTTTCCAAGGCAGCCCTGGGTTTGCGCAGGGACGCGGCTGCTCTGGGCGT
GGTTCCGGGAAACGCAGCGGCGCCGACCCTGGGTCTCGCACATTCTTCACGTCCGTTCGCAGCGTCAC
CCGGATCTTCGCCGCTACCCTTGTGGGCCCCCCGGCGACGCTTCCTGCTCCGCCCCTAAGTCGGGAAG
GTTCCTTGCGGTTCGCGGCGTGCCGGACGTGACAAACGGAAGCCGCACGTCTCACTAGTACCCTCGCA
GACGGACAGCGCCAGGGAGCAATGGCAGCGCGCCGACCGCGATGGGCTGTGGCCAATAGCGGCTGCTC
AGCAGGGCGCGCCGAGAGCAGCGGCCGGGAAGGGGCGGTGCGGGAGGCGGGGTGTGGGGCGGTAGTGT
GGGCCCTGTTCCTGCCCGCGCGGTGTTCCGCATTCTGCAAGCCTCCGGAGCGCACGTCGGCAGTCGGC
TCCCTCGTTGACCGAATCACCGACCTCTCTCCCCACCGGTCGCCACCATGGTGAGCAAGGGCGAGGAG
CTGTTCACCGGGGTGGTGCCCATCCTGGTCGAGCTGGACGGCGACGTAAACGGCCACAAGTTCAGCGT
GTCCGGCGAGGGCGAGGGCGATGCCACCTACGGCAAGCTGACCCTGAAGTTCATCTGCACCACCGGCA
AGCTGCCCGTGCCCTGGCCCACCCTCGTGACCACCCTGACCTACGGCGTGCAGTGCTTCAGCCGCTAC
CCCGACCACATGAAGCAGCACGACTTCTTCAAGTCCGCCATGCCCGAAGGCTACGTCCAGGAGCGCAC
CATCTTCTTCAAGGACGACGGCAACTACAAGACCCGCGCCGAGGTGAAGTTCGAGGGCGACACCCTGG
TGAACCGCATCGAGCTGAAGGGCATCGACTTCAAGGAGGACGGCAACATCCTGGGGCACAAGCTGGAG
TACAACTACAACAGCCACAACGTCTATATCATGGCCGACAAGCAGAAGAACGGCATCAAGGTGAACTT
CAAGATCCGCCACAACATCGAGGACGGCAGCGTGCAGCTCGCCGACCACTACCAGCAGAACACCCCCA
TCGGCGACGGCCCCGTGCTGCTGCCCGACAACCACTACCTGAGCACCCAGTCCGCCCTGAGCAAAGAC
CCCAACGAGAAGCGCGATCACATGGTCCTGCTGGAGTTCGTGACCGCCGCCGGGATCACTCTCGGCAT
GGACGAGCTGTACAAGTAAAGCGGCCGGCCGCGTCGAGTCTAGGATCAGCCTCGACTGTGCCTTCTAG
TTGCCAGCCATCTGTTGTTTGCCCCTCCCCCGTGCCTTCCTTGACCCTGGAAGGTGCCACTCCCACTG
TCCTTTCCTAATAAAATGAGGAAATTGCATCGCATTGTCTGAGTAGGTGTCATTCTATTCTGGGGGGT
GGGGTGGGGCAGGACAGCAAGGGGGAGGATTGGGAAGACAATAGCAGGCATGCTGGGGATGCGGTGGG
CTCTATGGAAGCTTTACTAGGGACAGGATTGGTGACAGAAAAGCCCCATCCTTAGGCCTCCTCCTTCC
TAGTCTCCTGATATTGGGTCTAACCCCCACCTCCTGTTAGGCAGATTCCTTATCTGGTGACACACCCC
CATTTCCTGGAGCCATCTCTCTCCTTGCCAGAACCTCTAAGGTTTGCTTACGATGGAGCCAGAGAGGA
TCCTGGGAGGGAGAGCTTGGCAGGGGGTGGGAGGGAAGGGGGGGATGCGTGACCTGCCCGGTTCTCAG
TGGCCACCCTGCGCTACCCTCTCCCAGAACCTGAGCTGCTCTGACGCGGCTGTCTGGTGCGTTTCACT
GATCCTGGTGCTGCAGCTTCCTTACACTTCCCAAGAGGAGAAGCAGTTTGGAAAAACAAAATCAGAAT
AAGTTGGTCCTGAGTTCTAACTTTGGCTCTTCACCTTTCTAGTCCCCAATTTATATTGTTCCTCCGTG
CGTCAGTTTTACCTGTGAGATAAGGCCAGTAGCCAGCCCCGTCCTGGCAGGGCTGTGGTGAGGAGGGG
GGTGTCCGTGTGGAAAACTCCCTTTGTGAGAATGGTGCGTCCTAGGTGTTCACCAGGTCGTGGCCGCC
TCTACTCCCTTTCTCTTTCTCCATCCTTCTTTCCTTAAAGAGTCCCCAGTGCTATCTGGGACATATTC
CTCCGCCCAGAGCAGGGTCCCGCTTCCCTAAGGCCCTGCTCTGGGCTTCTGGGTTTGAGTCCTTGGCA
AGCCCAGGAGAGGCGCTCAGGCTTCCCTGTCCCCCTTCCTCGTCCACCATCTCATGCCCCTGGCTCTC
CTGCCCCTTCCCTACAGGGGTTCCTGGCTCTGCTCTTCAGACTGAGCCCCGTTCCCCTGCATCCCCGT
TCCCCTGCATCCCCCTTCCCCTGCATCCCCCAGAGGCCCCAGGCCACCTACTTGGCCTGGACCCCACG
AGAGGCCACCCCAGCCCTGTCTACCAGGCTGCCTTTTGGGTGGATTCTCCTCCAACTGTGGGGTGACT
GCTTGGCAAACTCACTCTTCGGGGTATCCCAGGAGGCCTGGAGCATTGGGGTGGGCTGGGGTTCAGAG

AGGAGGGATTCCCTTCTCAGGTTACGTGGCCAAGAAGCAGGGGAGCTGGGTTTGGGTCAGGTCTGGGT
GTGGGGTGACCAGCTTATGCTGTTTGCCCAGGACAGCCTAGTTTTAGCACTGAAACCCTCAGTCCTAG
GTGTTCACCAGGTCGTGGCCGCCTCTACTCCCTTTCTCTTTCTCCATCCTTCTTTCCTTAAAGAGTCC
CCAGTGCTATCTGGGACATATTCCTCCGCCCAGAGCAGGGTCCCGCTTCCCTAAGGCCCTGCTCTGGG
CTTCTGGGTTTGAGTCCTTGGCAAGCCCAGGAGAGGCGCTCAGGCTTCCCTGTCCCCCTTCCTCGTCC
ACCATCTCATGCCCCTGGCTCTCCTGCCCCTTCCCTACAGGGGTTCCTGGCTCTGCTCTAAGGGCGAA
TTCGATATCAAGCTTATCGATACCGTCGACACGCGTCTGTCCCCTCCACCCCACAGTGGGGCCACTAG
GGACAGGATTGGTGACAGACTCGACTCGAAGATCTCGAGAAGTACTAGTGGCCACGTGGGCCGTGCAC
CTTAAGCTTTTAAATAAGGAGGAATAACATATGACCATGATTACGCCAAGCTCCAATTCGCCCTATAG
TGAGTCGTATTACAATTCACTGGCCGTCGTTTTACTATGCGGTGTGAAATACCGCACAGATGCGTAAG
GAGAAAATACCGCATCAGGCGCTCTTCCGCTTCCTCGCTCACTGACTCGCTGCGCTCGGTCGTTCGGC
TGCGGCGAGCGGTATCAGCTCACTCAAAGGCGGTAATACGGTTATCCACAGAATCAGGGGATAACGCA
GGAAAGAACATGTGAGCAAAAGGCCAGCAAAAGGCCAGGAACCGTAAAAAGGCCGCGTTGCTGGCGTT
TTTCCATAGGCTCCGCCCCCCTGACGAGCATCACAAAAATCGACGCTCAAGTCAGAGGTGGCGAAACC
CGACAGGACTATAAAGATACCAGGCGTTTCCCCCTGGAAGCTCCCTCGTGCGCTCTCCTGTTCCGACC
CTGCCGCTTACCGGATACCTGTCCGCCTTTCTCCCTTCGGGAAGCGTGGCGCTTTCTCATAGCTCACG
CTGTAGGTATCTCAGTTCGGTGTAGGTCGTTCGCTCCAAGCTGGGCTGTGTGCACGAACCCCCCGTTC
AGCCCGACCGCTGCGCCTTATCCGGTAACTATCGTCTTGAGTCCAACCCGGTAAGACACGACTTATCG
CCACTGGCAGCAGCCACTGGTAACAGGATTAGCAGAGCGAGGTATGTAGGCGGTGCTACAGAGTTCTT
GAAGTGGTGGCCTAACTACGGCTACACTAGAAGGACAGTATTTGGTATCTGCGCTCTGCTGAAGCCAG
TTACCTTCGGAAAAAGAGTTGGTAGCTCTTGATCCGGCAAACAAACCACCGCTGGTAGCGGTGGTTTT
TTTGTTTGCAAGCAGCAGATTACGCGCAGAAAAAAAGGATCTCAAGAAGATCCTTTGATCTTTTCTAC
GGGGTCTGACGCTCAGTGGAACGAAAACTCACGTTAAGGGATTTTGGTCATGAGATTATCAAAAAGGA
TCTTCACCTAGATCCTTTTAAATTAAAAATGAAGTTTTAAATCAATCTAAAGTATATATGAGTAAACT
TGGTCTGACAGTTACCAATGCTTAATCAGTGAGGCACCTATCTCAGCGATCTGTCTATTTCGTTCATC
CATAGTTGCCTGACTCCCCGTCGTGTAGATAACTACGATACGGGAGGGCTTACCATCTGGCCCCAGTG
CTGCAATGATACCGCGAGACCCACGCTCACCGGCTCCAGATTTATCAGCAATAAACCAGCCAGCCGGA
AGGGCCGAGCGCAGAAGTGGTCCTGCAACTTTATCCGCCTCCATCCAGTCTATTAATTGTTGCCGGGA
AGCTAGAGTAAGTAGTTCGCCAGTTAATAGTTTGCGCAACGTTGTTGCCATTGCTGCAGGCATCGTGG
TGTCACGCTCGTCGTTTGGTATGGCTTCATTCAGCTCCGGTTCCCAACGATCAAGGCGAGTTACATGA
TCCCCCATGTTGTGCAAAAAAGCGGTTAGCTCCTTCGGTCCTCCGATCGTTGTCAGAAGTAAGTTGGC
CGCAGTGTTATCACTCATGGTTATGGCAGCACTGCATAATTCTCTTACTGTCATGCCATCCGTAAGAT
GCTTTTCTGTGACTGGTGAGTACTCAACCAAGTCATTCTGAGAATAGTGTATGCGGCGACCGAGTTGC
TCTTGCCCGGCGTCAACACGGGATAATACCGCGCCACATAGCAGAACTTTAAAAGTGCTCATCATTGG
AAAACGTTCTTCGGGGCGAAAACTCTCAAGGATCTTACCGCTGTTGAGATCCAGTTCGATGTAACCCA
CTCGTGCACCCAACTGATCTTCAGCATCTTTTACTTTCACCAGCGTTTCTGGGTGAGCAAAAACAGGA
AGGCAAAATGCCGCAAAAAAGGGAATAAGGGCGACACGGAAATGTTGAATACTCATACTCTTCCTTTT
TCAATAATTCGATATCAAGCTTATCGATACCGTCGACACGCGTCTGTCCCCTCCACCCCACAGTGGGG
CCACTAGGGACAGGATTGGTGACAGACTCGACTCGAAGATCTCGAATTATTGAAGCATTTATCAGGGT
TATTGTCTCATGAGCGGATACATATTTGAATGTATTTAGAAAAATAAACAAATAGGGGTTCCGCGCAC
ATTTCCCCGAAAAGTGCCACCTGACGTCTAAGAAACCATTATTATCATGACATTAACCTATAAAAATA
GGCGTATCACGAGGC

**Map and nucleotide sequence of *AAVS1*-targeting plasmid AA63_pE.Donor$^{S1.TS}$**. Orange regions, sequences homologous to the human *AAVS1* safe harbour locus; Magenta arrows, gRNA$^{S1}$ target site (TS); hPGK promoter, human phosphoglycerate kinase 1 gene (*PGK1*) regulatory sequences; EGFP, open reading frame of the enhanced green fluorescence protein reporter; bGH poly(A) signal, bovine growth hormone gene (*GH1*) polyadenylation signal; AmpR, *β-lactamase* ampicillin resistance gene.

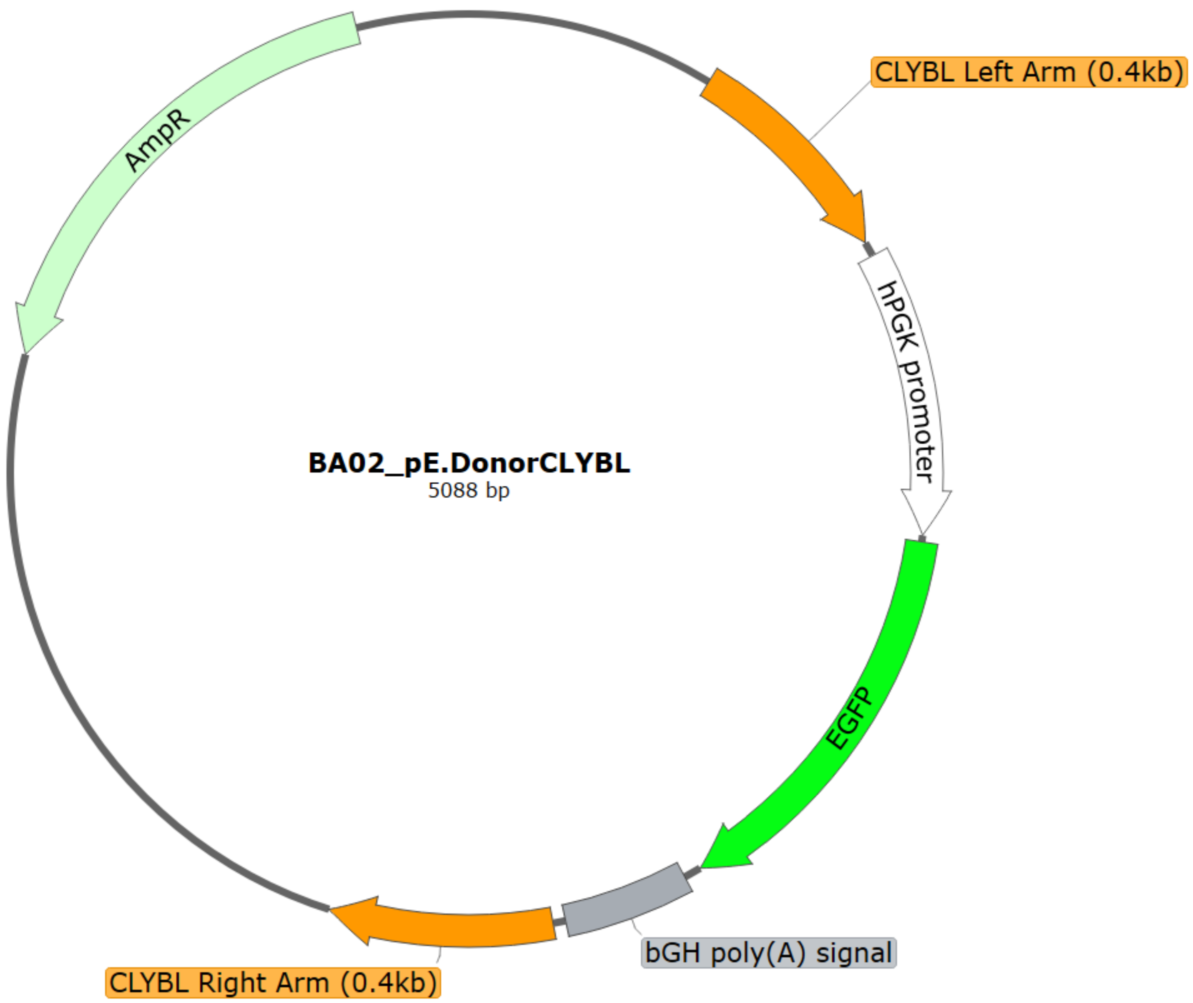


>BA02_pE.Donor$^{CLYBL}$ (5088 bp)

```
TCGCGCGTTTCGGTGATGACGGTGAAAACCTCTGACACATGCAGCTCCCGGAGACGGTCACAGCTTGT
CTGTAAGCGGATGCCGGGAGCAGACAAGCCCGTCAGGGCGCGTCAGCGGGTGTTGGCGGGTGTCGGGG
CTGGCTTAACTATGCGGCATCAGAGCAGATTGTACTGAGAGTGCACCATATGCGGTGTGAAATACCGC
ACAGATGCGTAAGGAGAAAATACCGCATCAGGCGCCATTCGCCATTCAGGCTGCGCAACTGTTGGGAA
GGGCGATCGGTGCGGGCCTCTTCGCTATTACGCCAGCTGGCGAAAGGGGGATGTGCTGCAAGGCGATT
AAGTTGGGTAACGCCAGGGTTTTCCCAGTCACGACGTTGTAAAACGACGGCCAGTGAATTCGAGCTCG
GTACCTCGCGAATGCATCTAGATGACGACCGCCGGAAATATTCAGAATGCTTTAATGGAGTAAGAAGG
GCCACTGCTTAGAGTGGGGCTGAAGAGAAAAGAAAGAAGGAGACCCTGCGGAAGCGGCAATACCAGGA
AGGCAGTTGTATGCACATCATGCAGACCAGGAGTAGCCTAGGGGTAGGGGACATGCCTGGGCTGGACA
GTGGAATTTGTGGGAAATCAGATTTCCCTGAGCAAAAACCAGTGGGCAGCAGCCAAACTGGTGAGCAA
GAACTGGCTGTGGTGGCTGGGCCCGAATTGGGAGCTACACCTGCCCGGGCAGGTGTAGCTGCGAGGGA
GGTCTCTGCCAACAACTCAGCTGTGCCGTGCCTGCTGAACGGCCTCACCTTGGCCTCTGGCTCTGTTG
CAGCTGTGTGGGGCTCGTGCTCCTACATACCACGCGTGTTCGCGTGTTAGCTTCCACGGGGTTGGGGT
TGCGCCTTTTCCAAGGCAGCCCTGGGTTTGCGCAGGGACGCGGCTGCTCTGGGCGTGGTTCCGGGAAA
CGCAGCGGCGCCGACCCTGGGTCTCGCACATTCTTCACGTCCGTTCGCAGCGTCACCCGGATCTTCGC
CGCTACCCTTGTGGGCCCCCCGGCGACGCTTCCTGCTCCGCCCCTAAGTCGGGAAGGTTCCTTGCGGT
TCGCGGCGTGCCGGACGTGACAAACGGAAGCCGCACGTCTCACTAGTACCCTCGCAGACGGACAGCGC
CAGGGAGCAATGGCAGCGCGCCGACCGCGATGGGCTGTGGCCAATAGCGGCTGCTCAGCAGGGCGCGC
CGAGAGCAGCGGCCGGGAAGGGGCGGTGCGGGAGGCGGGGTGTGGGGCGGTAGTGTGGGCCCTGTTCC
TGCCCGCGCGGTGTTCCGCATTCTGCAAGCCTCCGGAGCGCACGTCGGCAGTCGGCTCCCTCGTTGAC
```

CGAATCACCGACCTCTCTCCCCACCGGTCGCCACCATGGTGAGCAAGGGCGAGGAGCTGTTCACCGGG
GTGGTGCCCATCCTGGTCGAGCTGGACGGCGACGTAAACGGCCACAAGTTCAGCGTGTCCGGCGAGGG
CGAGGGCGATGCCACCTACGGCAAGCTGACCCTGAAGTTCATCTGCACCACCGGCAAGCTGCCCGTGC
CCTGGCCCACCCTCGTGACCACCCTGACCTACGGCGTGCAGTGCTTCAGCCGCTACCCCGACCACATG
AAGCAGCACGACTTCTTCAAGTCCGCCATGCCCGAAGGCTACGTCCAGGAGCGCACCATCTTCTTCAA
GGACGACGGCAACTACAAGACCCGCGCCGAGGTGAAGTTCGAGGGCGACACCCTGGTGAACCGCATCG
AGCTGAAGGGCATCGACTTCAAGGAGGACGGCAACATCCTGGGGCACAAGCTGGAGTACAACTACAAC
AGCCACAACGTCTATATCATGGCCGACAAGCAGAAGAACGGCATCAAGGTGAACTTCAAGATCCGCCA
CAACATCGAGGACGGCAGCGTGCAGCTCGCCGACCACTACCAGCAGAACACCCCCATCGGCGACGGCC
CCGTGCTGCTGCCCGACAACCACTACCTGAGCACCCAGTCCGCCCTGAGCAAAGACCCCAACGAGAAG
CGCGATCACATGGTCCTGCTGGAGTTCGTGACCGCCGCCGGGATCACTCTCGGCATGGACGAGCTGTA
CAAGTAAAGCGGCCGCGTCGAGTCTAGGATCAGCCTCGACTGTGCCTTCTAGTTGCCAGCCATCTGTT
GTTTGCCCCTCCCCCGTGCCTTCCTTGACCCTGGAAGGTGCCACTCCCACTGTCCTTTCCTAATAAAA
TGAGGAAATTGCATCGCATTGTCTGAGTAGGTGTCATTCTATTCTGGGGGGTGGGGTGGGGCAGGACA
GCAAGGGGGAGGATTGGGAAGACAATAGCAGGCATGCTGGGGATGCGGTGGGCTCTATGGAAGCTAAC
AAGCTAACAAGCTTGTTATCCTGTGGTAGAGTTTTACCGTGGACCAGAGCCATCTAGAGGACATTGAG
TTACCCACATACTTTCTGTGGGAGAGGCCAAGGAGTCAGGGGGGGAGGCCGGGACCTGAAGATCAACT
CTTTACTGCAGAGATCCCTCCCTCCCTCTCTCTGTCCCATGGATCCAGAGGGAAGTTGAGGACAGTTT
CATGCCACATGCTTGACCCGACTCAACTGAGTAACTGTGGCCACTGAGAAGACTCTGTCCTTGCAAAT
AACTCTCCTTAGTGCCCATATTCTGCAGAATGTTTGAGTGGCACGAGTACTTCATTCGTCTAGAAATA
TTTCATGGTAGTCAGTTGCTAATAACAGGATATCTGCAGGTAGTGTGTTCTCAGGAGGCACACCTGAA
GCCTCAGTTTGGCCGCGTCGTATCGGATCCCGGGCCCGTCGACTGCAGAGGCCTGCATGCAAGCTTGG
CGTAATCATGGTCATAGCTGTTTCCTGTGTGAAATTGTTATCCGCTCACAATTCCACACAACATACGA
GCCGGAAGCATAAAGTGTAAAGCCTGGGGTGCCTAATGAGTGAGCTAACTCACATTAATTGCGTTGCG
CTCACTGCCCGCTTTCCAGTCGGGAAACCTGTCGTGCCAGCTGCATTAATGAATCGGCCAACGCGCGG
GGAGAGGCGGTTTGCGTATTGGGCGCTCTTCCGCTTCCTCGCTCACTGACTCGCTGCGCTCGGTCGTT
CGGCTGCGGCGAGCGGTATCAGCTCACTCAAAGGCGGTAATACGGTTATCCACAGAATCAGGGGATAA
CGCAGGAAAGAACATGTGAGCAAAAGGCCAGCAAAAGGCCAGGAACCGTAAAAAGGCCGCGTTGCTGG
CGTTTTTCCATAGGCTCCGCCCCCCTGACGAGCATCACAAAAATCGACGCTCAAGTCAGAGGTGGCGA
AACCCGACAGGACTATAAAGATACCAGGCGTTTCCCCCTGGAAGCTCCCTCGTGCGCTCTCCTGTTCC
GACCCTGCCGCTTACCGGATACCTGTCCGCCTTTCTCCCTTCGGGAAGCGTGGCGCTTTCTCATAGCT
CACGCTGTAGGTATCTCAGTTCGGTGTAGGTCGTTCGCTCCAAGCTGGGCTGTGTGCACGAACCCCCC
GTTCAGCCCGACCGCTGCGCCTTATCCGGTAACTATCGTCTTGAGTCCAACCCGGTAAGACACGACTT
ATCGCCACTGGCAGCAGCCACTGGTAACAGGATTAGCAGAGCGAGGTATGTAGGCGGTGCTACAGAGT
TCTTGAAGTGGTGGCCTAACTACGGCTACACTAGAAGAACAGTATTTGGTATCTGCGCTCTGCTGAAG
CCAGTTACCTTCGGAAAAAGAGTTGGTAGCTCTTGATCCGGCAAACAAACCACCGCTGGTAGCGGTGG
TTTTTTTGTTTGCAAGCAGCAGATTACGCGCAGAAAAAAAGGATCTCAAGAAGATCCTTTGATCTTTT
CTACGGGGTCTGACGCTCAGTGGAACGAAAACTCACGTTAAGGGATTTTGGTCATGAGATTATCAAAA
AGGATCTTCACCTAGATCCTTTTAAATTAAAAATGAAGTTTTAAATCAATCTAAAGTATATATGAGTA
AACTTGGTCTGACAGTTACCAATGCTTAATCAGTGAGGCACCTATCTCAGCGATCTGTCTATTTCGTT
CATCCATAGTTGCCTGACTCCCCGTCGTGTAGATAACTACGATACGGGAGGGCTTACCATCTGGCCCC
AGTGCTGCAATGATACCGCGAGACCCACGCTCACCGGCTCCAGATTTATCAGCAATAAACCAGCCAGC
CGGAAGGGCCGAGCGCAGAAGTGGTCCTGCAACTTTATCCGCCTCCATCCAGTCTATTAATTGTTGCC
GGGAAGCTAGAGTAAGTAGTTCGCCAGTTAATAGTTTGCGCAACGTTGTTGCCATTGCTACAGGCATC
GTGGTGTCACGCTCGTCGTTTGGTATGGCTTCATTCAGCTCCGGTTCCCAACGATCAAGGCGAGTTAC
ATGATCCCCCATGTTGTGCAAAAAAGCGGTTAGCTCCTTCGGTCCTCCGATCGTTGTCAGAAGTAAGT
TGGCCGCAGTGTTATCACTCATGGTTATGGCAGCACTGCATAATTCTCTTACTGTCATGCCATCCGTA
AGATGCTTTTCTGTGACTGGTGAGTACTCAACCAAGTCATTCTGAGAATAGTGTATGCGGCGACCGAG

TTGCTCTTGCCCGGCGTCAATACGGGATAATACCGCGCCACATAGCAGAACTTTAAAAGTGCTCATCA
TTGGAAAACGTTCTTCGGGGCGAAAACTCTCAAGGATCTTACCGCTGTTGAGATCCAGTTCGATGTAA
CCCACTCGTGCACCCAACTGATCTTCAGCATCTTTTACTTTCACCAGCGTTTCTGGGTGAGCAAAAAC
AGGAAGGCAAAATGCCGCAAAAAAGGGAATAAGGGCGACACGGAAATGTTGAATACTCATACTCTTCC
TTTTTCAATATTATTGAAGCATTTATCAGGGTTATTGTCTCATGAGCGGATACATATTTGAATGTATT
TAGAAAAATAAACAAATAGGGGTTCCGCGCACATTTCCCCGAAAAGTGCCACCTGACGTCTAAGAAAC
CATTATTATCATGACATTAACCTATAAAAATAGGCGTATCACGAGGCCCTTTCGTC

**Map and nucleotide sequence of *CLYBL*-targeting plasmid BA02_pE.Donor$^{CLYBL}$.** Orange regions, sequences identical to the human *CLYBL* safe harbour locus; hPGK promoter, human phosphoglycerate kinase 1 gene (*PGK1*) regulatory sequences; EGFP, open reading frame of the enhanced green fluorescence protein gene; bGH poly(A) signal, bovine growth hormone gene (*GH1*) polyadenylation signal; AmpR, *β-lactamase* ampicillin resistance gene.

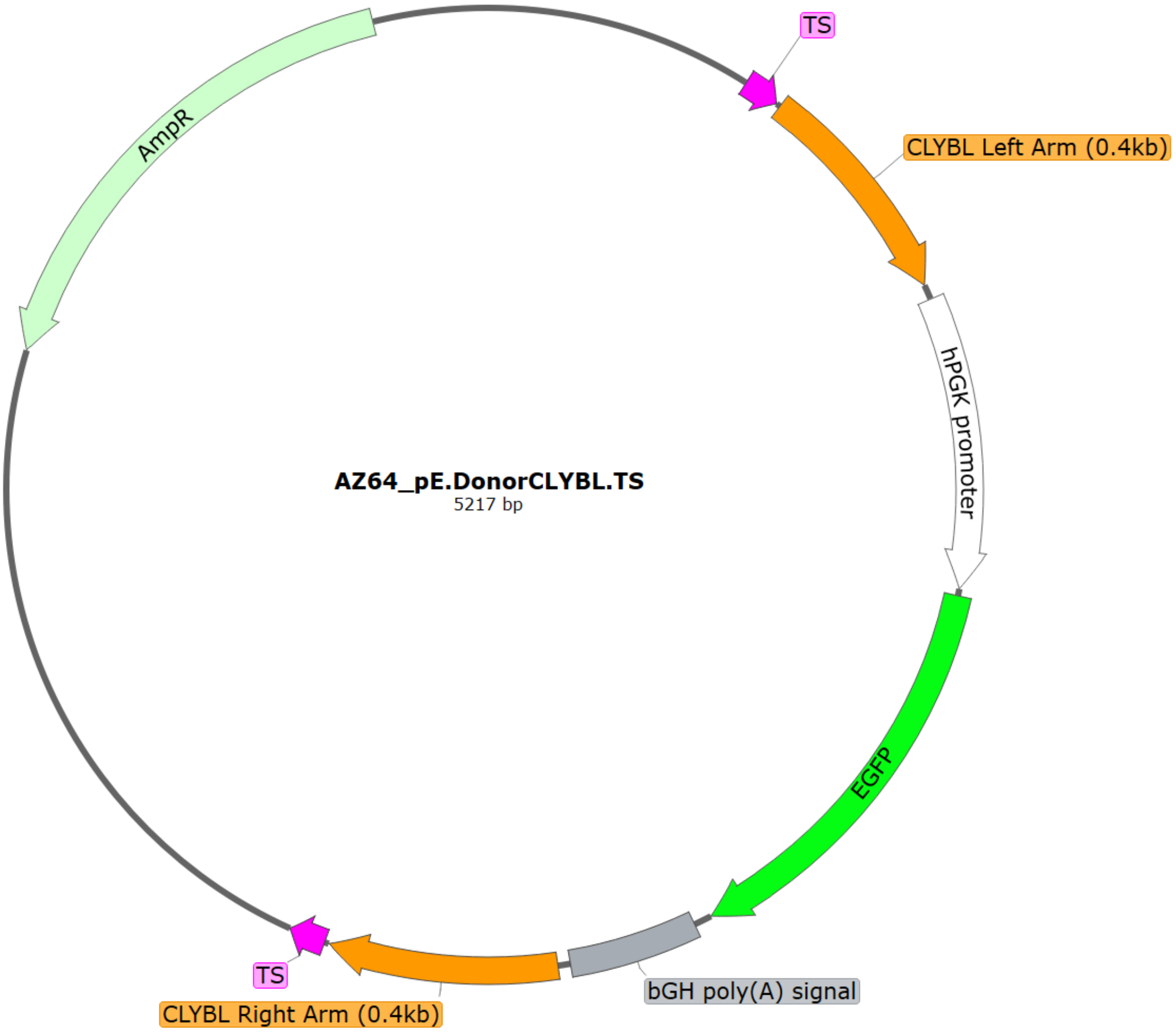


>AZ64_pE.Donor$^{CLYBL.TS}$ (5217 bp)
TCGCGCGTTTCGGTGATGACGGTGAAAACCTCTGACACATGCAGCTCCCGGAGACGGTCACAGCTTGT
CTGTAAGCGGATGCCGGGAGCAGACAAGCCCGTCAGGGCGCGTCAGCGGGTGTTGGCGGGTGTCGGGG
CTGGCTTAACTATGCGGCATCAGAGCAGATTGTACTGAGAGTGCACCATATGCGGTGTGAAATACCGC

ACAGATGCGTAAGGAGAAAATACCGCATCAGGCGCCATTCGCCATTCAGGCTGCGCAACTGTTGGGAA
GGGCGATCGGTGCGGGCCTCTTCGCTATTACGCCAGCTGGCGAAAGGGGGATGTGCTGCAAGGCGATT
AAGTTGGGTAACGCCAGGGTTTTCCCAGTCACGACGTTGTAAAACGACGGCCAGTGAATTCGAGCTCG
GTACCTCGCGAATGCATCTAGATATCGGATCCCGGGCCCGTCGACTGCAGAGGCGGCCGCCAGCTGT
GTGGGGCTCGTGCTCCTACATACCGTTATCCTGTGGTAGAGTTTTACCGTGGACCAGATTTAAATATT
CAGAATGCTTTAATGGAGTAAGAAGGGCCACTGCTTAGAGTGGGGCTGAAGAGAAAGAAAGAAGGAG
ACCCTGCGGAAGCGGCAATACCAGGAAGGCAGTTGTATGCACATCATGCAGACCAGGAGTAGCCTAGG
GGTAGGGGACATGCCTGGGCTGGACAGTGGAATTTGTGGGAAATCAGATTTCCCTGAGCAAAAACCAG
TGGGCAGCAGCCAAACTGGTGAGCAAGAACTGGCTGTGGTGGCTGGGCCCGAATTGGGAGCTACACCT
GCCCGGGCAGGTGTAGCTGCGAGGGAGGTCTCTGCCAACAACTCAGCTGTGCCGTGCCTGCTGAACGG
CCTCACCTTGGCCTCTGGCTCTGTTGCAGCTGTGTGGGGCTCGTGCTCCTACATACCACGCGTGTTCG
CGTGTTAGCTTCCACGGGGTTGGGGTTGCGCCTTTTCCAAGGCAGCCCTGGGTTTGCGCAGGGACGCG
GCTGCTCTGGGCGTGGTTCCGGGAAACGCAGCGGCGCCGACCCTGGGTCTCGCACATTCTTCACGTCC
GTTCGCAGCGTCACCCGGATCTTCGCCGCTACCCTTGTGGGCCCCCCGGCGACGCTTCCTGCTCCGCC
CCTAAGTCGGGAAGGTTCCTTGCGGTTCGCGGCGTGCCGGACGTGACAAACGGAAGCCGCACGTCTCA
CTAGTACCCTCGCAGACGGACAGCGCCAGGGAGCAATGGCAGCGCGCCGACCGCGATGGGCTGTGGCC
AATAGCGGCTGCTCAGCAGGGCGCGCCGAGAGCAGCGGCCGGGAAGGGGCGGTGCGGGAGGCGGGGTG
TGGGGCGGTAGTGTGGGCCCTGTTCCTGCCCGCGCGGTGTTCCGCATTCTGCAAGCCTCCGGAGCGCA
CGTCGGCAGTCGGCTCCCTCGTTGACCGAATCACCGACCTCTCTCCCCACCGGTCGCCACCATGGTGA
GCAAGGGCGAGGAGCTGTTCACCGGGGTGGTGCCCATCCTGGTCGAGCTGGACGGCGACGTAAACGGC
CACAAGTTCAGCGTGTCCGGCGAGGGCGAGGGCGATGCCACCTACGGCAAGCTGACCCTGAAGTTCAT
CTGCACCACCGGCAAGCTGCCCGTGCCCTGGCCCACCCTCGTGACCACCCTGACCTACGGCGTGCAGT
GCTTCAGCCGCTACCCCGACCACATGAAGCAGCACGACTTCTTCAAGTCCGCCATGCCCGAAGGCTAC
GTCCAGGAGCGCACCATCTTCTTCAAGGACGACGGCAACTACAAGACCCGCGCCGAGGTGAAGTTCGA
GGGCGACACCCTGGTGAACCGCATCGAGCTGAAGGGCATCGACTTCAAGGAGGACGGCAACATCCTGG
GGCACAAGCTGGAGTACAACTACAACAGCCACAACGTCTATATCATGGCCGACAAGCAGAAGAACGGC
ATCAAGGTGAACTTCAAGATCCGCCACAACATCGAGGACGGCAGCGTGCAGCTCGCCGACCACTACCA
GCAGAACACCCCCATCGGCGACGGCCCCGTGCTGCTGCCCGACAACCACTACCTGAGCACCCAGTCCG
CCCTGAGCAAAGACCCCAACGAGAAGCGCGATCACATGGTCCTGCTGGAGTTCGTGACCGCCGCCGGG
ATCACTCTCGGCATGGACGAGCTGTACAAGTAAAGCGGCCGCGTCGAGTCTAGGATCAGCCTCGACTG
TGCCTTCTAGTTGCCAGCCATCTGTTGTTTGCCCCTCCCCCGTGCCTTCCTTGACCCTGGAAGGTGCC
ACTCCCACTGTCCTTTCCTAATAAAATGAGGAAATTGCATCGCATTGTCTGAGTAGGTGTCATTCTAT
TCTGGGGGGTGGGGTGGGGCAGGACAGCAAGGGGGAGGATTGGGAAGACAATAGCAGGCATGCTGGGG
ATGCGGTGGGCTCTATGGAAGCTAACAAGCTAACAAGCTTGTTATCCTGTGGTAGAGTTTTACCGTGG
ACCAGAGCCATCTAGAGGACATTGAGTTACCCACATACTTTCTGTGGGAGAGGCCAAGGAGTCAGGGG
GGGAGGCCGGGACCTGAAGATCAACTCTTTACTGCAGAGATCCCTCCCTCCCTCTCTCTGTCCCATGG
ATCCAGAGGGAAGTTGAGGACAGTTTCATGCCACATGCTTGACCCGACTCAACTGAGTAACTGTGGCC
ACTGAGAAGACTCTGTCCTTGCAAATAACTCTCCTTAGTGCCCATATTCTGCAGAATGTTTGAGTGGC
ACGAGTACTTCATTCGTCTAGAAATATTTCATGGTAGTCAGTTGCTAATAACAGGATATCTGCAGGTA
GTGTGTTCTCAGGAGGCACACCTGAAGCCTCAGTTTAAACCAGCTGTGTGGGGCTCGTGCTCCTACAT
ACCGTTATCCTGTGGTAGAGTTTTACCGTGGACCAGCGCCGGCGCCTGCATGCAAGCTTGGCGTAATC
ATGGTCATAGCTGTTTCCTGTGTGAAATTGTTATCCGCTCACAATTCCACACAACATACGAGCCGGAA
GCATAAAGTGTAAAGCCTGGGGTGCCTAATGAGTGAGCTAACTCACATTAATTGCGTTGCGCTCACTG
CCCGCTTTCCAGTCGGGAAACCTGTCGTGCCAGCTGCATTAATGAATCGGCCAACGCGCGGGGAGAGG
CGGTTTGCGTATTGGGCGCTCTTCCGCTTCCTCGCTCACTGACTCGCTGCGCTCGGTCGTTCGGCTGC
GGCGAGCGGTATCAGCTCACTCAAAGGCGGTAATACGGTTATCCACAGAATCAGGGGATAACGCAGGA
AAGAACATGTGAGCAAAAGGCCAGCAAAAGGCCAGGAACCGTAAAAAGGCCGCGTTGCTGGCGTTTTT
CCATAGGCTCCGCCCCCCTGACGAGCATCACAAAAATCGACGCTCAAGTCAGAGGTGGCGAAACCCGA

```
CAGGACTATAAAGATACCAGGCGTTTCCCCCTGGAAGCTCCCTCGTGCGCTCTCCTGTTCCGACCCTG
CCGCTTACCGGATACCTGTCCGCCTTTCTCCCTTCGGGAAGCGTGGCGCTTTCTCATAGCTCACGCTG
TAGGTATCTCAGTTCGGTGTAGGTCGTTCGCTCCAAGCTGGGCTGTGTGCACGAACCCCCCGTTCAGC
CCGACCGCTGCGCCTTATCCGGTAACTATCGTCTTGAGTCCAACCCGGTAAGACACGACTTATCGCCA
CTGGCAGCAGCCACTGGTAACAGGATTAGCAGAGCGAGGTATGTAGGCGGTGCTACAGAGTTCTTGAA
GTGGTGGCCTAACTACGGCTACACTAGAAGAACAGTATTTGGTATCTGCGCTCTGCTGAAGCCAGTTA
CCTTCGGAAAAAGAGTTGGTAGCTCTTGATCCGGCAAACAAACCACCGCTGGTAGCGGTGGTTTTTTT
GTTTGCAAGCAGCAGATTACGCGCAGAAAAAAAGGATCTCAAGAAGATCCTTTGATCTTTTCTACGGG
GTCTGACGCTCAGTGGAACGAAAACTCACGTTAAGGGATTTTGGTCATGAGATTATCAAAAAGGATCT
TCACCTAGATCCTTTTAAATTAAAAATGAAGTTTTAAATCAATCTAAAGTATATATGAGTAAACTTGG
TCTGACAGTTACCAATGCTTAATCAGTGAGGCACCTATCTCAGCGATCTGTCTATTTCGTTCATCCAT
AGTTGCCTGACTCCCCGTCGTGTAGATAACTACGATACGGGAGGGCTTACCATCTGGCCCCAGTGCTG
CAATGATACCGCGAGACCCACGCTCACCGGCTCCAGATTTATCAGCAATAAACCAGCCAGCCGGAAGG
GCCGAGCGCAGAAGTGGTCCTGCAACTTTATCCGCCTCCATCCAGTCTATTAATTGTTGCCGGGAAGC
TAGAGTAAGTAGTTCGCCAGTTAATAGTTTGCGCAACGTTGTTGCCATTGCTACAGGCATCGTGGTGT
CACGCTCGTCGTTTGGTATGGCTTCATTCAGCTCCGGTTCCCAACGATCAAGGCGAGTTACATGATCC
CCCATGTTGTGCAAAAAAGCGGTTAGCTCCTTCGGTCCTCCGATCGTTGTCAGAAGTAAGTTGGCCGC
AGTGTTATCACTCATGGTTATGGCAGCACTGCATAATTCTCTTACTGTCATGCCATCCGTAAGATGCT
TTTCTGTGACTGGTGAGTACTCAACCAAGTCATTCTGAGAATAGTGTATGCGGCGACCGAGTTGCTCT
TGCCCGGCGTCAATACGGGATAATACCGCGCCACATAGCAGAACTTTAAAAGTGCTCATCATTGGAAA
ACGTTCTTCGGGGCGAAAACTCTCAAGGATCTTACCGCTGTTGAGATCCAGTTCGATGTAACCCACTC
GTGCACCCAACTGATCTTCAGCATCTTTTACTTTCACCAGCGTTTCTGGGTGAGCAAAAACAGGAAGG
CAAAATGCCGCAAAAAAGGGAATAAGGGCGACACGGAAATGTTGAATACTCATACTCTTCCTTTTTCA
ATATTATTGAAGCATTTATCAGGGTTATTGTCTCATGAGCGGATACATATTTGAATGTATTTAGAAAA
ATAAACAAATAGGGGTTCCGCGCACATTTCCCCGAAAAGTGCCACCTGACGTCTAAGAAACCATTATT
ATCATGACATTAACCTATAAAAATAGGCGTATCACGAGGCCCTTTCGTC
```

**Map and nucleotide sequence of *CLYBL*-targeting plasmid AZ64_pE.Donor$^{CLYBL.TS}$**. Orange regions, sequences homologous to the human *CLYBL* safe harbour locus; Magenta arrows, gRNA$^{CLYBL}$ target site (TS); hPGK promoter, human phosphoglycerate kinase 1 gene (*PGK1*) regulatory sequences; EGFP, open reading frame of enhanced green fluorescence protein gene; bGH poly(A) signal, bovine growth hormone gene (*GH1*) polyadenylation signal; AmpR, *β-lactamase* ampicillin resistance gene.

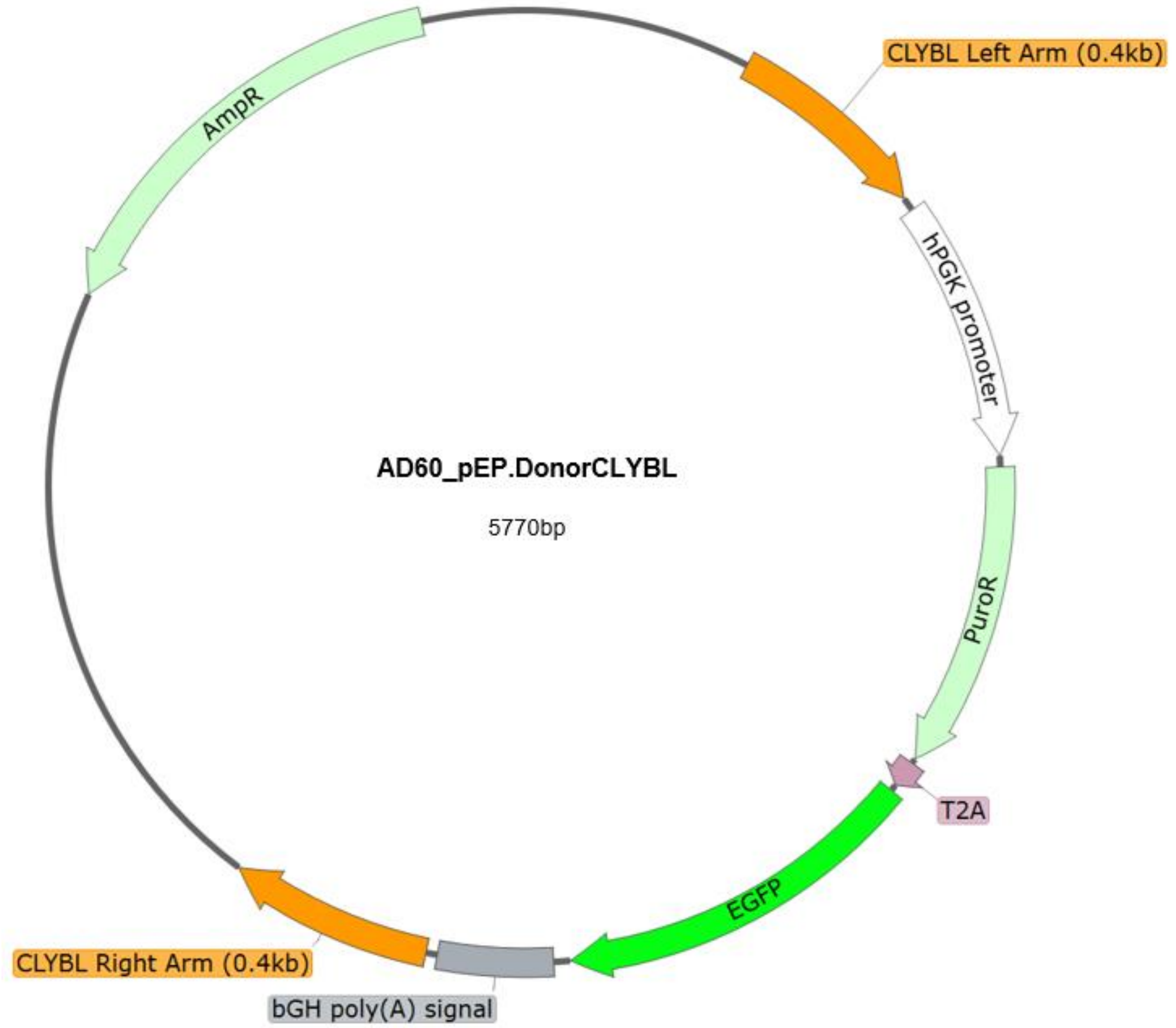


>AD60_pEP.Donor$^{CLYBL}$ (5770 bp)
TCGCGCGTTTCGGTGATGACGGTGAAAACCTCTGACACATGCAGCTCCCGGAGACGGTCACAGCTTGT
CTGTAAGCGGATGCCGGGAGCAGACAAGCCCGTCAGGGCGCGTCAGCGGGTGTTGGCGGGTGTCGGGG
CTGGCTTAACTATGCGGCATCAGAGCAGATTGTACTGAGAGTGCACCATATGCGGTGTGAAATACCGC
ACAGATGCGTAAGGAGAAAATACCGCATCAGGCGCCATTCGCCATTCAGGCTGCGCAACTGTTGGGAA
GGGCGATCGGTGCGGGCCTCTTCGCTATTACGCCAGCTGGCGAAAGGGGGATGTGCTGCAAGGCGATT
AAGTTGGGTAACGCCAGGGTTTTCCCAGTCACGACGTTGTAAAACGACGGCCAGTGAATTCGAGCTCG
GTACCTCGCGAATGCATCTAGATGACGACCGCCGGAAATATTCAGAATGCTTTAATGGAGTAAGAAGG
GCCACTGCTTAGAGTGGGGCTGAAGAGAAAAGAAAGAAGGAGACCCTGCGGAAGCGGCAATACCAGGA
AGGCAGTTGTATGCACATCATGCAGACCAGGAGTAGCCTAGGGGTAGGGGACATGCCTGGGCTGGACA
GTGGAATTTGTGGGAAATCAGATTTCCCTGAGCAAAAACCAGTGGGCAGCAGCCAAACTGGTGAGCAA
GAACTGGCTGTGGTGGCTGGGCCCGAATTGGGAGCTACACCTGCCCGGGCAGGTGTAGCTGCGAGGGA
GGTCTCTGCCAACAACTCAGCTGTGCCGTGCCTGCTGAACGGCCTCACCTTGGCCTCTGGCTCTGTTG
CAGCTGTGTGGGGCTCGTGCTCCTACATACCACGCGTGTTCGCGTGTTAGCTTCCACGGGGTTGGGGT
TGCGCCTTTTCCAAGGCAGCCCTGGGTTTGCGCAGGGACGCGGCTGCTCTGGGCGTGGTTCCGGGAAA
CGCAGCGGCGCCGACCCTGGGTCTCGCACATTCTTCACGTCCGTTCGCAGCGTCACCCGGATCTTCGC
CGCTACCCTTGTGGGCCCCCCGGCGACGCTTCCTGCTCCGCCCCTAAGTCGGGAAGGTTCCTTGCGGT
TCGCGGCGTGCCGGACGTGACAAACGGAAGCCGCACGTCTCACTAGTACCCTCGCAGACGGACAGCGC
CAGGGAGCAATGGCAGCGCGCCGACCGCGATGGGCTGTGGCCAATAGCGGCTGCTCAGCAGGGCGCGC

CGAGAGCAGCGGCCGGGAAGGGGCGGTGCGGGAGGCGGGGTGTGGGGCGGTAGTGTGGGCCCTGTTCC
TGCCCGCGCGGTGTTCCGCATTCTGCAAGCCTCCGGAGCGCACGTCGGCAGTCGGCTCCCTCGTTGAC
CGAATCACCGACCTCTCTCCCCACCGGGTACGTCGCTAGCCCACCATGACCGAGTACAAGCCCACGGT
GCGCCTCGCCACCCGCGACGACGTCCCCCGGGCCGTACGCACCCTCGCCGCCGCGTTCGCCGACTACC
CCGCCACGCGCCACACCGTCGACCCGGACCGCCACATCGAGCGGGTCACCGAGCTGCAAGAACTCTTC
CTCACGCGCGTCGGGCTCGACATCGGCAAGGTGTGGGTCGCGGACGACGGCGCCGCGGTGGCGGTCTG
GACCACGCCGGAGAGCGTCGAAGCGGGGGCGGTGTTCGCCGAGATCGGCCCGCGCATGGCCGAGTTGA
GCGGTTCCCGGCTGGCCGCGCAGCAACAGATGGAAGGCCTCCTGGCGCCGCACCGGCCCAAGGAGCCC
GCGTGGTTCCTGGCCACCGTCGGCGTCTCGCCCGACCACCAGGGCAAGGGTCTGGGCAGCGCCGTCGT
GCTCCCCGGAGTGGAGGCGGCCGAGCGCGCCGGGGTGCCCGCCTTCCTGGAGACCTCCGCGCCCCGCA
ACCTCCCCTTCTACGAGCGGCTCGGCTTCACCGTCACCGCCGACGTCGAGGTGCCCGAAGGACCGCGC
ACCTGGTGCATGACCCGCAAGCCCGGTGCCGGATCGGGAGAGGGCAGAGGAAGTCTGCTAACATGCGG
TGACGTCGAGGAGAATCCTGGCCCACCGGTCGCCACCATGGTGAGCAAGGGCGAGGAGCTGTTCACCG
GGGTGGTGCCCATCCTGGTCGAGCTGGACGGCGACGTAAACGGCCACAAGTTCAGCGTGTCCGGCGAG
GGCGAGGGCGATGCCACCTACGGCAAGCTGACCCTGAAGTTCATCTGCACCACCGGCAAGCTGCCCGT
GCCCTGGCCCACCCTCGTGACCACCCTGACCTACGGCGTGCAGTGCTTCAGCCGCTACCCCGACCACA
TGAAGCAGCACGACTTCTTCAAGTCCGCCATGCCCGAAGGCTACGTCCAGGAGCGCACCATCTTCTTC
AAGGACGACGGCAACTACAAGACCCGCGCCGAGGTGAAGTTCGAGGGCGACACCCTGGTGAACCGCAT
CGAGCTGAAGGGCATCGACTTCAAGGAGGACGGCAACATCCTGGGGCACAAGCTGGAGTACAACTACA
ACAGCCACAACGTCTATATCATGGCCGACAAGCAGAAGAACGGCATCAAGGTGAACTTCAAGATCCGC
CACAACATCGAGGACGGCAGCGTGCAGCTCGCCGACCACTACCAGCAGAACACCCCCATCGGCGACGG
CCCCGTGCTGCTGCCCGACAACCACTACCTGAGCACCCAGTCCGCCCTGAGCAAAGACCCCAACGAGA
AGCGCGATCACATGGTCCTGCTGGAGTTCGTGACCGCCGCCGGGATCACTCTCGGCATGGACGAGCTG
TACAAGTAAAGCGGCCGCGTCGAGTCTAGGATCAGCCTCGACTGTGCCTTCTAGTTGCCAGCCATCTG
TTGTTTGCCCCTCCCCCGTGCCTTCCTTGACCCTGGAAGGTGCCACTCCCACTGTCCTTTCCTAATAA
AATGAGGAAATTGCATCGCATTGTCTGAGTAGGTGTCATTCTATTCTGGGGGGTGGGGTGGGGCAGGA
CAGCAAGGGGGAGGATTGGGAAGACAATAGCAGGCATGCTGGGGATGCGGTGGGCTCTATGGAAGCTA
ACAAGCTAACAAGCTTGTTATCCTGTGGTAGAGTTTTACCGTGGACCAGAGCCATCTAGAGGACATTG
AGTTACCCACATACTTTCTGTGGGAGAGGCCAAGGAGTCAGGGGGGGAGGCCGGGACCTGAAGATCAA
CTCTTTACTGCAGAGATCCCTCCCTCCCTCTCTCTGTCCCATGGATCCAGAGGGAAGTTGAGGACAGT
TTCATGCCACATGCTTGACCCGACTCAACTGAGTAACTGTGGCCACTGAGAAGACTCTGTCCTTGCAA
ATAACTCTCCTTAGTGCCCATATTCTGCAGAATGTTTGAGTGGCACGAGTACTTCATTCGTCTAGAAA
TATTTCATGGTAGTCAGTTGCTAATAACAGGATATCTGCAGGTAGTGTGTTCTCAGGAGGCACACCTG
AAGCCTCAGTTTGGCCGCGTCGTATCGGATCCCGGGCCCGTCGACTGCAGAGGCCTGCATGCAAGCTT
GGCGTAATCATGGTCATAGCTGTTTCCTGTGTGAAATTGTTATCCGCTCACAATTCCACACAACATAC
GAGCCGGAAGCATAAAGTGTAAAGCCTGGGGTGCCTAATGAGTGAGCTAACTCACATTAATTGCGTTG
CGCTCACTGCCCGCTTTCCAGTCGGGAAACCTGTCGTGCCAGCTGCATTAATGAATCGGCCAACGCGC
GGGGAGAGGCGGTTTGCGTATTGGGCGCTCTTCCGCTTCCTCGCTCACTGACTCGCTGCGCTCGGTCG
TTCGGCTGCGGCGAGCGGTATCAGCTCACTCAAAGGCGGTAATACGGTTATCCACAGAATCAGGGGAT
AACGCAGGAAAGAACATGTGAGCAAAAGGCCAGCAAAAGGCCAGGAACCGTAAAAAGGCCGCGTTGCT
GGCGTTTTTCCATAGGCTCCGCCCCCCTGACGAGCATCACAAAAATCGACGCTCAAGTCAGAGGTGGC
GAAACCCGACAGGACTATAAAGATACCAGGCGTTTCCCCCTGGAAGCTCCCTCGTGCGCTCTCCTGTT
CCGACCCTGCCGCTTACCGGATACCTGTCCGCCTTTCTCCCTTCGGGAAGCGTGGCGCTTTCTCATAG
CTCACGCTGTAGGTATCTCAGTTCGGTGTAGGTCGTTCGCTCCAAGCTGGGCTGTGTGCACGAACCCC
CCGTTCAGCCCGACCGCTGCGCCTTATCCGGTAACTATCGTCTTGAGTCCAACCCGGTAAGACACGAC
TTATCGCCACTGGCAGCAGCCACTGGTAACAGGATTAGCAGAGCGAGGTATGTAGGCGGTGCTACAGA
GTTCTTGAAGTGGTGGCCTAACTACGGCTACACTAGAAGAACAGTATTTGGTATCTGCGCTCTGCTGA
AGCCAGTTACCTTCGGAAAAAGAGTTGGTAGCTCTTGATCCGGCAAACAAACCACCGCTGGTAGCGGT

GGTTTTTTTGTTTGCAAGCAGCAGATTACGCGCAGAAAAAAAGGATCTCAAGAAGATCCTTTGATCTT
TTCTACGGGGTCTGACGCTCAGTGGAACGAAAACTCACGTTAAGGGATTTTGGTCATGAGATTATCAA
AAAGGATCTTCACCTAGATCCTTTTAAATTAAAAATGAAGTTTTAAATCAATCTAAAGTATATATGAG
TAAACTTGGTCTGACAGTTACCAATGCTTAATCAGTGAGGCACCTATCTCAGCGATCTGTCTATTTCG
TTCATCCATAGTTGCCTGACTCCCCGTCGTGTAGATAACTACGATACGGGAGGGCTTACCATCTGGCC
CCAGTGCTGCAATGATACCGCGAGACCCACGCTCACCGGCTCCAGATTTATCAGCAATAAACCAGCCA
GCCGGAAGGGCCGAGCGCAGAAGTGGTCCTGCAACTTTATCCGCCTCCATCCAGTCTATTAATTGTTG
CCGGGAAGCTAGAGTAAGTAGTTCGCCAGTTAATAGTTTGCGCAACGTTGTTGCCATTGCTACAGGCA
TCGTGGTGTCACGCTCGTCGTTTGGTATGGCTTCATTCAGCTCCGGTTCCCAACGATCAAGGCGAGTT
ACATGATCCCCCATGTTGTGCAAAAAAGCGGTTAGCTCCTTCGGTCCTCCGATCGTTGTCAGAAGTAA
GTTGGCCGCAGTGTTATCACTCATGGTTATGGCAGCACTGCATAATTCTCTTACTGTCATGCCATCCG
TAAGATGCTTTTCTGTGACTGGTGAGTACTCAACCAAGTCATTCTGAGAATAGTGTATGCGGCGACCG
AGTTGCTCTTGCCCGGCGTCAATACGGGATAATACCGCGCCACATAGCAGAACTTTAAAAGTGCTCAT
CATTGGAAAACGTTCTTCGGGGCGAAAACTCTCAAGGATCTTACCGCTGTTGAGATCCAGTTCGATGT
AACCCACTCGTGCACCCAACTGATCTTCAGCATCTTTTACTTTCACCAGCGTTTCTGGGTGAGCAAAA
ACAGGAAGGCAAAATGCCGCAAAAAAGGGAATAAGGGCGACACGGAAATGTTGAATACTCATACTCTT
CCTTTTTCAATATTATTGAAGCATTTATCAGGGTTATTGTCTCATGAGCGGATACATATTTGAATGTA
TTTAGAAAAATAAACAAATAGGGGTTCCGCGCACATTTCCCCGAAAAGTGCCACCTGACGTCTAAGAA
ACCATTATTATCATGACATTAACCTATAAAAATAGGCGTATCACGAGGCCCTTTCGTC

**Map and nucleotide sequence of *CLYBL*-targeting plasmid AD60_pEP.Donor$^{CLYBL}$.** Orange regions, sequences identical to the human *CLYBL* safe harbour locus; hPGK promoter, human phosphoglycerate kinase 1 gene (*PGK1*) regulatory sequences; PuroR, puromycin N-acetyltransferase resistance gene; T2A, “self-cleaving” 2A peptide coding sequence from the *Thosea asigna* virus capsid protein; EGFP, open reading frame of enhanced green fluorescence protein gene; bGH poly(A) signal, bovine growth hormone gene (*GH1*) polyadenylation signal; AmpR, *β-lactamase* ampicillin resistance gene.

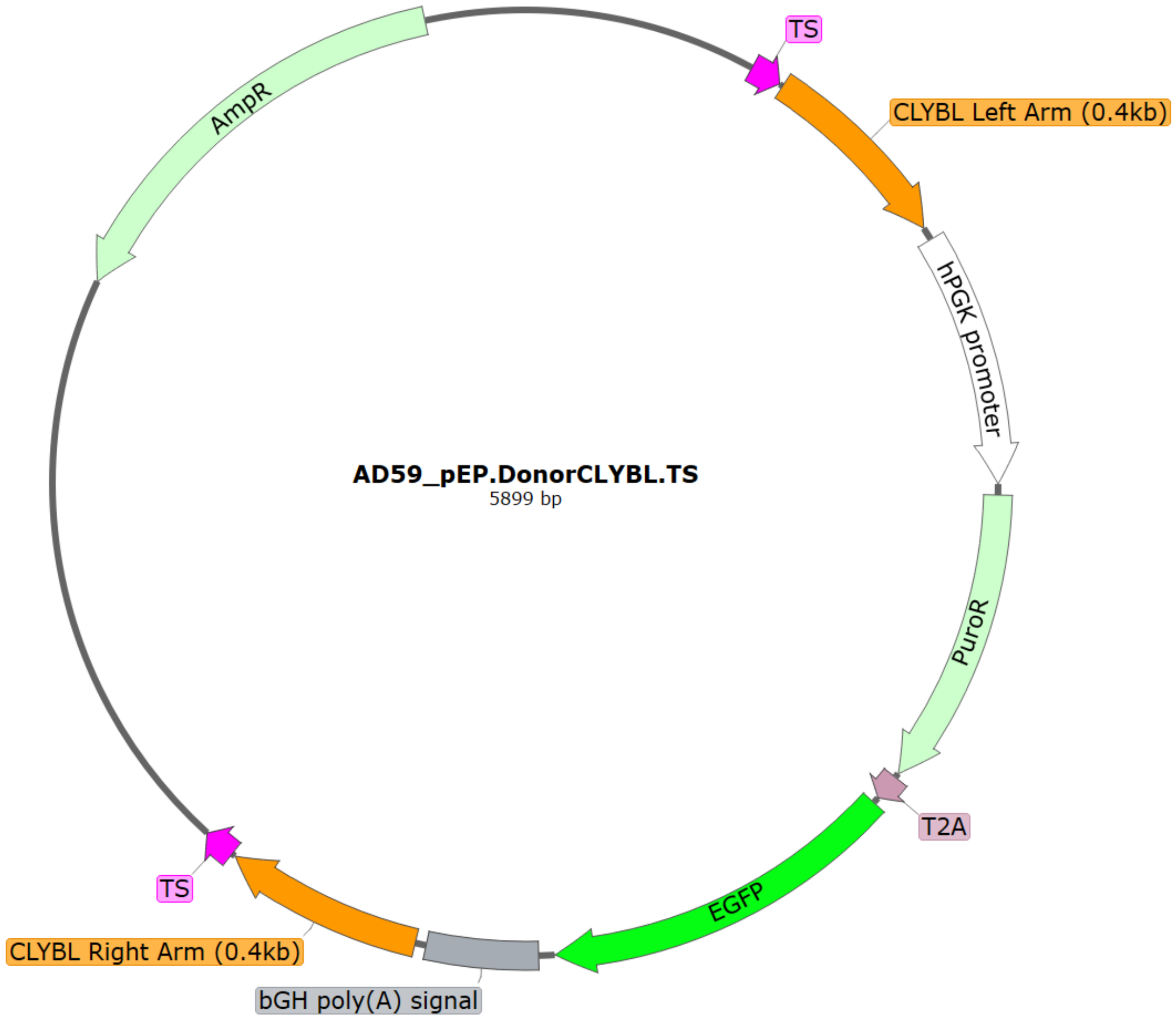


>AD59_pEP.Donor[CLYBL.TS] (5899 bp)

```
TCGCGCGTTTCGGTGATGACGGTGAAAACCTCTGACACATGCAGCTCCCGGAGACGGTCACAGCTTGT
CTGTAAGCGGATGCCGGGAGCAGACAAGCCCGTCAGGGCGCGTCAGCGGGTGTTGGCGGGTGTCGGGG
CTGGCTTAACTATGCGGCATCAGAGCAGATTGTACTGAGAGTGCACCATATGCGGTGTGAAATACCGC
ACAGATGCGTAAGGAGAAAATACCGCATCAGGCGCCATTCGCCATTCAGGCTGCGCAACTGTTGGGAA
GGGCGATCGGTGCGGGCCTCTTCGCTATTACGCCAGCTGGCGAAAGGGGGATGTGCTGCAAGGCGATT
AAGTTGGGTAACGCCAGGGTTTTCCCAGTCACGACGTTGTAAAACGACGGCCAGTGAATTCGAGCTCG
GTACCTCGCGAATGCATCTAGATATCGGATCCCGGGCCCGTCGACTGCAGAGGCGGCCGCCAGCTGT
GTGGGGCTCGTGCTCCTACATACCGTTATCCTGTGGTAGAGTTTTACCGTGGACCAGATTTAAATATT
CAGAATGCTTTAATGGAGTAAGAAGGGCCACTGCTTAGAGTGGGGCTGAAGAGAAAAGAAAGAAGGAG
ACCCTGCGGAAGCGGCAATACCAGGAAGGCAGTTGTATGCACATCATGCAGACCAGGAGTAGCCTAGG
GGTAGGGGACATGCCTGGGCTGGACAGTGGAATTTGTGGGAAATCAGATTTCCCTGAGCAAAAACCAG
TGGGCAGCAGCCAAACTGGTGAGCAAGAACTGGCTGTGGTGGCTGGGCCCGAATTGGGAGCTACACCT
GCCCGGGCAGGTGTAGCTGCGAGGGAGGTCTCTGCCAACAACTCAGCTGTGCCGTGCCTGCTGAACGG
CCTCACCTTGGCCTCTGGCTCTGTTGCAGCTGTGTGGGGCTCGTGCTCCTACATACCACGCGTGTTCG
CGTGTTAGCTTCCACGGGGTTGGGGTTGCGCCTTTTCCAAGGCAGCCCTGGGTTTGCGCAGGGACGCG
GCTGCTCTGGGCGTGGTTCCGGGAAACGCAGCGGCGCCGACCCTGGGTCTCGCACATTCTTCACGTCC
GTTCGCAGCGTCACCCGGATCTTCGCCGCTACCCTTGTGGGCCCCCCGGCGACGCTTCCTGCTCCGCC
CCTAAGTCGGGAAGGTTCCTTGCGGTTCGCGGCGTGCCGGACGTGACAAACGGAAGCCGCACGTCTCA
CTAGTACCCTCGCAGACGGACAGCGCCAGGGAGCAATGGCAGCGCGCCGACCGCGATGGGCTGTGGCC
```

AATAGCGGCTGCTCAGCAGGGCGCGCCGAGAGCAGCGGCCGGGAAGGGGCGGTGCGGGAGGCGGGGTG
TGGGGCGGTAGTGTGGGCCCTGTTCCTGCCCGCGCGGTGTTCCGCATTCTGCAAGCCTCCGGAGCGCA
CGTCGGCAGTCGGCTCCCTCGTTGACCGAATCACCGACCTCTCTCCCCACCGGGTACGTCGCTAGCCC
ACCATGACCGAGTACAAGCCCACGGTGCGCCTCGCCACCCGCGACGACGTCCCCCGGGCCGTACGCAC
CCTCGCCGCCGCGTTCGCCGACTACCCCGCCACGCGCCACACCGTCGACCCGGACCGCCACATCGAGC
GGGTCACCGAGCTGCAAGAACTCTTCCTCACGCGCGTCGGGCTCGACATCGGCAAGGTGTGGGTCGCG
GACGACGGCGCCGCGGTGGCGGTCTGGACCACGCCGGAGAGCGTCGAAGCGGGGGCGGTGTTCGCCGA
GATCGGCCCGCGCATGGCCGAGTTGAGCGGTTCCCGGCTGGCCGCGCAGCAACAGATGGAAGGCCTCC
TGGCGCCGCACCGGCCCAAGGAGCCCGCGTGGTTCCTGGCCACCGTCGGCGTCTCGCCCGACCACCAG
GGCAAGGGTCTGGGCAGCGCCGTCGTGCTCCCCGGAGTGGAGGCGGCCGAGCGCGCCGGGGTGCCCGC
CTTCCTGGAGACCTCCGCGCCCCGCAACCTCCCCTTCTACGAGCGGCTCGGCTTCACCGTCACCGCCG
ACGTCGAGGTGCCCGAAGGACCGCGCACCTGGTGCATGACCCGCAAGCCCGGTGCCGGATCGGGAGAG
GGCAGAGGAAGTCTGCTAACATGCGGTGACGTCGAGGAGAATCCTGGCCCACCGGTCGCCACCATGGT
GAGCAAGGGCGAGGAGCTGTTCACCGGGGTGGTGCCCATCCTGGTCGAGCTGGACGGCGACGTAAACG
GCCACAAGTTCAGCGTGTCCGGCGAGGGCGAGGGCGATGCCACCTACGGCAAGCTGACCCTGAAGTTC
ATCTGCACCACCGGCAAGCTGCCCGTGCCCTGGCCCACCCTCGTGACCACCCTGACCTACGGCGTGCA
GTGCTTCAGCCGCTACCCCGACCACATGAAGCAGCACGACTTCTTCAAGTCCGCCATGCCCGAAGGCT
ACGTCCAGGAGCGCACCATCTTCTTCAAGGACGACGGCAACTACAAGACCCGCGCCGAGGTGAAGTTC
GAGGGCGACACCCTGGTGAACCGCATCGAGCTGAAGGGCATCGACTTCAAGGAGGACGGCAACATCCT
GGGGCACAAGCTGGAGTACAACTACAACAGCCACAACGTCTATATCATGGCCGACAAGCAGAAGAACG
GCATCAAGGTGAACTTCAAGATCCGCCACAACATCGAGGACGGCAGCGTGCAGCTCGCCGACCACTAC
CAGCAGAACACCCCCATCGGCGACGGCCCCGTGCTGCTGCCCGACAACCACTACCTGAGCACCCAGTC
CGCCCTGAGCAAAGACCCCAACGAGAAGCGCGATCACATGGTCCTGCTGGAGTTCGTGACCGCCGCCG
GGATCACTCTCGGCATGGACGAGCTGTACAAGTAAAGCGGCCGCGTCGAGTCTAGGATCAGCCTCGAC
TGTGCCTTCTAGTTGCCAGCCATCTGTTGTTTGCCCCTCCCCCGTGCCTTCCTTGACCCTGGAAGGTG
CCACTCCCACTGTCCTTTCCTAATAAAATGAGGAAATTGCATCGCATTGTCTGAGTAGGTGTCATTCT
ATTCTGGGGGGTGGGGTGGGGCAGGACAGCAAGGGGGAGGATTGGGAAGACAATAGCAGGCATGCTGG
GGATGCGGTGGGCTCTATGGAAGCTAACAAGCTAACAAGCTTGTTATCCTGTGGTAGAGTTTTACCGT
GGACCAGAGCCATCTAGAGGACATTGAGTTACCCACATACTTTCTGTGGGAGAGGCCAAGGAGTCAGG
GGGGGAGGCCGGGACCTGAAGATCAACTCTTTACTGCAGAGATCCCTCCCTCCCTCTCTCTGTCCCAT
GGATCCAGAGGGAAGTTGAGGACAGTTTCATGCCACATGCTTGACCCGACTCAACTGAGTAACTGTGG
CCACTGAGAAGACTCTGTCCTTGCAAATAACTCTCCTTAGTGCCCATATTCTGCAGAATGTTTGAGTG
GCACGAGTACTTCATTCGTCTAGAAATATTTCATGGTAGTCAGTTGCTAATAACAGGATATCTGCAGG
TAGTGTGTTCTCAGGAGGCACACCTGAAGCCTCAGTTTAAACCAGCTGTGTGGGGCTCGTGCTCCTAC
ATACCGTTATCCTGTGGTAGAGTTTTACCGTGGACCAGCGCCGGCGCCTGCATGCAAGCTTGGCGTAA
TCATGGTCATAGCTGTTTCCTGTGTGAAATTGTTATCCGCTCACAATTCCACACAACATACGAGCCGG
AAGCATAAAGTGTAAAGCCTGGGGTGCCTAATGAGTGAGCTAACTCACATTAATTGCGTTGCGCTCAC
TGCCCGCTTTCCAGTCGGGAAACCTGTCGTGCCAGCTGCATTAATGAATCGGCCAACGCGCGGGGAGA
GGCGGTTTGCGTATTGGGCGCTCTTCCGCTTCCTCGCTCACTGACTCGCTGCGCTCGGTCGTTCGGCT
GCGGCGAGCGGTATCAGCTCACTCAAAGGCGGTAATACGGTTATCCACAGAATCAGGGGATAACGCAG
GAAAGAACATGTGAGCAAAAGGCCAGCAAAAGGCCAGGAACCGTAAAAAGGCCGCGTTGCTGGCGTTT
TTCCATAGGCTCCGCCCCCCTGACGAGCATCACAAAAATCGACGCTCAAGTCAGAGGTGGCGAAACCC
GACAGGACTATAAAGATACCAGGCGTTTCCCCCTGGAAGCTCCCTCGTGCGCTCTCCTGTTCCGACCC
TGCCGCTTACCGGATACCTGTCCGCCTTTCTCCCTTCGGGAAGCGTGGCGCTTTCTCATAGCTCACGC
TGTAGGTATCTCAGTTCGGTGTAGGTCGTTCGCTCCAAGCTGGGCTGTGTGCACGAACCCCCCGTTCA
GCCCGACCGCTGCGCCTTATCCGGTAACTATCGTCTTGAGTCCAACCCGGTAAGACACGACTTATCGC
CACTGGCAGCAGCCACTGGTAACAGGATTAGCAGAGCGAGGTATGTAGGCGGTGCTACAGAGTTCTTG
AAGTGGTGGCCTAACTACGGCTACACTAGAAGAACAGTATTTGGTATCTGCGCTCTGCTGAAGCCAGT

```
TACCTTCGGAAAAAGAGTTGGTAGCTCTTGATCCGGCAAACAAACCACCGCTGGTAGCGGTGGTTTTT
TTGTTTGCAAGCAGCAGATTACGCGCAGAAAAAAAGGATCTCAAGAAGATCCTTTGATCTTTTCTACG
GGGTCTGACGCTCAGTGGAACGAAAACTCACGTTAAGGGATTTTGGTCATGAGATTATCAAAAAGGAT
CTTCACCTAGATCCTTTTAAATTAAAAATGAAGTTTTAAATCAATCTAAAGTATATATGAGTAAACTT
GGTCTGACAGTTACCAATGCTTAATCAGTGAGGCACCTATCTCAGCGATCTGTCTATTTCGTTCATCC
ATAGTTGCCTGACTCCCCGTCGTGTAGATAACTACGATACGGGAGGGCTTACCATCTGGCCCCAGTGC
TGCAATGATACCGCGAGACCCACGCTCACCGGCTCCAGATTTATCAGCAATAAACCAGCCAGCCGGAA
GGGCCGAGCGCAGAAGTGGTCCTGCAACTTTATCCGCCTCCATCCAGTCTATTAATTGTTGCCGGGAA
GCTAGAGTAAGTAGTTCGCCAGTTAATAGTTTGCGCAACGTTGTTGCCATTGCTACAGGCATCGTGGT
GTCACGCTCGTCGTTTGGTATGGCTTCATTCAGCTCCGGTTCCCAACGATCAAGGCGAGTTACATGAT
CCCCCATGTTGTGCAAAAAAGCGGTTAGCTCCTTCGGTCCTCCGATCGTTGTCAGAAGTAAGTTGGCC
GCAGTGTTATCACTCATGGTTATGGCAGCACTGCATAATTCTCTTACTGTCATGCCATCCGTAAGATG
CTTTTCTGTGACTGGTGAGTACTCAACCAAGTCATTCTGAGAATAGTGTATGCGGCGACCGAGTTGCT
CTTGCCCGGCGTCAATACGGGATAATACCGCGCCACATAGCAGAACTTTAAAAGTGCTCATCATTGGA
AAACGTTCTTCGGGGCGAAAACTCTCAAGGATCTTACCGCTGTTGAGATCCAGTTCGATGTAACCCAC
TCGTGCACCCAACTGATCTTCAGCATCTTTTACTTTCACCAGCGTTTCTGGGTGAGCAAAAACAGGAA
GGCAAAATGCCGCAAAAAAGGGAATAAGGGCGACACGGAAATGTTGAATACTCATACTCTTCCTTTTT
CAATATTATTGAAGCATTTATCAGGGTTATTGTCTCATGAGCGGATACATATTTGAATGTATTTAGAA
AAATAAACAAATAGGGGTTCCGCGCACATTTCCCCGAAAAGTGCCACCTGACGTCTAAGAAACCATTA
TTATCATGACATTAACCTATAAAAATAGGCGTATCACGAGGCCCTTTCGTC
```

**Map and nucleotide sequence of *CLYBL*-targeting plasmid AD59_pEP.Donor$^{CLYBL.TS}$.** Orange regions, sequences homologous to the human *CLYBL* safe harbour locus; Magenta arrows, gRNA$^{CLYBL}$ target site (TS); hPGK promoter, human phosphoglycerate kinase 1 gene (*PGK1*) regulatory sequences; PuroR, puromycin N-acetyltransferase resistance gene; T2A, "self-cleaving" 2A peptide coding sequence from the *Thosea asigna* virus capsid protein; EGFP, open reading frame of enhanced green fluorescence protein gene; bGH poly(A) signal, bovine growth hormone gene (*GH1*) polyadenylation signal; AmpR, *β-lactamase* ampicillin resistance gene.

```
cutadapt -a AGATCGGAAGAGCACACG -A CTGTCTCTTATACACATC -o out_R1.fastq
-p out_R2.fastq reads_R1.fastq reads_R2.fastq
```

**Script for adapter trimming of raw NGS reads.** The script supports trimming of paired-end reads with Cutadapt 2.10. The output files and input reads are highlighted in blue and red, respectively.

```
#!/bin/bash
docker run -v ${PWD}:/DATA -w /DATA -i pinellolab/crispresso2
CRISPResso --fastq_r1 AM.trimedR1.fastq --fastq_r2 AM.trimedR2.fastq
--amplicon_seq
CTGCCTAACAGGAGGTGGGGGTTAGACCCAATATCAGGAGACTAGGAAGGAGGAGGCCTAAGGATGGG
GCTTTTCTGTCACCAATCCTGTCCCTAGTGGCCCCACTGTGGGGTGGAGGGGACAGATAAAAGTACCC
AGAACCAGAGCCACATTAACCGGCCCTGGG --guide_seq ggggccactagggacaggat --
amplicon_name Mock --name AM --amplicon_min_alignment_score 60 --
quantification_window_size 20 --min_average_read_quality 0 --
min_single_bp_quality 0 --min_bp_quality_or_N 0 --
```

```
exclude_bp_from_left 15 --exclude_bp_from_right 15 --
plot_window_size 30 --min_frequency_alleles_around_cut_to_plot 0.1 -
-max_rows_alleles_around_cut_to_plot 100
```

**Script for NGS quantification of on-target indel frequencies induced by regular and high-specificity CRISPR complexes at *AAVS1*.** Script for deep sequencing analysis using CRISPResso2 software and Docker containerization systems. The trimmed input reads and *AAVS1* amplicon sequence are highlighted in red and blue, respectively.

```
#!/bin/bash
docker run -v ${PWD}:/DATA -w /DATA -i pinellolab/crispresso2
CRISPResso --fastq_r1 BM.trimedR1.fastq --fastq_r2 BM.trimedR2.fastq
--amplicon_seq
TAGGGGAAAGAGAGGAGCCTGATTCTGCTAAGGCATAGAAACAGATTGTCAGCCATTCCTGCAGGTAA
CACCACTATTGTATATGGGCCTTTGAGATGTTTTTTTTGTTGTTTTTTTTTTTCATGTCTGACACCTA
TGGCTCCACTGGACCCCATGGGTCTACCTAGACCCACCAATCCTGCCCCTGTGGCCCCACTCAGAAAT
GATTTAGCCCACAGGAGGATGG --guide_seq TGGGGCCACAGGGGCAGGAT --
amplicon_name Mock --name BM --amplicon_min_alignment_score 60 --
quantification_window_size 20 --min_average_read_quality 0 --
min_single_bp_quality 0 --min_bp_quality_or_N 0 --
exclude_bp_from_left 15 --exclude_bp_from_right 15 --
plot_window_size 30 --min_frequency_alleles_around_cut_to_plot 0.1 -
-max_rows_alleles_around_cut_to_plot 100
```

**Script for NGS quantification of off-target indel frequencies induced by regular and high-specificity CRISPR complexes at *BBOX1*.** Script for deep sequencing analysis using CRISPResso2 software and Docker containerization systems. The trimmed input reads and *BBOX1* amplicon sequence are highlighted in red and blue, respectively.

```
#!/bin/bash
docker run -v ${PWD}:/DATA -w /DATA -i pinellolab/crispresso2
CRISPResso --fastq_r1 CM.trimedR1.fastq --fastq_r2 CM.trimedR2.fastq
--amplicon_seq
GGCTGGTCCCTGAAGACATCATCAGGCTCCACTGTGAGAAGCCTTGGATTCCTCATCCCAGAGCCCGC
TTCAGAGTAACAGCTTTTCCCTAAAAGCAAATTAAGGTCAGGGACCATCAGGGACAGGATGGGACGGG
AGGAGCCTCACTGTACCCAGCTCTGGCCTACAAGCTGTGAACA --guide_seq
GGGACCATCAGGGACAGGAT --amplicon_name Mock --name CM --
amplicon_min_alignment_score 60 --quantification_window_size 20 --
min_average_read_quality 0 --min_single_bp_quality 0 --
min_bp_quality_or_N 0 --exclude_bp_from_left 15 --
exclude_bp_from_right 15 --plot_window_size 30 --
min_frequency_alleles_around_cut_to_plot 0.1 --
max_rows_alleles_around_cut_to_plot 100
```

**Script for NGS quantification of off-target indel frequencies induced by regular and high-specificity CRISPR complexes at *CPNE5*.** Script for deep sequencing analysis using

CRISPResso2 software and Docker containerization systems. The trimmed input reads and *CPNE5* amplicon sequence are highlighted in red and blue, respectively.
