# Supplementary material for: Precise homology-directed installation of large genomic edits in human cells with cleaving and nicking high-specificity Cas9 variants
Source: Nucleic Acids Res. 2023 Mar 17;51(7):3465–84. doi: 10.1093/nar/gkad165 (PMC10123109; doi:10.1093/nar/gkad165)
Supplement: gkad165_Supplemental_Files [file gkad165_supplemental_files.zip › Wang et al. Supplementary Figures S1-S12.docx]

**
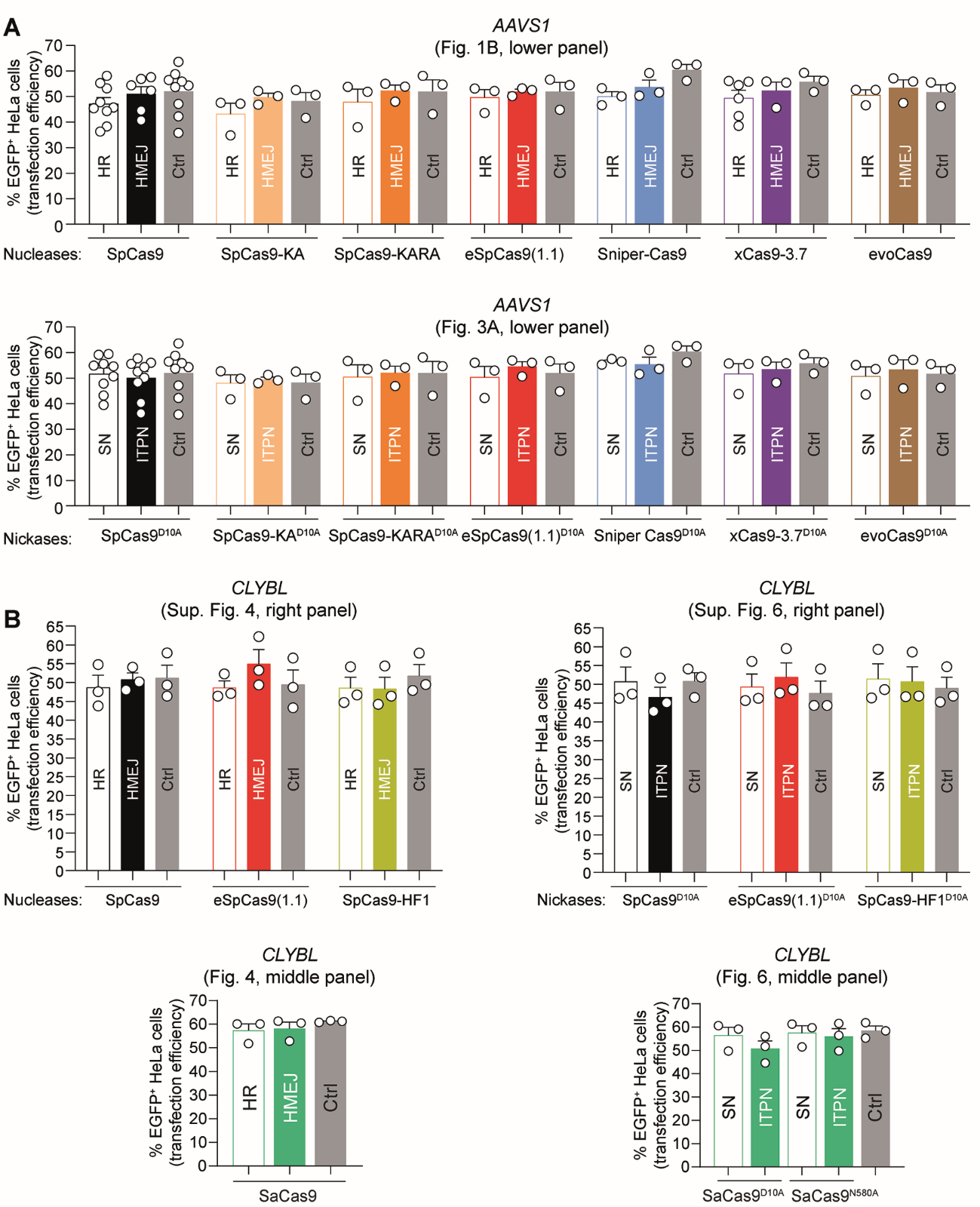
**

**Supplementary Figure S1. Controlling for comparable transfection efficiencies during genome editing experiments.** Cells were transfected with constructs harbouring donor templates and expression units for cleaving or nicking CRISPR complexes to assess genome editing based on single nicking (SN), *in trans* paired nicking (ITPN), homologous recombination (HR) and homology-mediated end joining (HMEJ). Transfection reactions containing plasmids encoding an irrelevant gRNA instead of a target sequence-specific gRNA provided for controls (Ctrl). Transfection efficiencies corresponding to representative genome editing experiments targeting *AAVS1* and *CLYBL* alleles using the indicated nucleases and nickases (panels **A** and **B**, respectively), were determined by reporter-directed flow cytometry at 3 days post-transfection. Non-transfected cells served as negative controls to set up the thresholds for background fluorescence. At least 10,000 live single cells were acquired per sample. Bars and error bars represent, respectively, means and standard deviations, respectively, of at least 3 biological replicates.


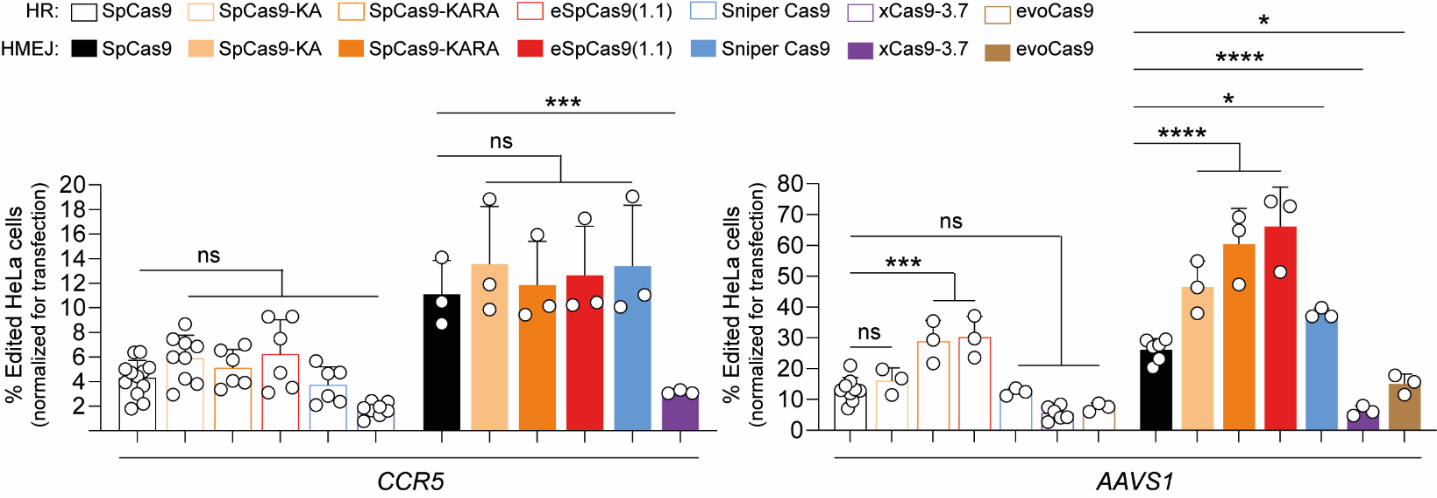


**Supplementary Figure S2. Comparing DSB-dependent genome editing using SpCas9 versus high-specificity SpCas9 variants.** Regrouping of datasets presented in **Figure 1B** corresponding to HeLa cells exposed to the indicated nucleases addressed to *CCR5* or *AAVS1* together with regular or target site-modified donor constructs (canonical HR or HMEJ strategies, respectively). Data are shown as mean ± SD of at least 3 independent biological replicates. Significant differences between the indicated datasets were calculated by two-way ANOVA followed by Šidák’s multiple comparisons tests; ****P<0.0001, ***0.0001<P<0.001, *0.01<P<0.05; P> 0.05 was considered non-significant (ns).


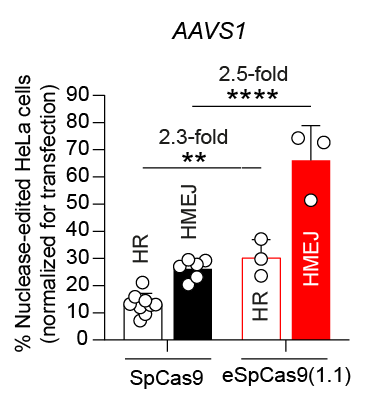


**Supplementary Figure S3. Comparing DSB-dependent genome editing using SpCas9 versus eSpCas9(1.1).** Plotting of datasets presented in **Figure 1B** corresponding to HeLa cells treated with SpCas9:gRNA^S1^ and high-specificity eSpCas9(1.1):gRNA^S1^ together with regular and target site-modified donors targeting the *AAVS1* locus (canonical HR and HMEJ strategies, respectively). Data are presented as mean ± SD of at least 3 independent biological replicates. Significant differences between the indicated datasets were calculated by two-way ANOVA followed by Šidák’s multiple comparisons tests; ****P<0.0001, **0.001<P<0.01.


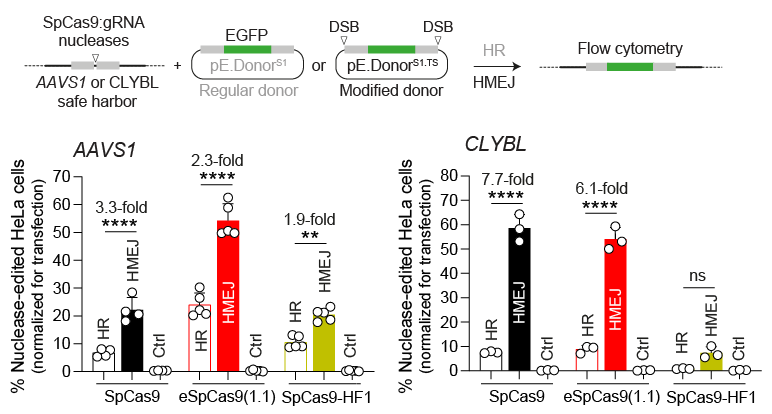


**Supplementary Figure S4. Comparing DSB-dependent genome editing using eSpCas9(1.1) versus SpCas9-HF1.** Nuclease-dependent genome editing frequencies in HeLa cells transfected with the depicted reagents targeting *AAVS1* and *CLYBL*, were measured by reporter-directed flow cytometry at 17 days post-transfection. Controls were provided by treating HeLa cells with nucleases and regular donor plasmids in the absence of locus-specific gRNAs. Data are shown as mean ± SD of at least 3 independent biological replicates. Significant differences between the indicated datasets were determined by two-way ANOVA followed by Šidák’s multiple comparison tests; ****P<0.0001, **0.001<P<0.01; P> 0.05 was considered non-significant (ns).


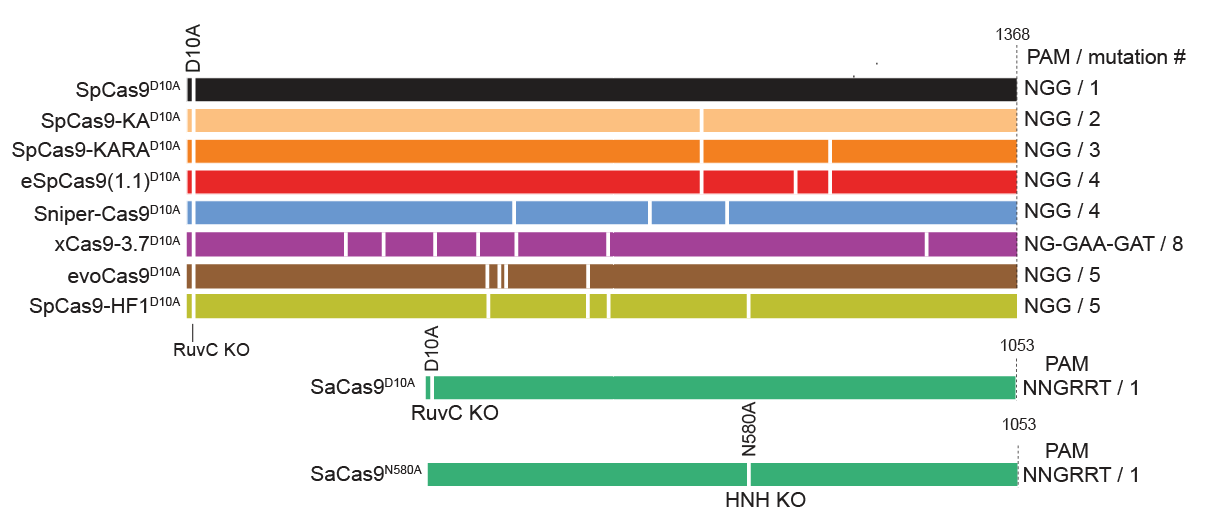


**Supplementary Figure S5. Schematics of engineered Cas9 nickases derived from *S. pyogenes* and *S. aureus* type II CRISPR systems.** The marked mutations-to-alanine endow the Cas9 proteins with sequence- and strand-specific DNA cleaving (nicking) activities once coupled to their cognate gRNAs. The main Cas9 protein domains and motifs are discriminated in the legend of Figure 1A.


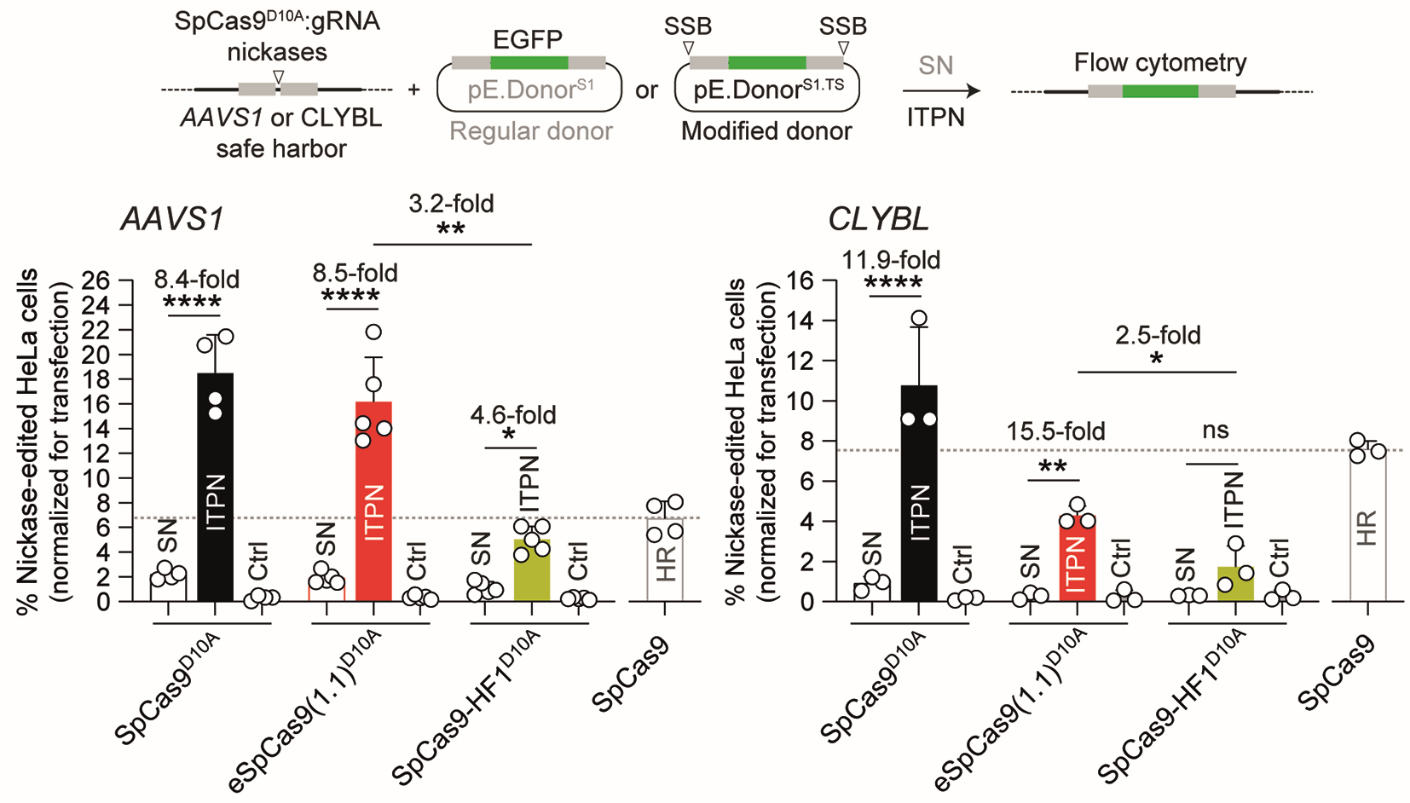


**Supplementary Figure S6. Comparing SSB-dependent genome editing using eSpCas9(1.1)D10A versus SpCas9-HF1.** Nickase-dependent genome editing frequencies in HeLa cells transfected with the indicated components targeting *AAVS1* and *CLYBL* were quantified by reporter-directed flow cytometry at 17 days post-transfection. Controls were provided by exposing HeLa cells to nickases and regular donor plasmids in the absence of locus-specific gRNAs. Results are plotted as mean ± SD of at least 3 independent biological replicates. Significant differences between the indicated datasets were assessed by two-way ANOVA followed by Šidák’s multiple comparisons tests, expect for the comparison of ITPN frequencies between eSpCas9(1.1)^D10A^ and SpCAs9-HF1^D10A^ where Student’s *t* tests were applied instead; ****P<0.0001, **0.001<P<0.01, *0.01<P<0.05; P> 0.05 was considered non-significant (ns).

**
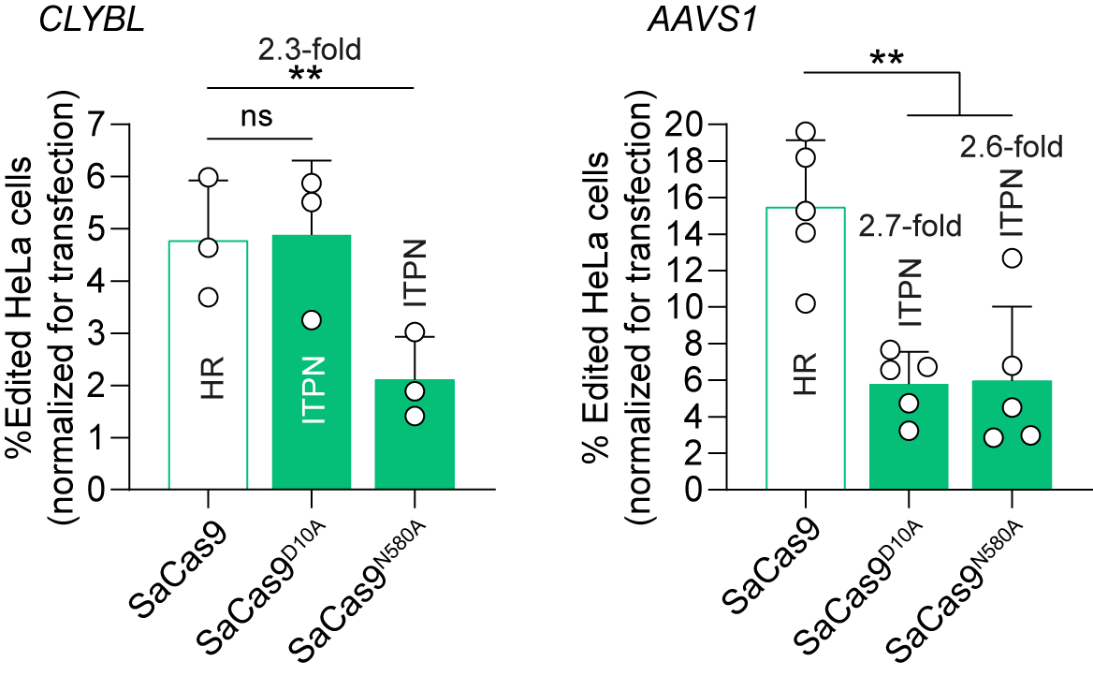
**

**Supplementary Figure S7. Comparing standard and in trans paired nicking genome editing using orthogonal Cas9 nuclease and nickases.** Plotting of datasets presented in **Figure 2** and **Figure 4** corresponding to HeLa cells subjected to SaCas9 nuclease and regular donors (canonical HR strategy) or to SaCas9^D10A^ or SaCas9^N580A^ nickases and target site-modified donors (ITPN strategy). Data are depicted as mean ± SD of a minimum of 3 independent biological replicates. Significant differences between the indicated datasets were calculated by one-way ANOVA followed by Dunnett’s multiple comparisons tests; **0.001<P<0.01; P>0.05 was considered non-significant (ns).


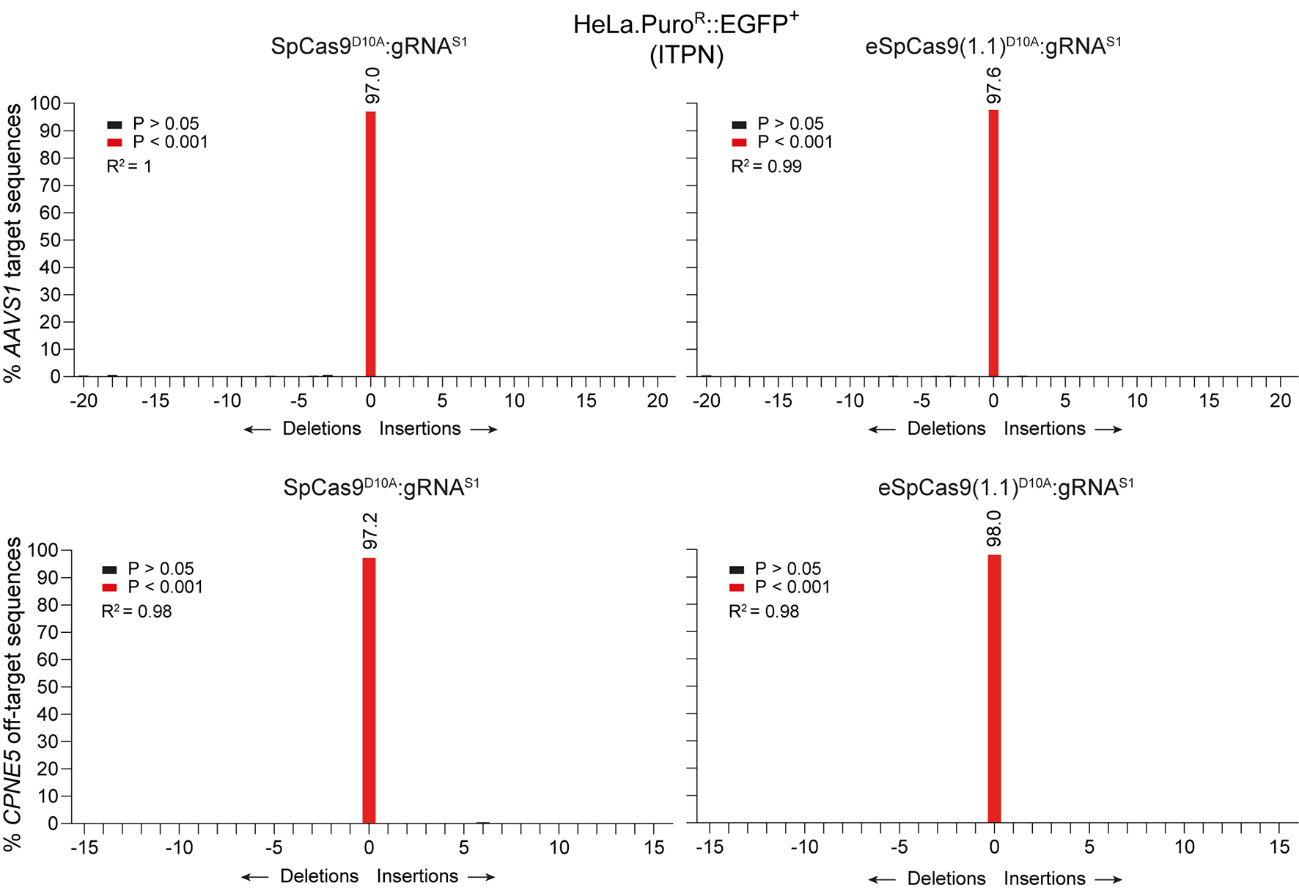


**Supplementary Figure S8. Probing mutagenic loads in HeLa cells genome edited through ITPN.** HeLa cells were exposed to the indicated *AAVS1*-targeting CRISPR complexes and matched ITPN donors encoding Puro^R^.EGFP were selected in puromycin. Genotyping of *AAVS1* and off-target *CPNE5* alleles in puromycin-resistant cell populations was performed through tracking of indels by decomposition (TIDE) analysis.


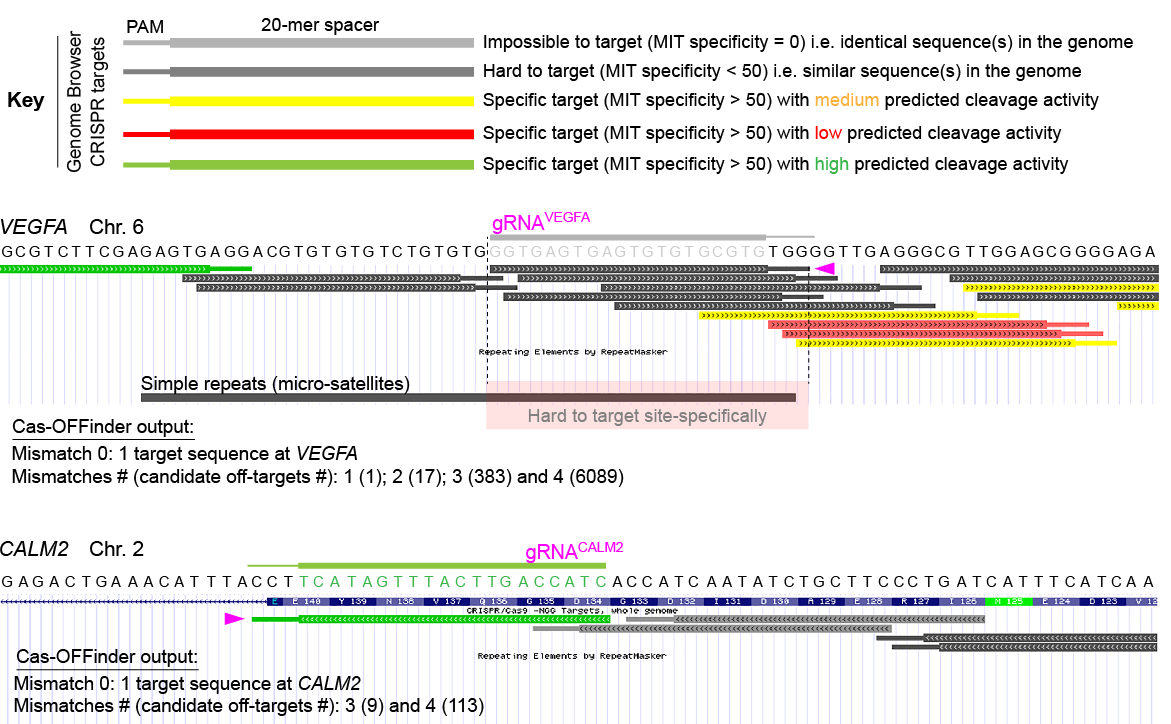


**Supplementary Figure S9. Specificity profiling of gRNAs targeting *VEGFA* and *CALM2*.** The CRISPR targets track from the UCSC Genome Browser (Assembly GRCh38/hg38) spanning the target sites for gRNA^VEGFA^ and gRNA^CALM2^ is shown. Target sites consisting of 20-mer spacers and the NGG PAM are colour-coded according to the predicted specificities and activities of their cognate *S. pyogenes* gRNAs. Independent gRNA specificity profiling of gRNA^VEGFA^ and gRNA^CALM2^, was performed by computing the predicted number of off-target sites using the Cas-OFFinder algorithm (Bae et al. 2014).


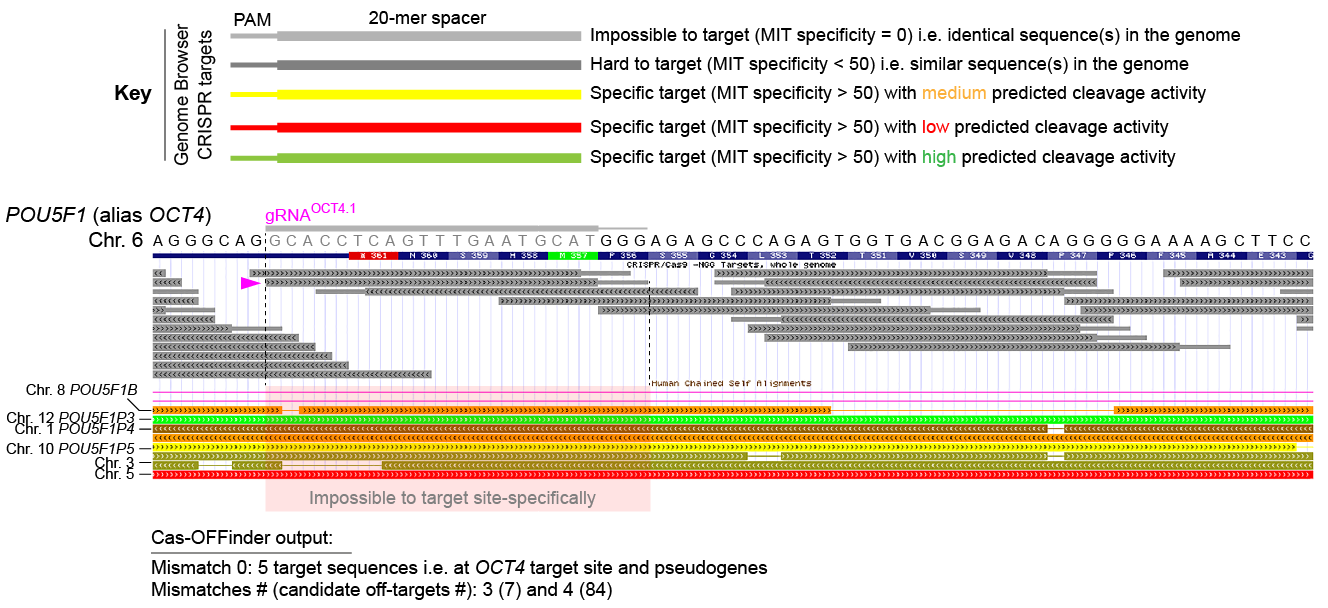


**Supplementary Figure S10. Specificity profiling of gRNA for OCT4 tagging.** The CRISPR targets track from the UCSC Genome Browser (Assembly GRCh38/hg38) covering the gRNA^OCT4.1^ target sequence is depicted. Target sequences consisting of 20-mer spacers and the NGG PAM are colour-coded according to the predicted specificities and activities of their cognate *S. pyogenes* gRNAs. The gRNA^OCT4.1^ target sequence gets an “impossible to target” score due to its 100% sequence identity with sequences in the *OCT4* pseudogenes *POU5F1B*, *POU5F1P3*, *POU5F1P4* and *POU5F1P5*. Independent gRNA specificity profiling of gRNA^OCT4.1^, was done by computing the predicted number of off-target sites using the Cas-OFFinder algorithm (Bae et al. 2014).


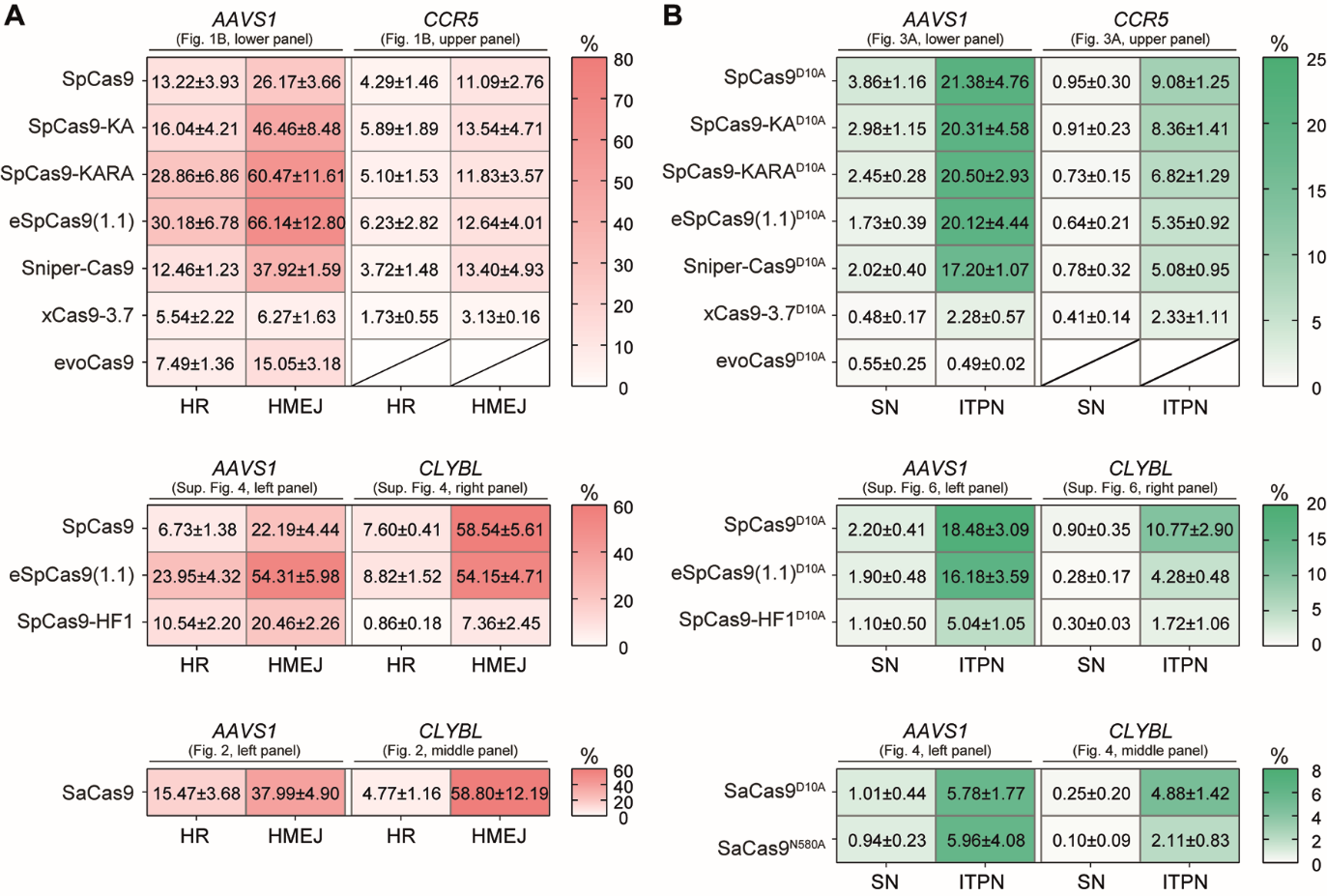


**Supplementary Figure S11.** **Summary of genome editing efficiencies achieved with the strategies investigated in this study.** Heatmaps comparing genome editing efficiencies reached at safe harbour loci with (**A**) “classic” homologous recombination (HR) and homology-mediated end joining (HMEJ) based on parental Cas9 nucleases versus their high-specificity Cas9 derivatives; and (**B**) single nicking (SN) and *in trans* paired nicking (ITPN) based on parental Cas9^D10A^ nickases versus their high-specificity Cas9^D10A^ derivates.


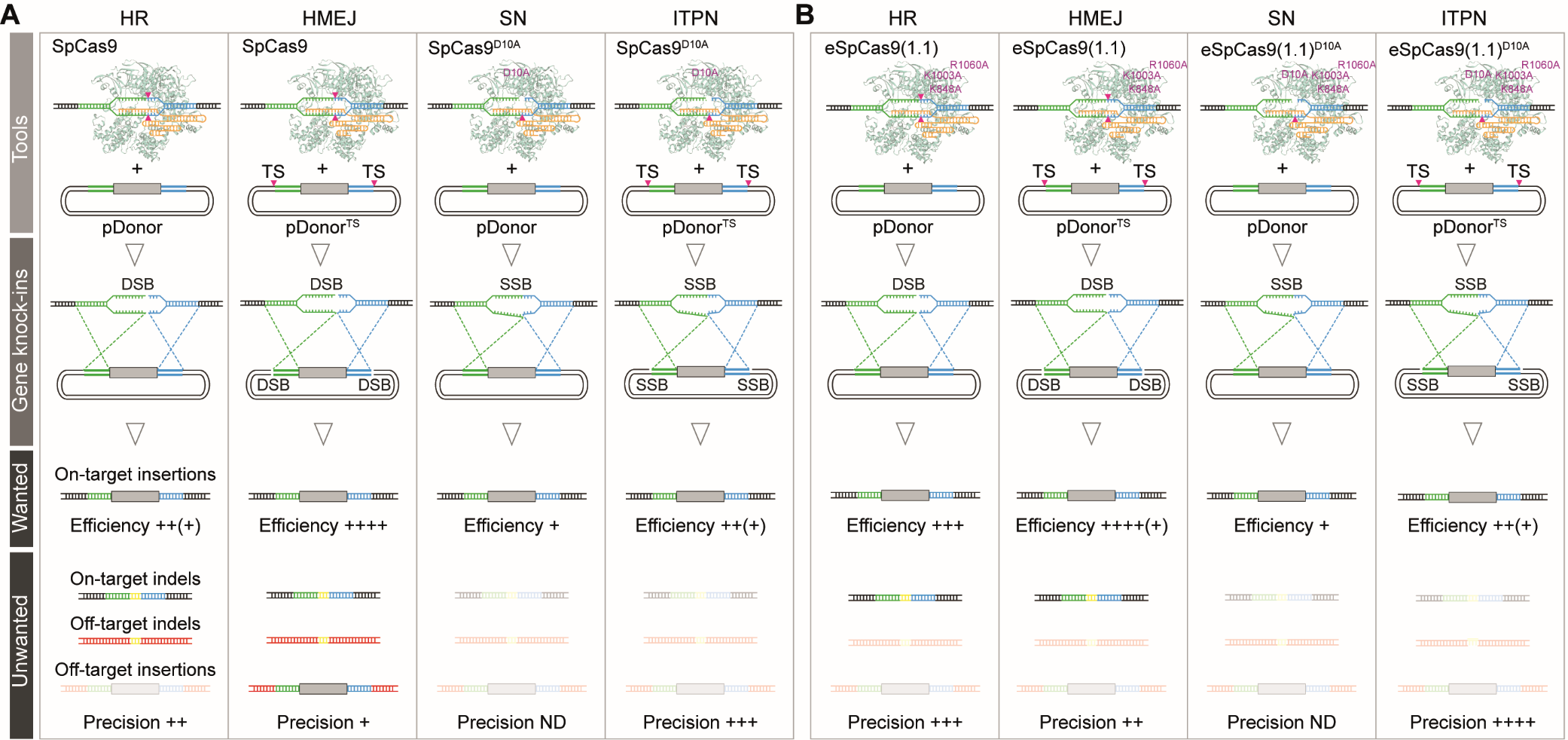


**Supplementary Figure S12.** **Schematics of the genome editing strategies and outcomes investigated in this study.** Genome editing strategies based on (**A**) conventional Cas9 nucleases or nickases and (**B**) high-specificity Cas9 nucleases or nickases. The relative frequencies of wanted and unwanted genome editing outcomes resulting from the indicated approaches are marked by + signs. HR, canonical homologous recombination; HMEJ, homologous-mediated end joining; SN, single nicking; ITPN, in trans paired nicking; DSB, double-strand DNA brake; SSB, single-strand DNA break; TS, target site; N.D., not determined.
