# Supplementary material for: Precise homology-directed installation of large genomic edits in human cells with cleaving and nicking high-specificity Cas9 variants
Source: Nucleic Acids Res. 2023 Mar 17;51(7):3465–84. doi: 10.1093/nar/gkad165 (PMC10123109; doi:10.1093/nar/gkad165)
Supplement: gkad165_Supplemental_Files [file gkad165_supplemental_files.zip › Wang et al. Supplementary Tables S1-S36.docx]

**Supplementary Table S1.** Target sequences of *S. pyogenes* gRNAs and *S. aureus* Sa-gRNAs used in this study.

| Plasmid name | Target sequence (5' 🡪 3') |
| --- | --- |
| AY27_ pU6.gRNA^CLYBL^ | TCCTACATACCGTTATCCTGTGG |
| AP65_pU6.Sa-gRNA^SI^ | CCACAGTGGGGCCACTAGGGACAGGAT |
| AM77_pU6.Sa-gRNA^CLYBL^ | CCTACATACCGTTATCCTGTGGTAGAGT |
| AG65_pU6.gRNA^VEGFA^ | GGTGAGTGAGTGTGTGCGTGTGG |
| AG66_pU6.gRNA^CALM2^ | GATGGTCAAGTAAACTATGAAGG |

**Note:** Protospacers and PAM sites are highlighted in black and red, respectively.

**Supplementary Table S2.**  Transfection scheme for testing DSB-dependent genome editing using regular versus high-specificity SpCas9 nucleases at *CCR5* (**Figure 1B**, top panel).

| HeLa cells | 5.0 ×10^4^ cells per well of 24-well plates | | | | | | | | | |
| --- | --- | --- | --- | --- | --- | --- | --- | --- | --- | --- |
|  | 400 ng total DNA and 1.54 µl PEI (1 mg ml^-1^) per well (medium replaced at 6 h post-transfection | | | | | | | | | |
| Reagents | SpCas9 | SpCas9-KA | SpCas9-KARA | eSpCas9(1.1) | Sniper-Cas9 | xCas9-3.7 | pmC.Donor^R5^ | pmC.Donor^R5.TS^ | gRNA^R5^ | gRNA^Empty^ |
| Plasmid codes | AV62 | AP75 | AP69 | AW01 | AE69 | AT84 | BB43 | BB44 | AY22 | AS10 |
| Construct size (bp) | 9215 | 9360 | 9360 | 9360 | 9217 | 9217 | 6638 | 6832 | 3046 | 3915 |
| 1 | 198.6 |  |  |  |  |  | 138.0 |  | 63.3 |  |
| 2 | 198.6 |  |  |  |  |  |  | 138.0 | 63.3 |  |
| 3 | 198.6 |  |  |  |  |  | 138.0 |  |  | 63.3 |
| 4 |  | 198.6 |  |  |  |  | 138.0 |  | 63.3 |  |
| 5 |  | 198.6 |  |  |  |  |  | 138.0 | 63.3 |  |
| 6 |  | 198.6 |  |  |  |  | 138.0 |  |  | 63.3 |
| 7 |  |  | 198.6 |  |  |  | 138.0 |  | 63.3 |  |
| 8 |  |  | 198.6 |  |  |  |  | 138.0 | 63.3 |  |
| 9 |  |  | 198.6 |  |  |  | 138.0 |  |  | 63.3 |
| 10 |  |  |  | 198.6 |  |  | 138.0 |  | 63.3 |  |
| 11 |  |  |  | 198.6 |  |  |  | 138.0 | 63.3 |  |
| 12 |  |  |  | 198.6 |  |  | 138.0 |  |  | 63.3 |
| 13 |  |  |  |  | 198.6 |  | 138.0 |  | 63.3 |  |
| 14 |  |  |  |  | 198.6 |  |  | 138.0 | 63.3 |  |
| 15 |  |  |  |  | 198.6 |  | 138.0 |  |  | 63.3 |
| 16 |  |  |  |  |  | 198.6 | 138.0 |  | 63.3 |  |
| 17 |  |  |  |  |  | 198.6 |  | 138.0 | 63.3 |  |
| 18 |  |  |  |  |  | 198.6 | 138.0 |  |  | 63.3 |

**Supplementary Table S3.**  Transfection scheme for testing DSB-dependent genome editing using regular versus high-specificity SpCas9 nucleases at *AAVS1* (**Figure 1B**, bottom panel).

| HeLa cells | 5.0 ×10^4^ cells per well of 24-well plates | | | | | | | | | | |
| --- | --- | --- | --- | --- | --- | --- | --- | --- | --- | --- | --- |
|  | 400 ng total DNA and 1.54 µl PEI (1 mg ml^-1^) per well (medium replaced at 6 h post-transfection) | | | | | | | | | | |
| Reagents | SpCas9 | SpCas9-KA | SpCas9-KARA | eSpCas9(1.1) | Sniper-Cas9 | xCas9-3.7 | evoCas9 | pE.Donor^S1^ | pE.Donor^S1.TS^ | gRNA^S1^ | gRNA^Empty^ |
| Plasmid codes | AV62 | AP75 | AP69 | AW01 | AE69 | AT84 | AP73 | AT13 | AA63 | AS11 | AS10 |
| Construct size (bp) | 9215 | 9360 | 9360 | 9360 | 9217 | 9217 | 9215 | 7199 | 7359 | 3973 | 3915 |
| 1 | 184.4 |  |  |  |  |  |  | 138.9 |  | 76.7 |  |
| 2 | 184.4 |  |  |  |  |  |  |  | 138.9 | 76.7 |  |
| 3 | 184.4 |  |  |  |  |  |  | 138.9 |  |  | 76.7 |
| 4 |  | 184.4 |  |  |  |  |  | 138.9 |  | 76.7 |  |
| 5 |  | 184.4 |  |  |  |  |  |  | 138.9 | 76.7 |  |
| 6 |  | 184.4 |  |  |  |  |  | 138.9 |  |  | 76.7 |
| 7 |  |  | 184.4 |  |  |  |  | 138.9 |  | 76.7 |  |
| 8 |  |  | 184.4 |  |  |  |  |  | 138.9 | 76.7 |  |
| 9 |  |  | 184.4 |  |  |  |  | 138.9 |  |  | 76.7 |
| 10 |  |  |  | 184.4 |  |  |  | 138.9 |  | 76.7 |  |
| 11 |  |  |  | 184.4 |  |  |  |  | 138.9 | 76.7 |  |
| 12 |  |  |  | 184.4 |  |  |  | 138.9 |  |  | 76.7 |
| 13 |  |  |  |  | 184.4 |  |  | 138.9 |  | 76.7 |  |
| 14 |  |  |  |  | 184.4 |  |  |  | 138.9 | 76.7 |  |
| 15 |  |  |  |  | 184.4 |  |  | 138.9 |  |  | 76.7 |
| 16 |  |  |  |  |  | 184.4 |  | 138.9 |  | 76.7 |  |
| 17 |  |  |  |  |  | 184.4 |  |  | 138.9 | 76.7 |  |
| 18 |  |  |  |  |  | 184.4 |  | 138.9 |  |  | 76.7 |
| 19 |  |  |  |  |  |  | 184.4 | 138.9 |  | 76.7 |  |
| 20 |  |  |  |  |  |  | 184.4 |  | 138.9 | 76.7 |  |
| 21 |  |  |  |  |  |  | 184.4 | 138.9 |  |  | 76.7 |

**Supplementary Table S4.**  Transfection scheme for comparing DSB-dependent genome editing using eSpCas9(1.1) versus SpCas9-HF1 at *AAVS1* (**Supplementary Figure 4**, left panel).

| HeLa cells | 4.0 ×10^4^ cells per well of 24-well plates | | | | | | |
| --- | --- | --- | --- | --- | --- | --- | --- |
|  | 400 ng total DNA and 1.75 µl PEI (1 mg ml^-1^) per well (medium replaced at 6 h post-transfection) | | | | | | |
| Reagents | SpCas9 | eSpCas9(1.1) | SpCas9-HF1 | pE.Donor^S1^ | pE.Donor^S1.TS^ | gRNA^S1^ | gRNA^Empty^ |
| Plasmid codes | AU26 | AV50 | AV64 | AT13 | AA63 | AS11 | AS10 |
| Construct size (bp) | 10636 | 8506 | 9300 | 7199 | 7359 | 3973 | 3915 |
| 1 | 195.1 |  |  | 132.0 |  | 72.9 |  |
| 2 | 195.1 |  |  |  | 132.0 | 72.9 |  |
| 3 | 195.1 |  |  | 132.0 |  |  | 72.9 |
| 4 |  | 195.1 |  | 132.0 |  | 72.9 |  |
| 5 |  | 195.1 |  |  | 132.0 | 72.9 |  |
| 6 |  | 195.1 |  | 132.0 |  |  | 72.9 |
| 7 |  |  | 195.1 | 132.0 |  | 72.9 |  |
| 8 |  |  | 195.1 |  | 132.0 | 72.9 |  |
| 9 |  |  | 195.1 | 132.0 |  |  | 72.9 |

**Supplementary Table S5.** Transfection scheme for comparing DSB-dependent genome editing using eSpCas9(1.1) versus SpCas9-HF1 at *CLYBL* (**Supplementary Figure 4**, right panel).

| HeLa cells | 5.0 ×10^4^ cells per well of 24-well plates | | | | | | |
| --- | --- | --- | --- | --- | --- | --- | --- |
|  | 600 ng total DNA and 2.19 µl PEI (1 mg ml^-1^) per well (medium replaced at 6 h post-transfection) | | | | | | |
| Reagents | SpCas9 | eSpCas9(1.1) | SpCas9-HF1 | pDonor^CLYBL^ | pDonor^CLYBL.TS^ | gRNA^CLYBL^ | gRNA^Empty^ |
| Plasmid codes | AU26 | AV50 | AV64 | BA02 | AZ64 | AY27 | AS10 |
| Construct size (bp) | 10636 | 8506 | 9300 | 5088 | 5217 | 3047 | 3915 |
| 1 | 340.0 |  |  | 162.6 |  | 97.4 |  |
| 2 | 340.0 |  |  |  | 162.6 | 97.4 |  |
| 3 | 340.0 |  |  | 162.6 |  |  | 97.4 |
| 4 |  | 340.0 |  | 162.6 |  | 97.4 |  |
| 5 |  | 340.0 |  |  | 162.6 | 97.4 |  |
| 6 |  | 340.0 |  | 162.6 |  |  | 97.4 |
| 7 |  |  | 340.0 | 162.6 |  | 97.4 |  |
| 8 |  |  | 340.0 |  | 162.6 | 97.4 |  |
| 9 |  |  | 340.0 | 162.6 |  |  | 97.4 |

**Supplementary Table S6.**  Transfection scheme for assessing HDR-mediated restriction site knock-ins at *AAVS1* using high-specificity SpCas9 nucleases (**Figure 1C**).

| HeLa cells | 7.0 ×10^4^ cells per well of 24-well plates | | | | | | | |
| --- | --- | --- | --- | --- | --- | --- | --- | --- |
|  | 400 ng total DNA and 1.54 µl PEI (1 mg ml^-1^) per well (medium replaced at 6 h post-transfection) | | | | | | | |
| Reagents | SpCas9 | SpCas9-KA | SpCas9-KARA | eSpCas9(1.1) | Sniper-Cas9 | pS.Donor^S1^ | pS.Donor^S1.TS^ | gRNA^S1^ |
| Plasmid codes | AV62 | AP75 | AP69 | AW01 | AE69 | AX44 | AX53 | AS11 |
| Construct size (bp) | 9215 | 9360 | 9360 | 9360 | 9217 | 3333 | 3526 | 3046 |
| 1 | 223.1 |  |  |  |  | 80.7 |  | 96.2 |
| 2 | 223.1 |  |  |  |  |  | 80.7 | 96.2 |
| 3 |  | 223.1 |  |  |  | 80.7 |  | 96.2 |
| 4 |  | 223.1 |  |  |  |  | 80.7 | 96.2 |
| 5 |  |  | 223.1 |  |  | 80.7 |  | 96.2 |
| 6 |  |  | 223.1 |  |  |  | 80.7 | 96.2 |
| 7 |  |  |  | 223.1 |  | 80.7 |  | 96.2 |
| 8 |  |  |  | 223.1 |  |  | 80.7 | 96.2 |
| 9 |  |  |  |  | 223.1 | 80.7 |  | 96.2 |
| 10 |  |  |  |  | 223.1 |  | 80.7 | 96.2 |

**Supplementary Table S7.**  Transfection scheme for testing HDR-mediated transgene knock-ins at *AAVS1* in iPSCs using eSpCas9(1.1) (**Figure 1D**).

| iPSCs | 3.0 ×10^4^ cells per well of 24-well plates | | | | |
| --- | --- | --- | --- | --- | --- |
|  | 500 ng total DNA and 1.50 µl Lipofectamine Stem per well (medium replaced at 24 h post-transfection) | | | | |
| Reagents | eSpCas9(1.1) | pEP.Donor^S1^ | pEP.Donor^S1.TS^ | gRNA^S1^ | gRNA^Empty^ |
| Plasmid codes | AW01 | AV11 | AV09 | AS11 | AS10 |
| Construct size (bp) | 9360 | 7881 | 8041 | 3046 | 3915 |
| 1 | 227.9 | 196.8 |  | 75.3 |  |
| 2 | 227.9 |  | 196.8 | 75.3 |  |
| 3 | 227.9 | 196.8 |  |  | 75.3 |

**Supplementary Table S8.** Transfection scheme for comparing DSB-dependent genome editing strategies using orthogonal SaCas9 complexes at *AAVS1* (**Figure 2**).

| HeLa cells | 4.0 ×10^4^ cells per well of 24-well plates | | | | |
| --- | --- | --- | --- | --- | --- |
|  | 250 ng total DNA and 1.10 µl PEI (1 mg ml^-1^) per well (medium replaced at 6 h post-transfection) | | | | |
| Reagents | SaCas9 | pE.Donor^S1^ | pE.Donor^S1.TS^ | Sa.gRNA^S1^ | Sa.gRNA^Empty^ |
| Plasmid codes | BA15 | AT13 | AA63 | AP65 | AZ46 |
| Construct size (bp) | 8319 | 7199 | 7359 | 2289 | 2288 |
| 1 | 117.2 | 100.7 |  | 32.0 |  |
| 2 | 117.2 |  | 100.7 | 32.0 |  |
| 3 | 117.2 | 100.7 |  |  | 32.0 |

**Supplementary Table S9.** Transfection scheme for comparing DSB-dependent genome editing strategies using orthogonal SaCas9 complexes at *CLYBL* in HeLa cells (**Figure 2**).

| HeLa cells | 4.0 ×10^4^ cells per well of 24-well plates | | | | |
| --- | --- | --- | --- | --- | --- |
|  | 250 ng total DNA and 1.10 µl PEI (1 mg ml^-1^) per well (medium replaced at 6 h post-transfection) | | | | |
| Reagents | SaCas9 | pDonor^CLYBL^ | pDonor^CLYBL.TS^ | Sa-gRNA^CLYBL^ | Sa-gRNA^Empty^ |
| Plasmid codes | BA15 | BA02 | AZ64 | AM77 | AZ46 |
| Construct size (bp) | 8319 | 5088 | 5217 | 2290 | 2288 |
| 1 | 132.9 | 80.7 |  | 36.3 |  |
| 2 | 132.9 |  | 100.7 | 36.3 |  |
| 3 | 132.9 | 80.7 |  |  | 36.3 |

**Supplementary Table S10.**  Transfection scheme for comparing DSB-dependent genome editing strategies using orthogonal SaCas9 complexes at *CLYBL* in iPSCs (**Figure 2**).

| iPSCs | 3.0 ×10^4^ cells per well of 24-well plates | | | | |
| --- | --- | --- | --- | --- | --- |
|  | 500 ng total DNA and 1.50 µl Lipofectamine Stem per well (medium replaced at 24 h post-transfection) | | | | |
| Reagents | SaCas9 | pDonor^CLYBL^ | pDonor^CLYBL.TS^ | Sa-gRNA^CLYBL^ | Sa-gRNA^Empty^ |
| Plasmid codes | BA15 | AD60 | AD59 | AM77 | AZ46 |
| Construct size (bp) | 8319 | 5770 | 5899 | 2290 | 2288 |
| 1 | 254.9 | 175.5 |  | 69.7 |  |
| 2 | 254.9 |  | 175.5 | 69.7 |  |
| 3 | 254.9 | 175.5 |  |  | 69.7 |

**Supplementary Table S11.**  Transfection scheme for testing SSB-dependent genome editing using regular versus high-specificity SpCas9^D10A^ nickases at *CCR5* (**Figure 3A**, top panel).

| HeLa cells | 5.0 ×10^4^ cells per well of 24-well plates | | | | | | | | | |
| --- | --- | --- | --- | --- | --- | --- | --- | --- | --- | --- |
|  | 400 ng total DNA and 1.54 µl PEI (1 mg ml^-1^) per well (medium replaced at 6 h post-transfection) | | | | | | | | | |
| Reagents | SpCas9^D10A^ | SpCas9-KA^D10A^ | SpCas9-KARA^D10A^ | eSpCas9(1.1) ^D10A^ | Sniper-Cas9 ^D10A^ | xCas9-3.7 ^D10A^ | pmC.Donor^R5^ | pmC.Donor^R5.TS^ | gRNA^R5^ | gRNA^Empty^ |
| Plasmid codes | AB65 | AP76 | AP70 | AA69 | AE70 | AT85 | BB43 | BB44 | AY22 | AS10 |
| Construct size (bp) | 9215 | 9360 | 9360 | 9360 | 9217 | 9217 | 6638 | 6832 | 3046 | 3915 |
| 1 | 198.6 |  |  |  |  |  | 138.0 |  | 63.3 |  |
| 2 | 198.6 |  |  |  |  |  |  | 138.0 | 63.3 |  |
| 3 | 198.6 |  |  |  |  |  | 138.0 |  |  | 63.3 |
| 4 |  | 198.6 |  |  |  |  | 138.0 |  | 63.3 |  |
| 5 |  | 198.6 |  |  |  |  |  | 138.0 | 63.3 |  |
| 6 |  | 198.6 |  |  |  |  | 138.0 |  |  | 63.3 |
| 7 |  |  | 198.6 |  |  |  | 138.0 |  | 63.3 |  |
| 8 |  |  | 198.6 |  |  |  |  | 138.0 | 63.3 |  |
| 9 |  |  | 198.6 |  |  |  | 138.0 |  |  | 63.3 |
| 10 |  |  |  | 198.6 |  |  | 138.0 |  | 63.3 |  |
| 11 |  |  |  | 198.6 |  |  |  | 138.0 | 63.3 |  |
| 12 |  |  |  | 198.6 |  |  | 138.0 |  |  | 63.3 |
| 13 |  |  |  |  | 198.6 |  | 138.0 |  | 63.3 |  |
| 14 |  |  |  |  | 198.6 |  |  | 138.0 | 63.3 |  |
| 15 |  |  |  |  | 198.6 |  | 138.0 |  |  | 63.3 |
| 16 |  |  |  |  |  | 198.6 | 138.0 |  | 63.3 |  |
| 17 |  |  |  |  |  | 198.6 |  | 138.0 | 63.3 |  |
| 18 |  |  |  |  |  | 198.6 | 138.0 |  |  | 63.3 |

**Supplementary Table S12.**  Transfection scheme for testing SSB-dependent genome editing using regular versus high-specificity SpCas9^D10A^ nickases at *AAVS1* (**Figure 3B**, bottom panel).

| HeLa cells | 5.0 ×10^4^ cells per well of 24-well plates | | | | | | | | | | |
| --- | --- | --- | --- | --- | --- | --- | --- | --- | --- | --- | --- |
|  | 400 ng total DNA and 1.54 µl PEI (1 mg ml^-1^) per well (medium replaced at 6 h post-transfection) | | | | | | | | | | |
| Reagents | SpCas9^D10A^ | SpCas9-KA^D10A^ | SpCas9-KARA^D10A^ | eSpCas9(1.1) ^D10A^ | Sniper-Cas9 ^D10A^ | xCas9-3.7 ^D10A^ | evoCas9 ^D10A^ | pE.Donor^S1^ | pE.Donor^S1.TS^ | gRNA^S1^ | gRNA^Empty^ |
| Plasmid codes | AB65 | AP76 | AP70 | AA69 | AE70 | AT85 | AP74 | AT13 | AA63 | AS11 | AS10 |
| Construct size (bp) | 9215 | 9360 | 9360 | 9360 | 9217 | 9217 | 9215 | 7199 | 7359 | 3973 | 3915 |
| 1 | 184.4 |  |  |  |  |  |  | 138.9 |  | 76.7 |  |
| 2 | 184.4 |  |  |  |  |  |  |  | 138.9 | 76.7 |  |
| 3 | 184.4 |  |  |  |  |  |  | 138.9 |  |  | 76.7 |
| 4 |  | 184.4 |  |  |  |  |  | 138.9 |  | 76.7 |  |
| 5 |  | 184.4 |  |  |  |  |  |  | 138.9 | 76.7 |  |
| 6 |  | 184.4 |  |  |  |  |  | 138.9 |  |  | 76.7 |
| 7 |  |  | 184.4 |  |  |  |  | 138.9 |  | 76.7 |  |
| 8 |  |  | 184.4 |  |  |  |  |  | 138.9 | 76.7 |  |
| 9 |  |  | 184.4 |  |  |  |  | 138.9 |  |  | 76.7 |
| 10 |  |  |  | 184.4 |  |  |  | 138.9 |  | 76.7 |  |
| 11 |  |  |  | 184.4 |  |  |  |  | 138.9 | 76.7 |  |
| 12 |  |  |  | 184.4 |  |  |  | 138.9 |  |  | 76.7 |
| 13 |  |  |  |  | 184.4 |  |  | 138.9 |  | 76.7 |  |
| 14 |  |  |  |  | 184.4 |  |  |  | 138.9 | 76.7 |  |
| 15 |  |  |  |  | 184.4 |  |  | 138.9 |  |  | 76.7 |
| 16 |  |  |  |  |  | 184.4 |  | 138.9 |  | 76.7 |  |
| 17 |  |  |  |  |  | 184.4 |  |  | 138.9 | 76.7 |  |
| 18 |  |  |  |  |  | 184.4 |  | 138.9 |  |  | 76.7 |
| 19 |  |  |  |  |  |  | 184.4 | 138.9 |  | 76.7 |  |
| 20 |  |  |  |  |  |  | 184.4 |  | 138.9 | 76.7 |  |
| 21 |  |  |  |  |  |  | 184.4 | 138.9 |  |  | 76.7 |

**Supplementary Table S13.**  Transfection scheme for comparing DSB-dependent genome editing using eSpCas9(1.1) ^D10A^ versus SpCas9-HF1^D10A^ at *AAVS1* (**Supplementary Figure 6**, left panel).

| HeLa cells | 4.0 ×10^4^ cells per well of 24-well plates | | | | | | |
| --- | --- | --- | --- | --- | --- | --- | --- |
|  | 400 ng total DNA and 1.75 µl PEI (1 mg ml^-1^) per well (medium replaced at 6 h post-transfection) | | | | | | |
| Reagents | SpCas9^D10A^ | eSpCas9(1.1) ^D10A^ | SpCas9-HF ^D10A^ | pE.Donor^S1^ | pE.Donor^S1.TS^ | gRNA^S1^ | gRNA^Empty^ |
| Plasmid codes | AU28 | BB36 | BB37 | AT13 | AA63 | AS11 | AS10 |
| Construct size (bp) | 10636 | 8506 | 9300 | 7199 | 7359 | 3973 | 3915 |
| 1 | 195.1 |  |  | 132.0 |  | 72.9 |  |
| 2 | 195.1 |  |  |  | 132.0 | 72.9 |  |
| 3 | 195.1 |  |  | 132.0 |  |  | 72.9 |
| 4 |  | 195.1 |  | 132.0 |  | 72.9 |  |
| 5 |  | 195.1 |  |  | 132.0 | 72.9 |  |
| 6 |  | 195.1 |  | 132.0 |  |  | 72.9 |
| 7 |  |  | 195.1 | 132.0 |  | 72.9 |  |
| 8 |  |  | 195.1 |  | 132.0 | 72.9 |  |
| 9 |  |  | 195.1 | 132.0 |  |  | 72.9 |

**Supplementary Table S14.** Transfection scheme for comparing DSB-dependent genome editing using eSpCas9(1.1)^D10A^ versus SpCas9-HF1^D10A^ at *CLYBL* (**Supplementary Figure 6**, right panel).

| HeLa cells | 5.0 ×10^4^ cells per well of 24-well plates | | | | | | |
| --- | --- | --- | --- | --- | --- | --- | --- |
|  | 600 ng total DNA and 2.19 µl PEI (1 mg ml^-1^) per well (medium replaced at 6 h post-transfection) | | | | | | |
| Reagents | SpCas9^D10A^ | eSpCas9(1.1) ^D10A^ | SpCas9-HF ^D10A^ | pDonor^CLYBL^ | pDonor^CLYBL.TS^ | gRNA^CLYBL^ | gRNA^Empty^ |
| Plasmid codes | AU28 | BB36 | BB37 | BA02 | AZ64 | AY27 | AS10 |
| Construct size (bp) | 10636 | 8506 | 9300 | 5088 | 5217 | 3047 | 3915 |
| 1 | 340.0 |  |  | 162.6 |  | 97.4 |  |
| 2 | 340.0 |  |  |  | 162.6 | 97.4 |  |
| 3 | 340.0 |  |  | 162.6 |  |  | 97.4 |
| 4 |  | 340.0 |  | 162.6 |  | 97.4 |  |
| 5 |  | 340.0 |  |  | 162.6 | 97.4 |  |
| 6 |  | 340.0 |  | 162.6 |  |  | 97.4 |
| 7 |  |  | 340.0 | 162.6 |  | 97.4 |  |
| 8 |  |  | 340.0 |  | 162.6 | 97.4 |  |
| 9 |  |  | 340.0 | 162.6 |  |  | 97.4 |

**Supplementary Table S15.** Transfection scheme for testing SpCas9- and eSpCas9^D10A^-triggered gene targeting at *AAVS1* in iPSCs (**Figure 3C**).

| iPSCs | 3.0 ×10^4^ cells per well of 24-well plates | | | | |
| --- | --- | --- | --- | --- | --- |
|  | 500 ng total DNA and 1.50 µl Lipofectamine Stem per well (medium replaced at 24 h post-transfection) | | | | |
| Encoded products | SpCas9 | eSpCas9(1.1)^D10A^ | pEP.Donor^S1^ | pEP.Donor^S1.TS^ | gRNA^S1^ |
| Plasmid codes | AV62 | AA69 | AV11 | AV09 | AS11 |
| Construct size (bp) | 9215 | 9360 | 7881 | 8041 | 3046 |
| 1 | 227.9 |  | 196.8 |  | 75.3 |
| 2 |  | 227.9 | 196.8 |  | 75.3 |
| 3 |  | 227.9 |  | 196.8 | 75.3 |

**Supplementary Table S16.** Transfection scheme for comparing SSB-dependent genome editing strategies using orthogonal SaCas9 nickases at *AAVS1* (**Figure 4**).

| HeLa cells | 4.0 ×10^4^ cells per well of 24-well plates | | | | |
| --- | --- | --- | --- | --- | --- |
|  | 250 ng DNA and 1.10 µl PEI (1 mg ml^-1^) per well ( medium replaced at 6 h post-transfection) | | | | |
| Reagents | SaCas9^D10A^ | SaCas9^N580A^ | pE.Donor^S1^ | pE.Donor^S1.TS^ | Sa.gRNA^S1^ |
| Plasmid codes | BA31 | BA32 | AT13 | AA63 | AP65 |
| Construct size (bp) | 8319 | 8319 | 7199 | 7359 | 2289 |
| 1 | 117.2 |  | 100.7 |  | 32.0 |
| 2 | 117.2 |  |  | 100.7 | 32.0 |
| 3 |  | 117.2 | 100.7 |  | 32.0 |
| 4 |  | 117.2 |  | 100.7 | 32.0 |

**Supplementary Table S17.** Transfection scheme for comparing SSB-dependent genome editing strategies using orthogonal SaCas9 nickases at *CLYBL* (**Figure 4**).

| HeLa cells | 4.0 ×10^4^ cells per well of 24-well plates | | | | |
| --- | --- | --- | --- | --- | --- |
|  | 250 ng total DNA and 1.10 µl PEI (1 mg ml^-1^) per well (medium replaced at 6 h post-transfection) | | | | |
| Reagents | SaCas9^D10A^ | SaCas9^N580A^ | pDonor^CLYBL^ | pDonor^CLYBL.TS^ | Sa.gRNA^CLYBL^ |
| Plasmid codes | BA31 | BA32 | BA02 | AZ64 | AM77 |
| Construct size (bp) | 8319 | 8319 | 5088 | 5217 | 2290 |
| 1 | 132.9 |  | 80.7 |  | 36.3 |
| 2 | 132.9 |  |  | 80.7 | 36.3 |
| 3 |  | 132.9 | 80.7 |  | 36.3 |
| 4 |  | 132.9 |  | 80.7 | 36.3 |

**Supplementary Table S18.** Transfection scheme for comparing SSB-dependent genome editing strategies using orthogonal SaCas9 nickases at *CLYBL* in iPSCs (**Figure 4**).

| iPSCs | 3.0 ×10^4^ cells per well of 24-well plates | | | | |
| --- | --- | --- | --- | --- | --- |
|  | 500 ng total DNA and 1.50 µl Lipofectamine Stem per well (medium replaced at 24 h post-transfection) | | | | |
| Reagents | SaCas9^D10A^ | SaCas9^N580A^ | Donor^CLYBL^ | Donor^CLYBL.TS^ | Sa.gRNA^CLYBL^ |
| Plasmid codes | BA31 | BA32 | AD60 | AD59 | AM77 |
| Construct size (bp) | 8319 | 8319 | 5770 | 5899 | 2290 |
| 1 | 254.9 |  | 175.5 |  | 69.7 |
| 2 | 254.9 |  |  | 175.5 | 69.7 |
| 3 |  | 254.9 | 175.5 |  | 69.7 |
| 4 |  | 254.9 |  | 175.5 | 69.7 |

**Supplementary Table S19.** Transfection scheme for assessing mutagenic loads in cells edited through canonical homologous recombination versus in trans paired nicking (**Figure 5**).

| HeLa cells | 5.0 ×10^4^ cells per well of 24-well plates | | | | | | | | | |
| --- | --- | --- | --- | --- | --- | --- | --- | --- | --- | --- |
|  | 500 ng total DNA and 1.54 µl PEI (1 mg ml^-1^) per well (medium replaced at 6 h post-transfection) | | | | | | | | | |
| Reagents | SpCas9 | SpCas9^D10A^ | eSpCas9(1.1) | eSpCas9(1.1) ^D10A^ | Sniper-Cas9 | Sniper-Cas9 ^D10A^ | pEP.Donor^S1^ | pEP.Donor^S1.TS^ | gRNA^S1^ | gRNA^Empty^ |
| Plasmid codes | AV62 | AB65 | AW01 | AA69 | AE69 | AE70 | AV11 | AV09 | AS11 | AS10 |
| Construct size (bp) | 9215 | 9215 | 9360 | 9360 | 9217 | 9217 | 7881 | 8041 | 3046 | 3915 |
| 1 | 218.7 |  |  |  |  |  | 187.0 |  | 94.3 |  |
| 2 |  | 218.7 |  |  |  |  |  | 187.0 | 94.3 |  |
| 3 | 218.7 |  |  |  |  |  | 187.0 |  |  | 94.3 |
| 4 |  |  | 218.7 |  |  |  | 187.0 |  | 94.3 |  |
| 5 |  |  |  | 218.7 |  |  |  | 187.0 | 94.3 |  |
| 6 |  |  | 218.7 |  |  |  | 187.0 |  |  | 94.3 |
| 7 |  |  |  |  | 218.7 |  | 187.0 |  | 94.3 |  |
| 8 |  |  |  |  |  | 218.7 |  | 187.0 | 94.3 |  |
| 9 |  |  |  |  | 218.7 |  | 187.0 |  |  | 94.3 |

**Supplementary Table S20.** Transfection scheme for assessing off-target chromosomal donor DNA insertions resulting from HR, HMEJ and ITPN (**Figure 6**).

| HeLa cells | 8.0 ×10^4^ cells per well of 24-well plates | | | | | | |
| --- | --- | --- | --- | --- | --- | --- | --- |
|  | 500 ng total DNA and 1.54 µl PEI (1 mg ml^-1^) per well (medium replaced at 6 h post-transfection) | | | | | | |
| Reagents | SpCas9 | SpCas9^D10A^ | eSpCas9(1.1) | eSpCas9(1.1) ^D10A^ | pEP.Donor^S1^ | pEP.Donor^S1.TS^ | gRNA^S1^ |
| Plasmid codes | AV62 | AB65 | AW01 | AA69 | AV11 | AV09 | AS11 |
| Construct size (bp) | 9215 | 9215 | 9360 | 9360 | 7881 | 8041 | 3046 |
| 1 | 218.7 |  |  |  | 187.0 |  | 94.3 |
| 2 | 218.7 |  |  |  |  | 187.0 | 94.3 |
| 3 |  | 218.7 |  |  |  | 187.0 | 94.3 |
| 4 |  |  | 218.7 |  | 187.0 |  | 94.3 |
| 5 |  |  | 218.7 |  |  | 187.0 | 94.3 |
| 6 |  |  |  | 218.7 |  | 187.0 | 94.3 |

**Supplementary Table S21.** Transfection scheme for investigating the activation of p53-dependent DNA damage responses in iPSCs exposed to nucleases versus nickases (**Figure 8**).

| iPSCs | 4.0 ×10^4^ cells per well of 24-well plates | | | | | |
| --- | --- | --- | --- | --- | --- | --- |
|  | 500 ng total DNA and 1.50 µl Lipofectamine Stem per well (medium replaced at 24 h post-transfection) | | | | | |
| Reagents | SpCas9 | SpCas9^D10A^ | eSpCas9(1.1) | eSpCas9(1.1) ^D10A^ | gRNA^VEGFA^ | gRNA^CALM2^ |
| Plasmid codes | AV62 | AB65 | AW01 | AA69 | AG65 | AG66 |
| Construct size (bp) | 9215 | 9215 | 9360 | 9360 | 3056 | 3056 |
| 1 | 375.5 |  |  |  | 124.5 |  |
| 2 |  | 375.5 |  |  | 124.5 |  |
| 3 | 375.5 |  |  |  |  | 124.5 |
| 4 |  | 375.5 |  |  |  | 124.5 |
| 5 |  |  | 375.5 |  | 124.5 |  |
| 6 |  |  |  | 375.5 | 124.5 |  |
| 7 |  |  | 375.5 |  |  | 124.5 |
| 8 |  |  |  | 375.5 |  | 124.5 |

**Supplementary Table S22.** Transfection scheme for testing DSB- versus SSB-dependent genome editing strategies at *OCT4* using high-specificity CRISPR complexes in iPSCs (**Figure 9**).

| iPSCs | 3.0 ×10^4^ cells per well of 24-well plates | | | | |
| --- | --- | --- | --- | --- | --- |
|  | 500 ng DNA and 1.50 µl Lipofectamine Stem per well (medium replaced at 24 h post-transfection) | | | | |
| Reagents | eSpCas9(1.1) | eSpCas9(1.1)^D10A^ | PDonor^OCT4^ | pDonor^OCT4.TS^ | gRNA^OCT4.1^ |
| Plasmid codes | AW01 | AA69 | AX24 | AX74 | AX33 |
| Construct size (bp) | 9360 | 9360 | 7955 | 8075 | 3046 |
| 1 | 227.9 |  | 196.8 |  | 75.3 |
| 2 | 227.9 |  |  | 196.8 | 75.3 |
| 3 |  | 227.9 |  | 196.8 | 75.3 |

**Supplementary Table S23**. Primer pairs and PCR mixtures used for *AAVS1* amplification (**Figures 1C** and **3C**).

| Target | Primer code | Primers (5’ → 3’) / final concentrations (µM) | dNTP  (mM) | MgCl_2_  (mM) | GoTaq Flexi Buffer | GoTaq G2 Flexi DNA Polymerase ( U µl^-1^) | Amplicon size  (bp) |
| --- | --- | --- | --- | --- | --- | --- | --- |
| *AAVS1*  (RFLA assay; **Figure 1C**) | #1257 | AGCCACCTCTCCATCCTCTT / 0.2 | 0.4 | 1 | 1× | 0.05 | 895 |
|  | #1258 | AGGGAGTTTTCCACACGGAC / 0.2 |  |  |  |  |  |
| *AAVS1*  (TIDE assay; **Figure 3D**) | #999 | TTCGGGTCACCTCTCACTCC / 0.2 | 0.4 | 1 | 1× | 0.05 | 469 |
|  | #1000 | GGCTCCATCGTAAGCAAACC / 0.2 |  |  |  |  |  |

**Supplementary Table S24**. PCR cycling parameters used for *AAVS1* amplification (**Figures 1C** and **3C**).

| Target | Initial denaturation | Denaturation | Annealing | Elongation | Cycles | Final elongation |
| --- | --- | --- | --- | --- | --- | --- |
| *AAVS1*  (RFLA assay; **Figure 1C**) | 95 ℃ | 95 ℃ | 58.3 ℃ | 72 ℃ | 40 | 72 ℃ |
|  | 5 min | 30 sec | 30 sec | 1 min |  | 5 min |
| *AAVS1*  (TIDE assay; **Figure 3D**) | 95 ℃ | 95 ℃ | 61 ℃ | 72 ℃ | 40 | 72 ℃ |
|  | 5 min | 30 sec | 30 sec | 30 sec |  | 5 min |

**Supplementary Table S25**. Primer pairs and PCR mixtures used for junction PCR analysis (**Figures 1D** and **3E**).

| Target | Primer code | Primers (5’ → 3’) / final concentrations (µM) | Mastermix  (mM) | DMSO | Amplicon size  (bp) |
| --- | --- | --- | --- | --- | --- |
| jC-*AAVS1* | #1046 | CGACAACCACTACCTGAGCA / 0.5 | 0.4 |  | 1701 |
|  | #1047 | GACCTGCCTGGAGAAGGAT / 0.5 |  |  |  |
| jT-*AAVS1* | #986 | AACCCCAACCCCGTGGAAG / 0.5 | 0.4 | 2% | 1678 |
|  | #1004 | GCACCGTCCGCTTCGAG / 0.5 |  |  |  |
| *EGFP* | #978 | CTGCATTCTAGTTGTGGTTTG / 0.5 | 0.4 |  | 596 |
|  | #979 | CTAACATGCGGTGACGTGG / 0.5 |  |  |  |

**Supplementary Table S26**. PCR cycling parameters used for junction PCR analysis (**Figures 1D** and **3E**).

| Target | Initial denaturation | Denaturation | Annealing | Elongation | Cycles | Final elongation |
| --- | --- | --- | --- | --- | --- | --- |
| jC-*AAVS1* | 98 ℃ | 98 ℃ | 64.5 ℃ | 72 ℃ | 35 | 72 ℃ |
|  | 5 min | 7 sec | 7 sec | 35 sec |  | 1 min |
| jT-*AAVS1* | 98 ℃ | 98 ℃ | 72 ℃ | | 35 | 72 ℃ |
|  | 5 min | 7 sec | 30 sec | |  | 2 min |
| *EGFP* | 98 ℃ | 98 ℃ | 72 ℃ | | 35 | 72 ℃ |
|  | 5 min | 5 sec | 20 sec | |  | 2 min |

**Supplementary Table S27**. Primer pairs and PCR mixtures for on-target and off-target DNA cleavage analysis by amplicon deep sequencing (**Figure 5**).

| Target | Primer code | Primers (5’ → 3’) / final concentrations (µM) | dNTPs  (mM) | 5× Phusion HF Buffer | Phusion HF DNA Polymerase  (U µl^-1^) | Amplicon size  (bp) |
| --- | --- | --- | --- | --- | --- | --- |
| *AAVS1*  (on-target) | #1369 | CGTGTGCTCTTCCGATCTCCCAGGGCCGGTTAATG / 0.5 | 0.2 | 1× | 0.02 | 202 |
|  | #1370 | GATGTGTATAAGAGACAGCTGCCTAACAGGAGGTGG / 0.5 |  |  |  |  |
| *CPNE5*  (off-target) | #1637 | GATGTGTATAAGAGACAGGGCTGGTCCCTGAAGACATC / 0.5 | 0.2 | 1× | 0.02 | 248 |
|  | #1639 | CGTGTGCTCTTCCGATCTCAGGGCTCTACTCACATAG / 0.5 |  |  |  |  |
| *BBOX1*  (off-target) | #1640 | GATGTGTATAAGAGACAGTAGGGGAAAGAGAGGAGCCT / 0.5 | 0.2 | 1× | 0.02 | 261 |
|  | #1641 | CGTGTGCTCTTCCGATCTCCATCCTCCTGTGGGCTAAA / 0.5 |  |  |  |  |

**Supplementary Table S28**. PCR cycling parameters for amplifying target and off-target sites of regular and high-specificity CRISPR complexes (**Figure 5**).

| Targets | Initial denaturation | Denaturation | Annealing | Elongation | Cycles | Final elongation |
| --- | --- | --- | --- | --- | --- | --- |
| *AAVS1*  (on-target) | 98 °C | 98 °C | 66 °C | 72 °C | 35 | 72 ℃ |
|  | 30 sec | 10 sec | 10 sec | 10 sec |  | 5 min |
| *CPNE5*  (off-target) | 98 °C | 98 °C | 70.3 °C | 72 °C | 35 | 72 ℃ |
|  | 30 sec | 10 sec | 10 sec | 10 sec |  | 5 min |
| *BBOX1*  (off-target) | 98 °C | 98 °C | 70.3°C | 72 °C | 35 | 72 ℃ |
|  | 30 sec | 10 sec | 10 sec | 10 sec |  | 5 min |
| Barcoding PCR step | 98 ℃ | 98 ℃ | 62.0 ℃ | 72 ℃ | 10 | 72 ℃ |
|  | 30 sec | 10 sec | 10 sec | 10 sec |  | 3 min |

**Supplementary Table S29**. Barcode PCR primers used in the targeted amplicon deep sequencing analyses (**Figure 5**).

| Primer codes | Primers (5’ → 3’) / final concentrations (µM) |
| --- | --- |
| Fun-i501 | AATGATACGGCGACCACCGAGATCTACACTAGATCGCTCGTCGGCAGCGTCAGATGTGTATAAGAGACAG / 0.25 |
| Fun-i502 | AATGATACGGCGACCACCGAGATCTACACCTCTCTATTCGTCGGCAGCGTCAGATGTGTATAAGAGACAG / 0.25 |
| Fun-i503 | AATGATACGGCGACCACCGAGATCTACACTATCCTCTTCGTCGGCAGCGTCAGATGTGTATAAGAGACAG / 0.25 |
| Fun-i504 | AATGATACGGCGACCACCGAGATCTACACAGAGTAGATCGTCGGCAGCGTCAGATGTGTATAAGAGACAG / 0.25 |
| Fun-i505 | AATGATACGGCGACCACCGAGATCTACACGTAAGGAGTCGTCGGCAGCGTCAGATGTGTATAAGAGACAG / 0.25 |
| Fun-i506 | AATGATACGGCGACCACCGAGATCTACACACTGCATATCGTCGGCAGCGTCAGATGTGTATAAGAGACAG / 0.25 |
| Fun-i701 | CAAGCAGAAGACGGCATACGAGATTCGCCTTAGTGACTGGAGTTCAGACGTGTGCTCTTCCGATCT / 0.25 |
| Fun-i702 | CAAGCAGAAGACGGCATACGAGATCTAGTACGGTGACTGGAGTTCAGACGTGTGCTCTTCCGATCT / 0.25 |
| Fun-i703 | CAAGCAGAAGACGGCATACGAGATTTCTGCCTGTGACTGGAGTTCAGACGTGTGCTCTTCCGATCT / 0.25 |
| Fun-i704 | CAAGCAGAAGACGGCATACGAGATGCTCAGGAGTGACTGGAGTTCAGACGTGTGCTCTTCCGATCT / 0.25 |
| Fun-i705 | CAAGCAGAAGACGGCATACGAGATAGGAGTCCGTGACTGGAGTTCAGACGTGTGCTCTTCCGATCT / 0.25 |

**Supplementary Table S30.** PCR mixtures used for the barcoding of deep sequencing amplicons (**Figure 5**).

| Components | Volumes | Final Concentrations |
| --- | --- | --- |
| 5× Phusion HF Buffer | 4 µl | 1× |
| dNTPs (2.5 mM each) | 1.2 µl | 0.15 mM (each) |
| PCR Grade Water | 11.6 µl | - |
| Index primer p5-XX (5 µM) | 1 µl | 0.25 µM |
| Index primer p7-XX (5 µM) | 1 µl | 0.25 µM |
| Purified PCR product | 1 µl | - |
| Phusion DNA Polymerase (2 U/µl) | 0.2 µl | 0.02 U/µl |
| Total reaction volume | 20 µl | - |

**Supplementary Table S31**. Primer pairs and PCR mixtures used for off-target insertion analysis at *CPNE5* (**Figure 6**).

| Target | Primer code | Primers (5’ → 3’) / final concentrations (µM) | dNTPs (mM) | 5× SuperFi^TM^ II Buffer | 50× Platinum^TM^ SuperFi^TM^ II DNA Polymerase | Amplicon size (bp) |
| --- | --- | --- | --- | --- | --- | --- |
| *CPNE5*  (“sense” insertion) | #2143 | AGCCCGCTTCAGAGTAACAG / 0.5 | 0.2 | 1× | 1× | 2272 |
|  | #1026 | TGCCTTGGAAAAGGCGC / 0.5 |  |  |  |  |
| *CPNE5*  (“antisense” insertion) | #2144 | GTTCACAGCTTGTAGGCCAG / 0.5 | 0.2 | 1× | 1× | 2261 |
|  | #1026 | TGCCTTGGAAAAGGCGC / 0.5 |  |  |  |  |
| jT-*AAVS1* | #1004 | GCACCGTCCGCTTCGAG / 0.5 | 0.2 | 1× | 1× | 1678 |
|  | #986 | AACCCCAACCCCGTGGAAG / 0.5 |  |  |  |  |
| jC-*AAVS1* | #1047 | GACCTGCCTGGAGAAGGAT / 0.5 | 0.2 | 1× | 1× | 1594 |
|  | #1464 | GCATGGACGAGCTGTACAAG / 0.5 |  |  |  |  |

| Target | Primer code | Primers (5’ → 3’) / final concentrations (µM) | dNTPs (mM) | GoTaq Flexi Buffer | MgCl_2_ (mM) | GoTaq G2 Flexi DNA Polymerase ( U µl^-1^) | Amplicon size (bp) |
| --- | --- | --- | --- | --- | --- | --- | --- |
| *EGFP* | #978 | CTGCATTCTAGTTGTGGTTTG / 0.5 | 0.4 | 1× | 1 | 0.05 | 596 |
|  | #979 | CTAACATGCGGTGACGTGG / 0.5 |  |  |  |  |  |

**Supplementary Table S32**. PCR cycling parameters used for off-target insertion analysis at *CPNE5* (**Figure 6**).

| Target | Initial denaturation | Denaturation | Annealing | Elongation | Cycles | Final elongation |
| --- | --- | --- | --- | --- | --- | --- |
| *CPNE5*  (“sense” insertion) | 98 ℃ | 98 ℃ | 60 ℃ | 72 ℃ | 35 | 72 ℃ |
|  | 30 sec | 10 sec | 10 sec | 90 sec |  | 5 min |
| *CPNE5*  (“antisense” insertion) | 98 ℃ | 98 ℃ | 60 ℃ | 72 ℃ | 35 | 72 ℃ |
|  | 30 sec | 10 sec | 10 sec | 90 sec |  | 5 min |
| jT-*AAVS1* | 98 ℃ | 98 ℃ | 60 ℃ | 72 ℃ | 35 | 72 ℃ |
|  | 30 sec | 10 sec | 10 sec | 90 sec |  | 5 min |
| jC-*AAVS1* | 98 ℃ | 98 ℃ | 60 ℃ | 72 ℃ | 35 | 72 ℃ |
|  | 30 sec | 10 sec | 10 sec | 90 sec |  | 5 min |
| *EGFP* | 95 ℃ | 95 ℃ | 62 ℃ | 72 ℃ | 40 | 72 ℃ |
|  | 5 min | 30 sec | 30 sec | 40 sec |  | 3 min |

**Supplementary Table S33**. Thermocycler program for generating heteroduplex substrates for the T7EI-based genotyping assays.

| Temperature | Time |
| --- | --- |
| 95 °C | 10 min |
| 95 °C to 85 °C | -2.0 °C/s |
| 85 °C | 1 min |
| 85 °C to 75 °C | -0.3 °C/s |
| 75 °C | 1 min |
| 75 °C to 65 °C | -0.3 °C/s |
| 65 °C | 1 min |
| 65 °C to 55 °C | -0.3 °C/s |
| 55 °C | 1 min |
| 55 °C to 45 °C | -0.3 °C/s |
| 45 °C | 1 min |
| 45 °C to 35 °C | -0.3 °C/s |
| 35 °C | 1 min |
| 35 °C to 25 °C | -0.3 °C/s |
| 25 °C | 1 min |
| 16 °C | Hold |

**Supplementary Table S34**. PCR mixtures used in the RT-qPCR analysis of P53 responsive and non-responsive genes (**Figures 7** and **8**).

| Targets | Primer codes | Primers (5’ → 3’) / final concentrations (µM) | SYBR Green Master mix | Amplicons  size (bp) | Target information  (Sequence accession number) |
| --- | --- | --- | --- | --- | --- |
| *P21* | #1396 | CCTCATCCCGTGTTCTCCTTT / 0.25 | 1× | 97 | NM_000389.5, NM_078467.3, NM_001291549.3, NM_001220778.2, NM_001220777.2, NM_001374509.1, NM_001374510.1, NM_001374511.1, NM_001374512.1, NM_001374513.1 |
|  | #1397 | GTACCACCCAGCGGACAAGT / 0.25 |  |  |  |
| *FAS* | #1428 | GTGACCCTTGCACCAAATGT / 0.25 | 1× | 109 | NM_000043.6, NM_152872.4, NM_001320619.2, NM_001410956.1, XM_011539766.3, XM_006717819.4, XM_011539764.3 |
|  | #1429 | AGACAAAGCCACCCCAAGTT / 0.25 |  |  |  |
| *PUMA* | #1430 | AAATTTGGCATGGGGTCTGC / 0.25 | 1× | 95 | NM_001127240.3 |
|  | #1431 | TCCCTGGGGCCACAAATCT / 0.25 |  |  |  |
| *MDM2* | #1434 | TGGGCAGCTTGAAGCAGTTG / 0.25 | 1× | 122 | NM_002392.6, NM_001145339.2, NM_001145337.3, NM_001145340.3, NM_001278462.2, NM_001367990.1, XM_047428853.1 |
|  | #1435 | CAGGCTGCCATGTGACCTAAGA / 0.25 |  |  |  |
| *P53* | #1426 | GTGGAAGGAAATTTGCGTGT / 0.25 | 1× | 184 | NM_000546.6, NM_001276761.3, NM_001276696.3, NM_001276695.3, NM_001276697.3, NM_001276698.3, NM_001276699.3, NM_001126118.2, NM_001407262.1, NM_001407265.1, NM_001407266.1, NM_001407269.1, NM_001407270.1 |
|  | #1427 | CCAGTGTGATGATGGTGAGG / 0.25 |  |  |  |
| *HPRT1* | #3108 | CTGGCGTCGTGATTAGTGAT / 0.25 | 1× | 138 | NM_000194.3 |
|  | #3109 | CTCGAGCAAGACGTTCAGTC / 0.25 |  |  |  |
| *GAPDH* | #119 | AGCCACATCGCTCAGACACC / 0.25 | 1× | 302 | NM_002046.7, NM_001289745.3, NM_001289746.2, NM_001357943.2 |
|  | #120 | GTACTCAGCGCCAGCATCG / 0.25 |  |  |  |

**Supplementary Table S35**. Thermocycler program used in the RT-qPCR analysis of P53 responsive and non-responsive genes (**Figures 7** and **8**).

| Steps | Temperatures | Times |
| --- | --- | --- |
| Initial denaturation | 95.0 ℃ | 5 min |
| Denaturation | 95.0 ℃ | 10 sec |
| Annealing | 60.0 ℃ | 30 sec |
| Elongation |  |  |
| Plate read |  | |
| Cycles (Go to step 2) | 45 | |
| Melt curve analysis | 65.0 ℃ to 95.0 ℃ (increase in 0.5 ℃ increments with a hold time of 5 sec for each read) | |
| Plate read |  | |

**Supplementary Table S36**. Overview of the antibodies used in the confocal microscopy analyses (**Figure 9D** and **9E**).

| Experiments | Primary antibodies | Secondary antibodies |
| --- | --- | --- |
| *OCT4* gene editing (iPSCs) | 1. Anti-OCT3/4 antibody (1:500; Santa Cruz Biotechnology; Cat. Nr.: SC-5279) | 1. Donkey anti-mouse.IgG Alexa Fluor 555 (1:500; Invitrogen; Cat. Nr.: A31570) |
| Differentiation of *OCT4-*edited iPSC populations | 1. Anti-α Smooth Muscle Actin (α-SMA) antibody (1:500; Sigma-Aldrich; Cat. Nr.: A2547) 2. Anti-Tubulin β 3 (TUBB3) antibody (1:400; Eurogentec; Cat. Nr.:MMS-435P) 3. Anti-HNF3β/FOXA2 antibody (1:100; Sigma-Aldrich; Cat. Nr.: 07-633) 4. Anti-CD31 antibody (1:100; Dako; Cat.Nr.: M0823) | 1. Donkey anti-mouse.IgG Alexa Fluor 555 (1:500; Invitrogen; Cat. Nr.: A31570). 2. Goat anti-rabbit IgG Alexa Fluor 568 (1:500; Invitrogen; Cat. Nr.: A11036). |
